# Supplementary figures and images for: PI3K/HSCB axis facilitates FOG1 nuclear translocation to promote erythropoiesis and megakaryopoiesis (part 1 of 2)
Source: eLife. 2024 May 17;13:RP95815. doi: 10.7554/eLife.95815 (PMC11101173; doi:10.7554/eLife.95815)

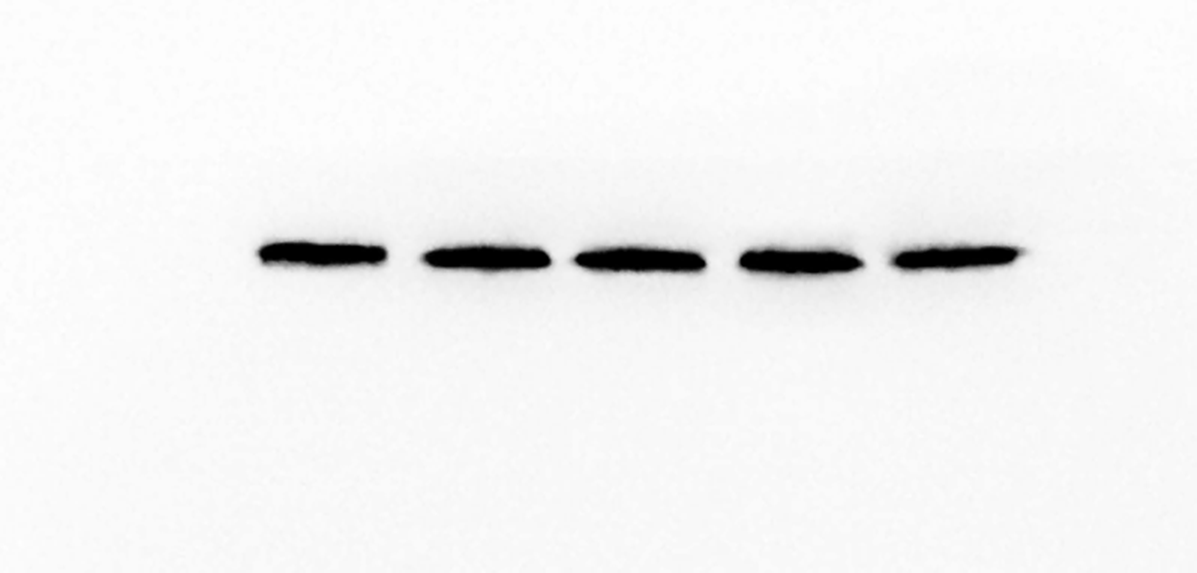

Supplement: Figure 1—source data 1. [file elife-95815-fig1-data1.zip › Figure 1—Source Data 1/Figure 1A Raw WB data/Figure 1A GAPDH.tif]

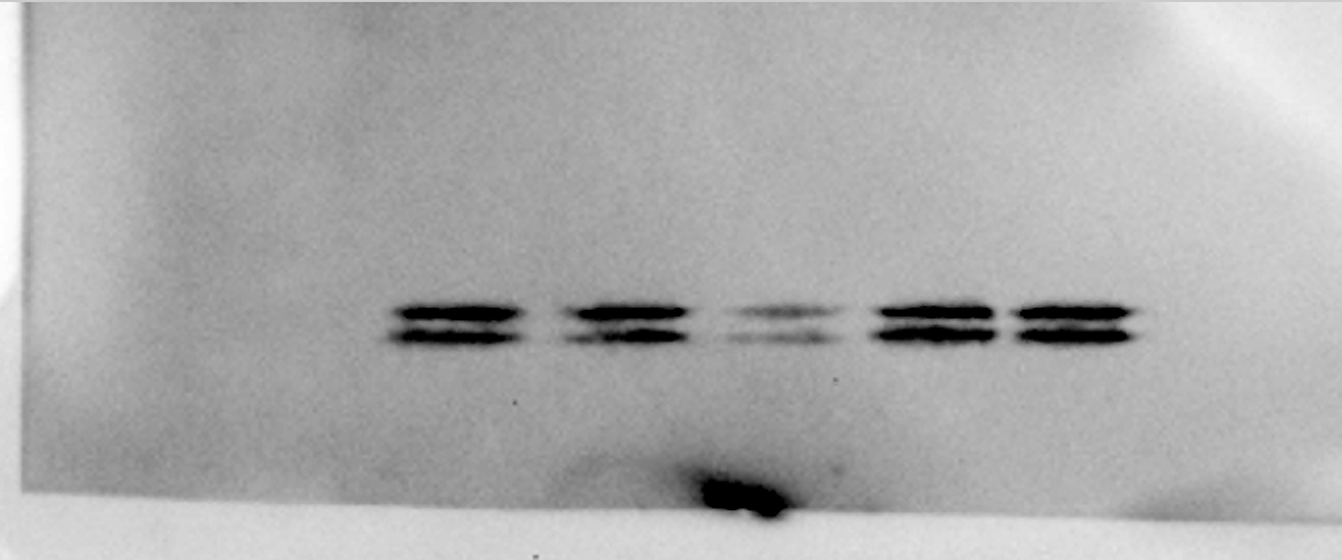

Supplement: Figure 1—source data 1. [file elife-95815-fig1-data1.zip › Figure 1—Source Data 1/Figure 1A Raw WB data/Figure 1A HSCB.tif]

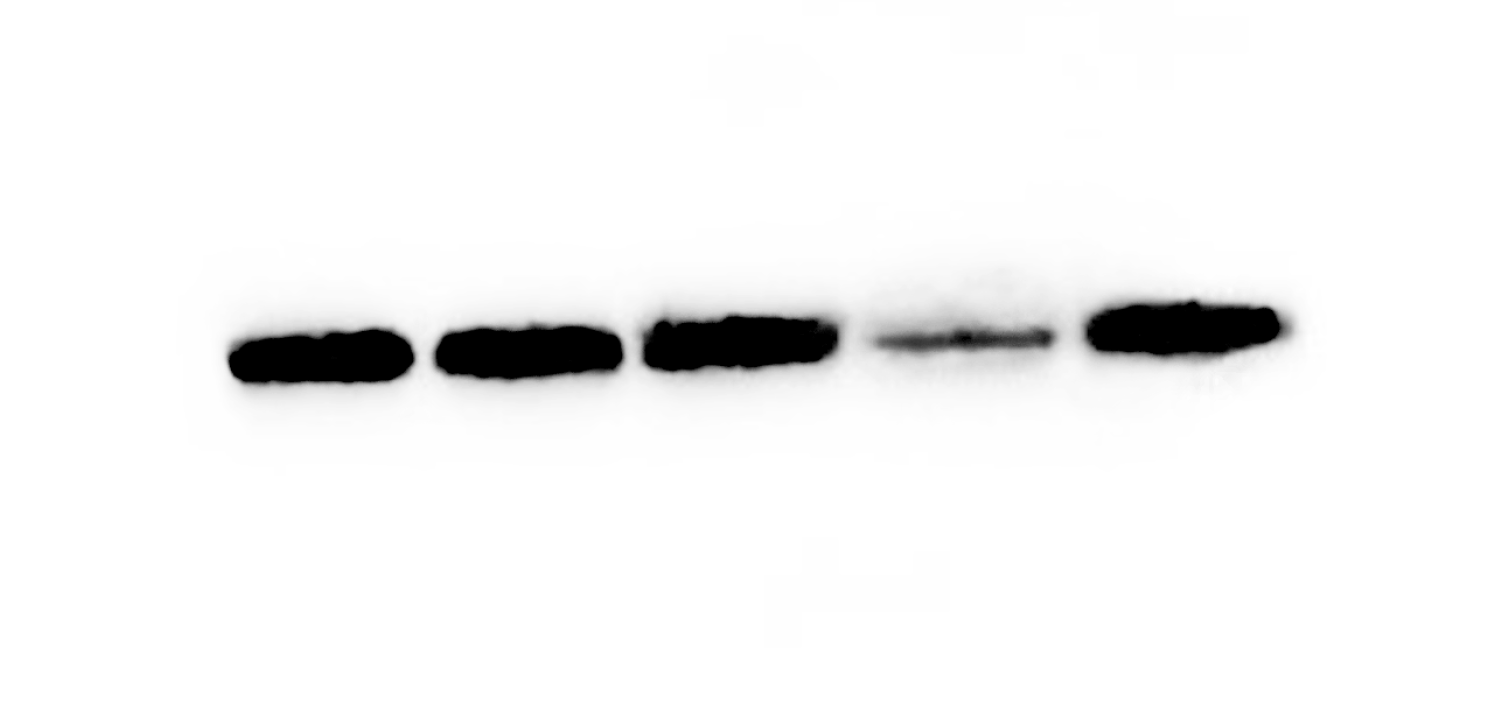

Supplement: Figure 1—source data 1. [file elife-95815-fig1-data1.zip › Figure 1—Source Data 1/Figure 1A Raw WB data/Figure 1A ISCU.tif]

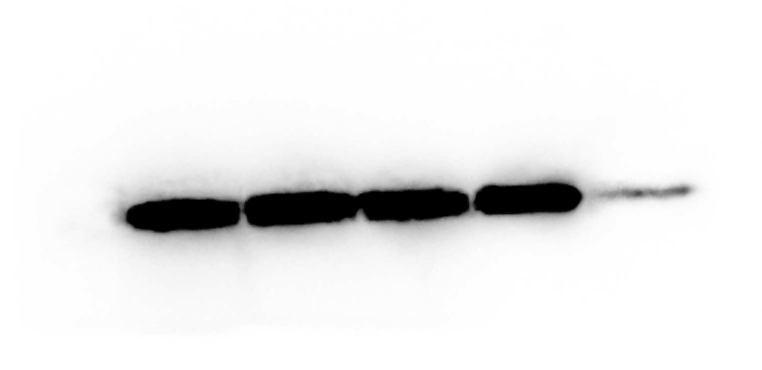

Supplement: Figure 1—source data 1. [file elife-95815-fig1-data1.zip › Figure 1—Source Data 1/Figure 1A Raw WB data/Figure 1A NFS1.tif]

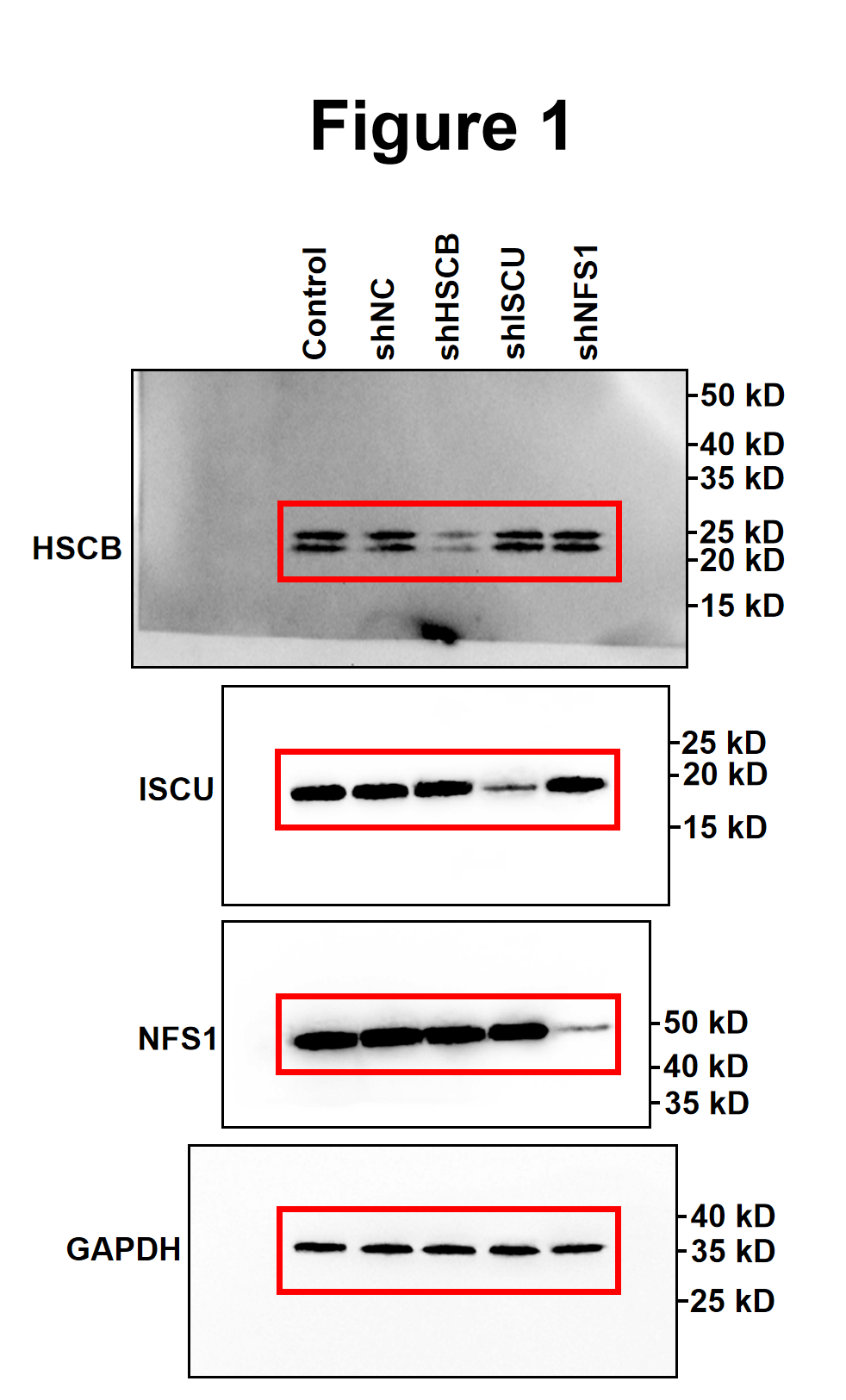

Supplement: Figure 1—source data 1. [file elife-95815-fig1-data1.zip › Figure 1—Source Data 1/Labelled WB data/Source blot data for Figure 1.tif]

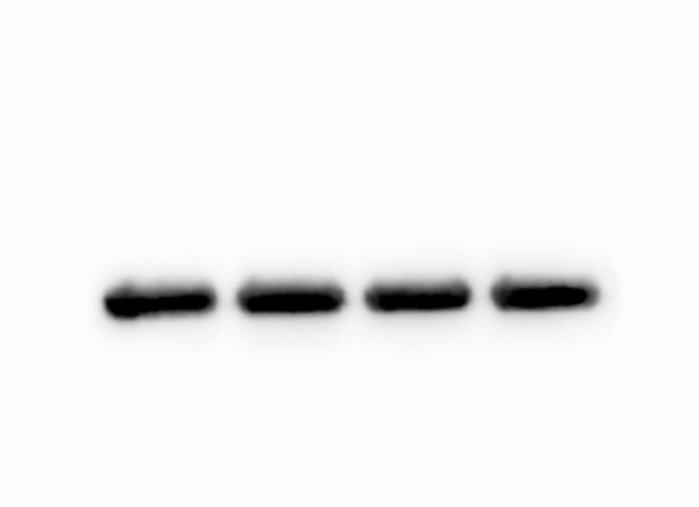

Supplement: Figure 2—source data 1. [file elife-95815-fig2-data1.zip › Figure 2—Source Data 1/Figure 2A Raw WB data/Figure 2A GAPDH.tif]

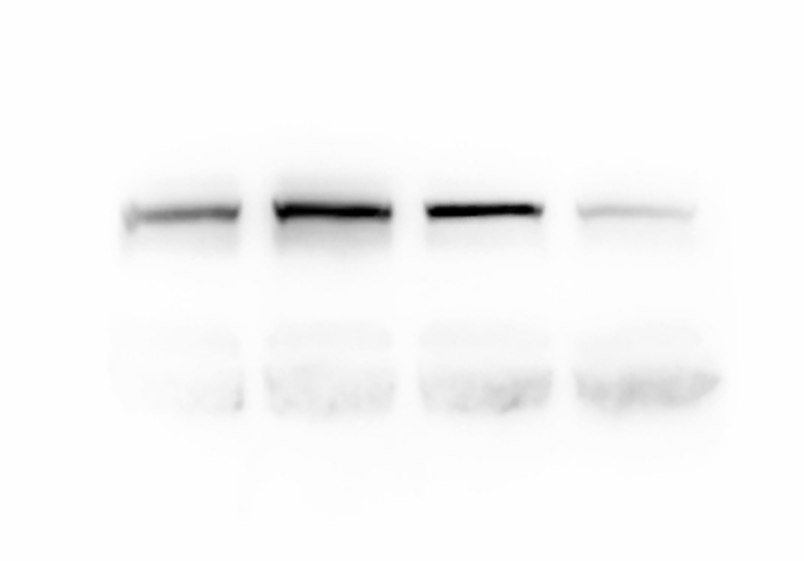

Supplement: Figure 2—source data 1. [file elife-95815-fig2-data1.zip › Figure 2—Source Data 1/Figure 2A Raw WB data/Figure 2A GYPA.tif]

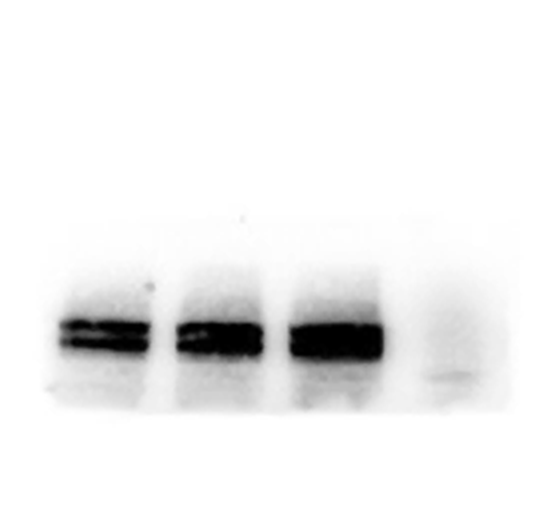

Supplement: Figure 2—source data 1. [file elife-95815-fig2-data1.zip › Figure 2—Source Data 1/Figure 2A Raw WB data/Figure 2A HSCB.tif]

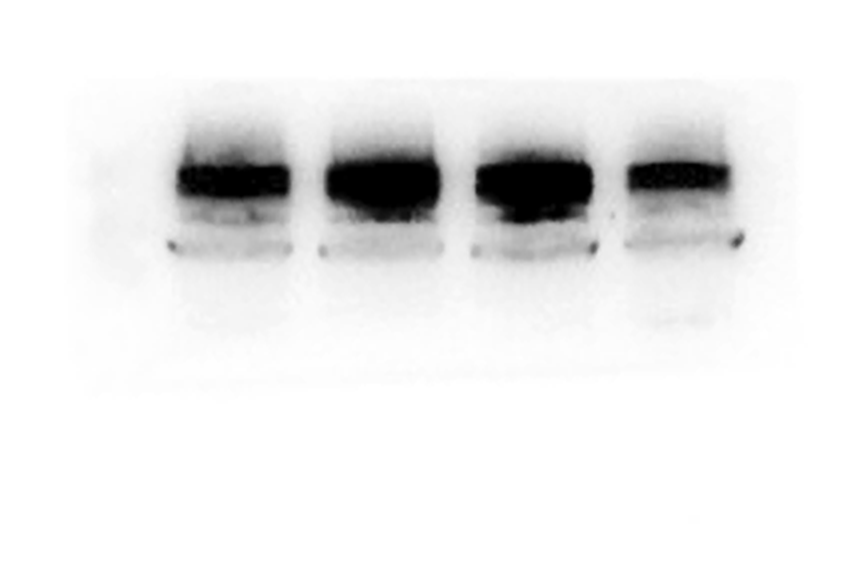

Supplement: Figure 2—source data 1. [file elife-95815-fig2-data1.zip › Figure 2—Source Data 1/Figure 2A Raw WB data/Figure 2A SPTA1.tif]

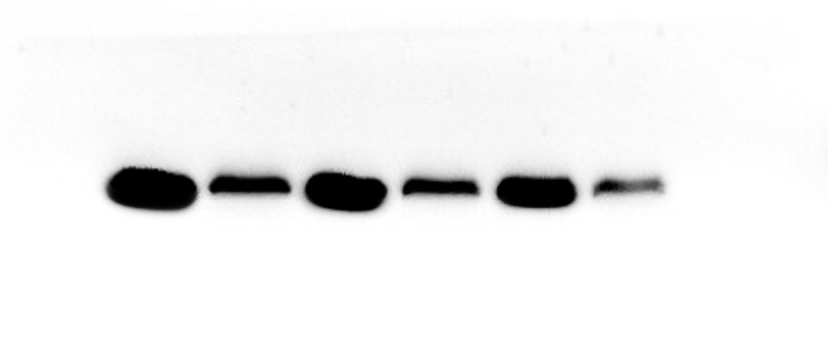

Supplement: Figure 2—source data 1. [file elife-95815-fig2-data1.zip › Figure 2—Source Data 1/Figure 2F Raw WB data/Figure 2F E 2-day FOG1.tif]

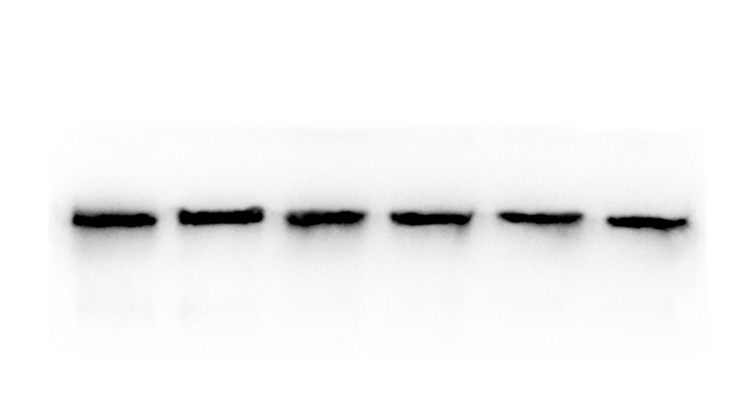

Supplement: Figure 2—source data 1. [file elife-95815-fig2-data1.zip › Figure 2—Source Data 1/Figure 2F Raw WB data/Figure 2F E 2-day GATA1.tif]

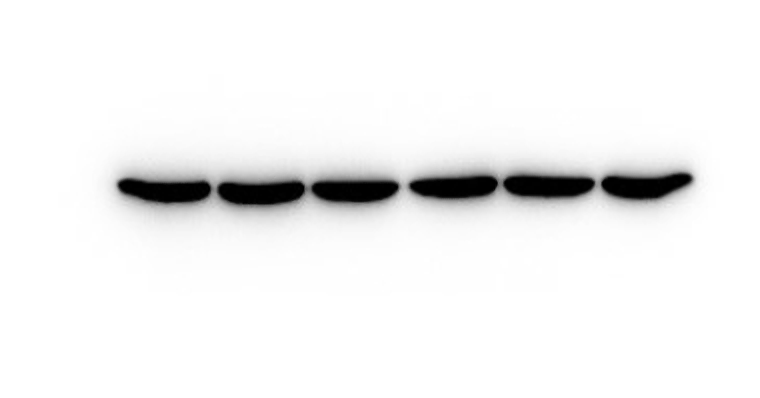

Supplement: Figure 2—source data 1. [file elife-95815-fig2-data1.zip › Figure 2—Source Data 1/Figure 2F Raw WB data/Figure 2F E 2-day LMNB1.tif]

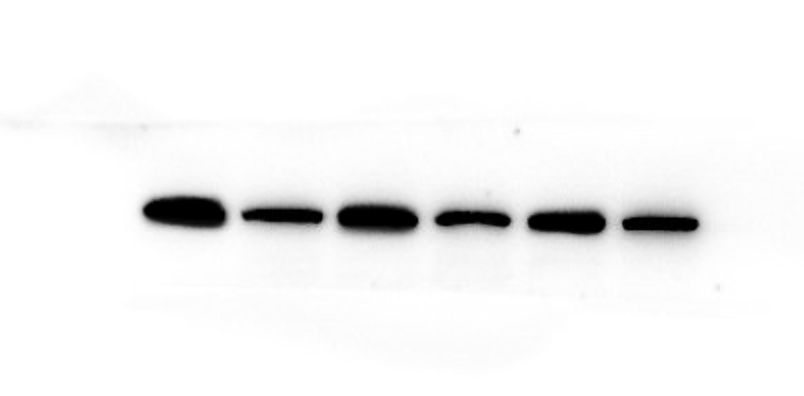

Supplement: Figure 2—source data 1. [file elife-95815-fig2-data1.zip › Figure 2—Source Data 1/Figure 2F Raw WB data/Figure 2F E 2-day LYAR.tif]

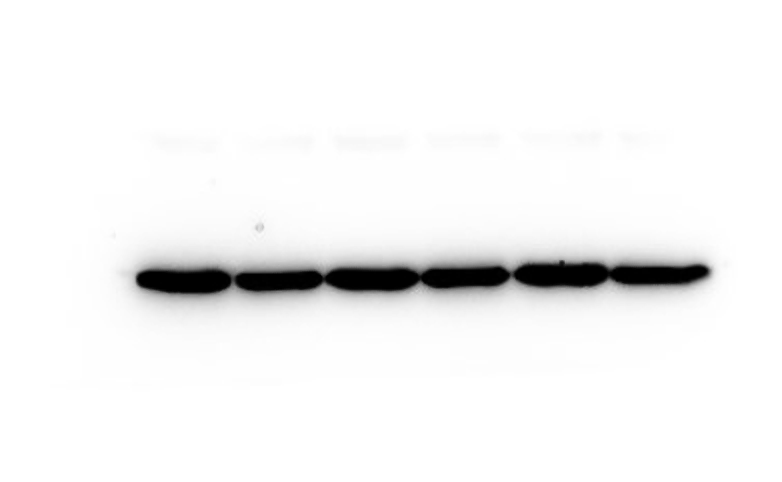

Supplement: Figure 2—source data 1. [file elife-95815-fig2-data1.zip › Figure 2—Source Data 1/Figure 2F Raw WB data/Figure 2F E 2-day NAA30.tif]

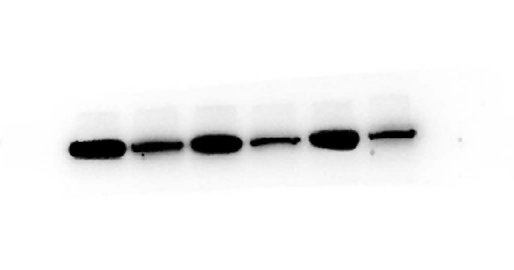

Supplement: Figure 2—source data 1. [file elife-95815-fig2-data1.zip › Figure 2—Source Data 1/Figure 2F Raw WB data/Figure 2F K562 FOG1.tif]

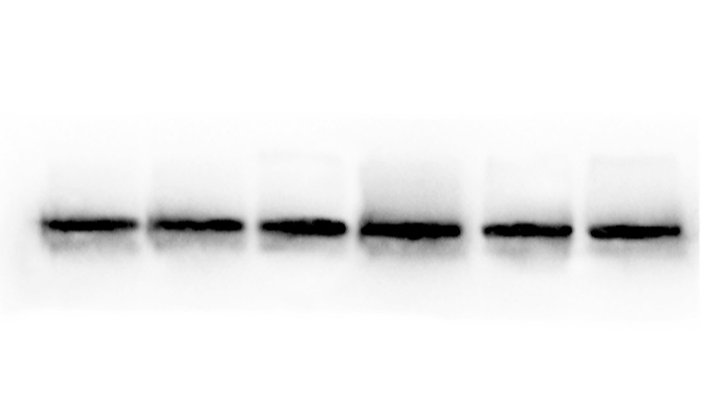

Supplement: Figure 2—source data 1. [file elife-95815-fig2-data1.zip › Figure 2—Source Data 1/Figure 2F Raw WB data/Figure 2F K562 GATA1.tif]

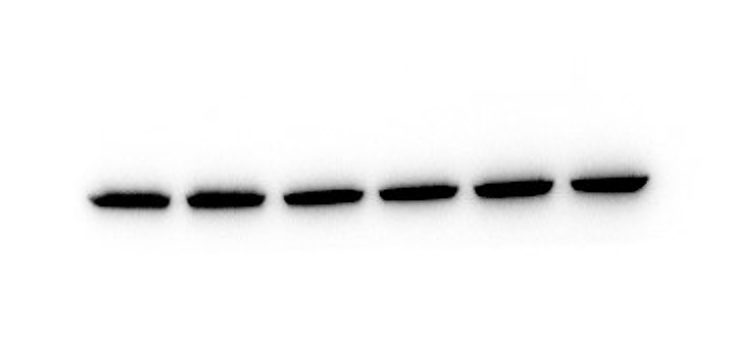

Supplement: Figure 2—source data 1. [file elife-95815-fig2-data1.zip › Figure 2—Source Data 1/Figure 2F Raw WB data/Figure 2F K562 LMNB1.tif]

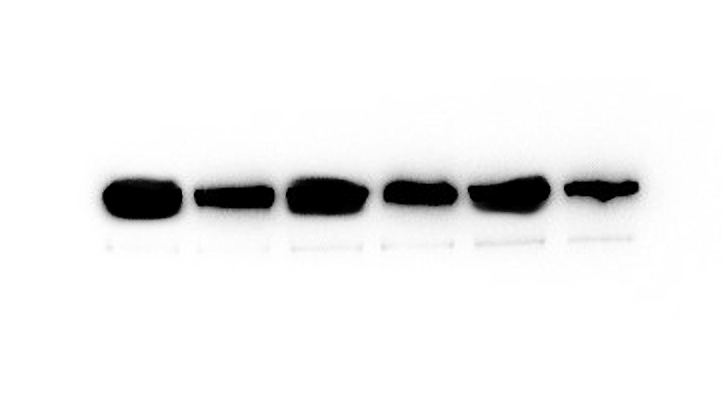

Supplement: Figure 2—source data 1. [file elife-95815-fig2-data1.zip › Figure 2—Source Data 1/Figure 2F Raw WB data/Figure 2F K562 LYAR.tif]

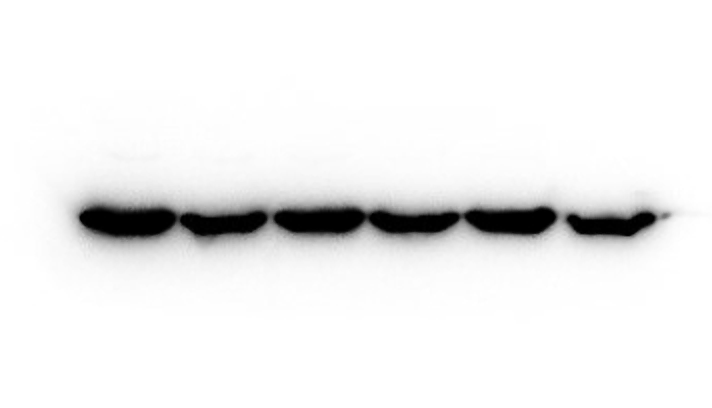

Supplement: Figure 2—source data 1. [file elife-95815-fig2-data1.zip › Figure 2—Source Data 1/Figure 2F Raw WB data/Figure 2F K562 NAA30.tif]

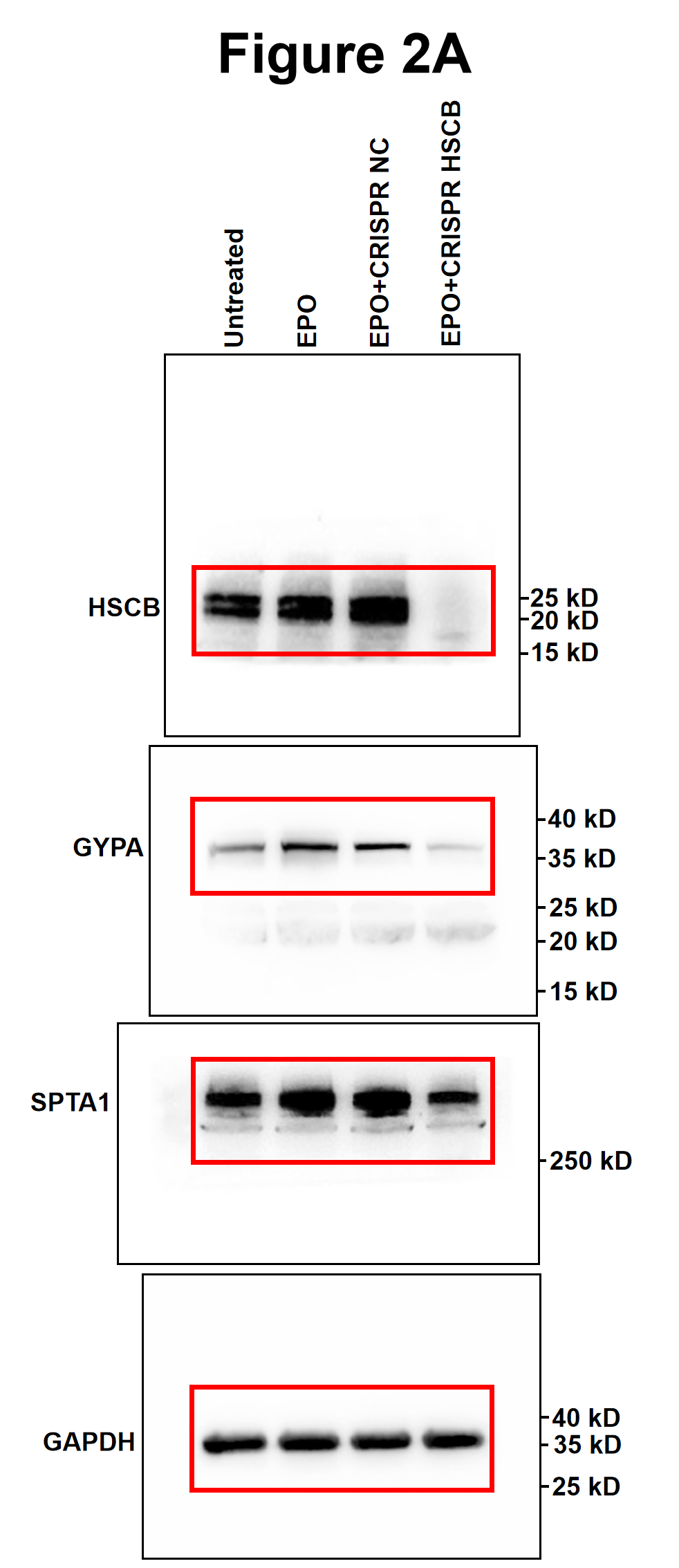

Supplement: Figure 2—source data 1. [file elife-95815-fig2-data1.zip › Figure 2—Source Data 1/Labelled WB data/Source blot data for Figure 2A.tif]

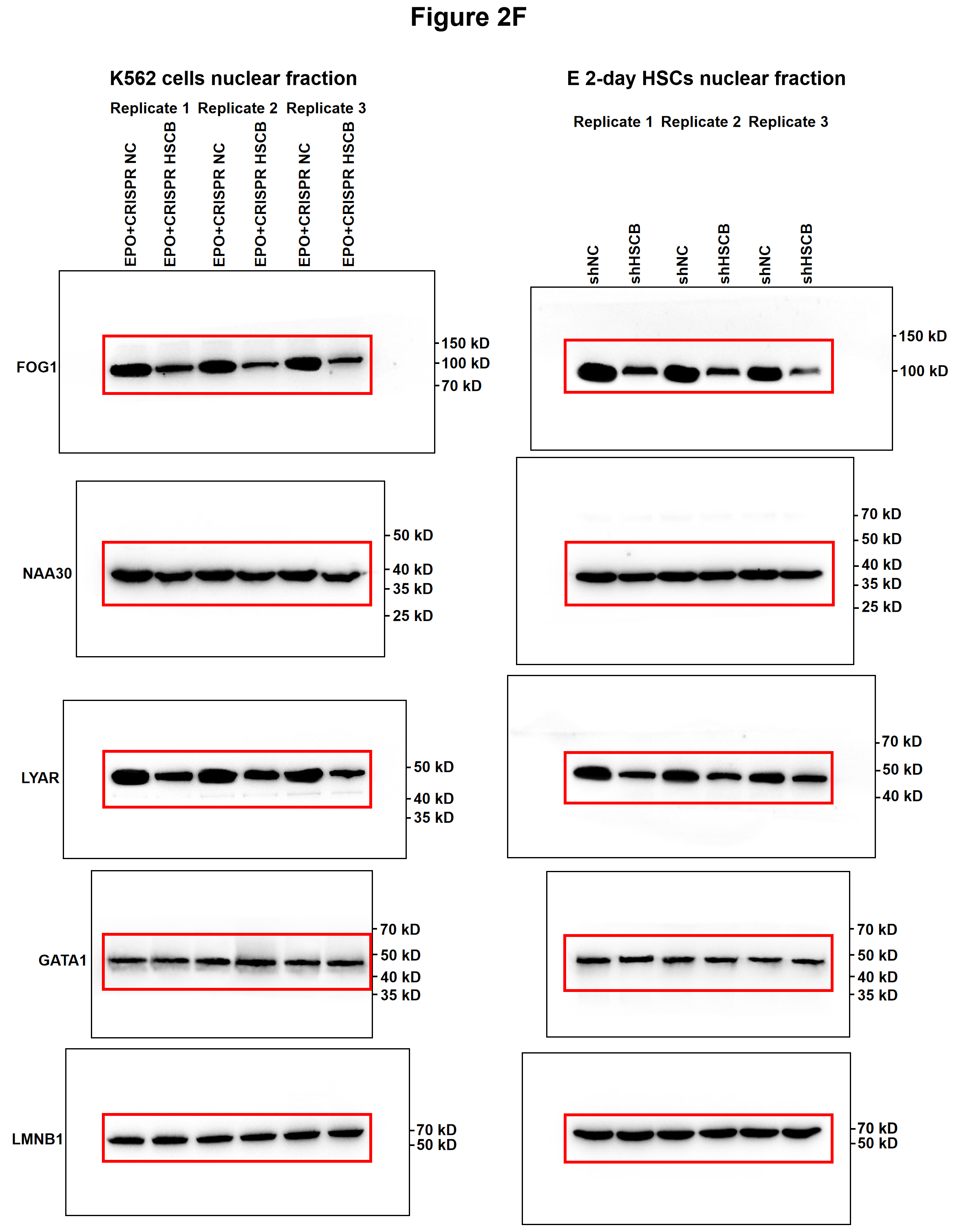

Supplement: Figure 2—source data 1. [file elife-95815-fig2-data1.zip › Figure 2—Source Data 1/Labelled WB data/Source blot data for Figure 2F.tif]

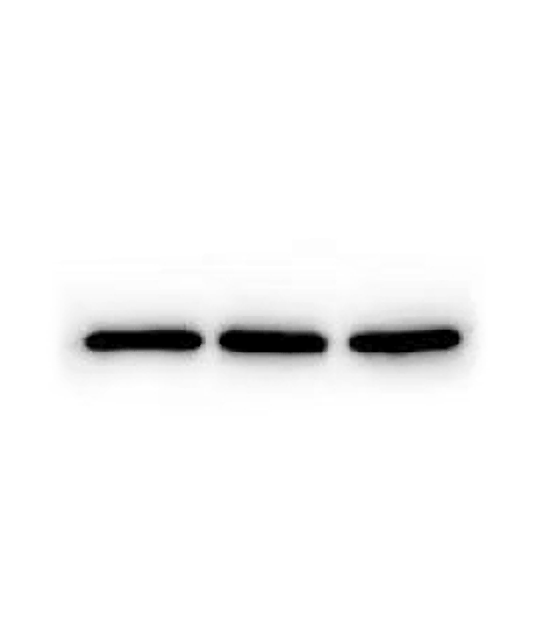

Supplement: Figure 3—source data 1. [file elife-95815-fig3-data1.zip › Figure 3—Source Data 1/Figure 3A Raw WB data/Figure 3A E 2-day GAPDH.tif]

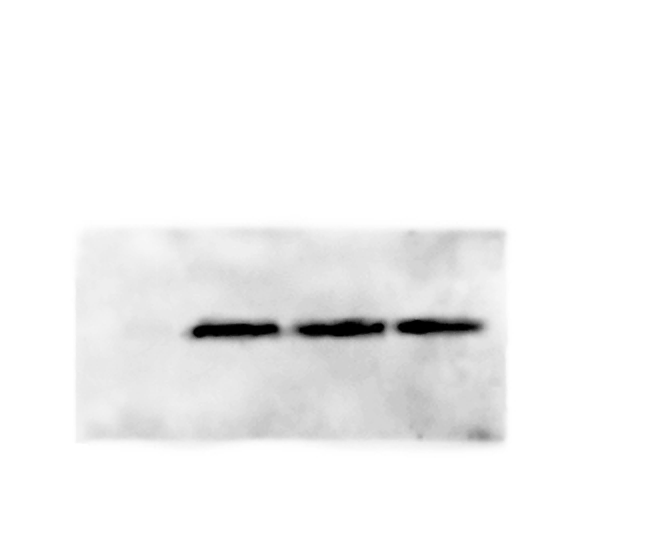

Supplement: Figure 3—source data 1. [file elife-95815-fig3-data1.zip › Figure 3—Source Data 1/Figure 3A Raw WB data/Figure 3A E 2-day GATA1.tif]

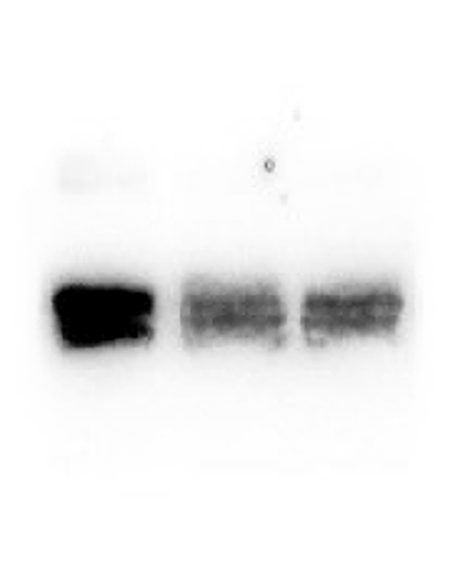

Supplement: Figure 3—source data 1. [file elife-95815-fig3-data1.zip › Figure 3—Source Data 1/Figure 3A Raw WB data/Figure 3A E 2-day HSCB.tif]

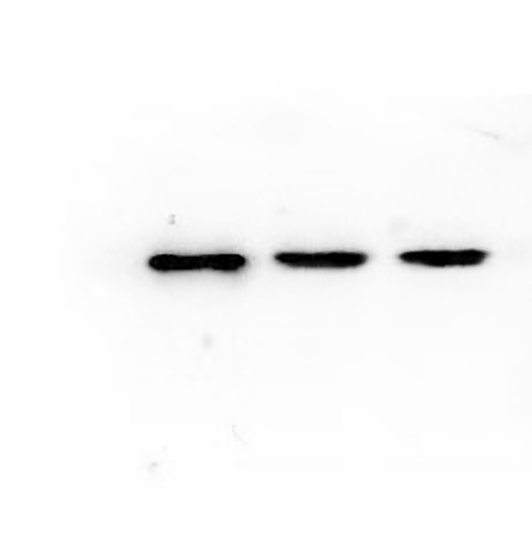

Supplement: Figure 3—source data 1. [file elife-95815-fig3-data1.zip › Figure 3—Source Data 1/Figure 3A Raw WB data/Figure 3A E 2-day LMNB1.tif]

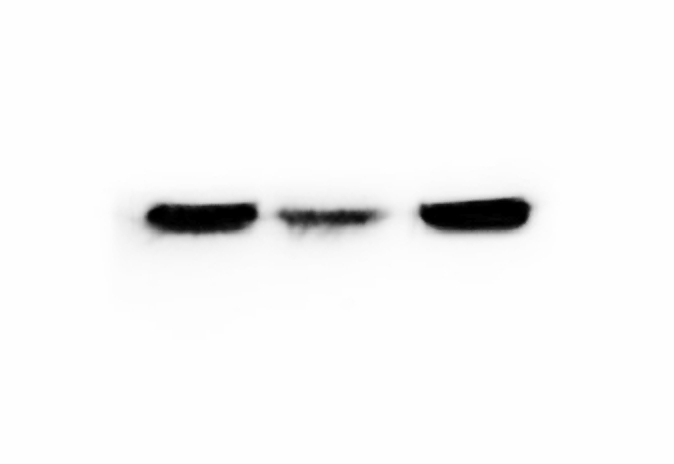

Supplement: Figure 3—source data 1. [file elife-95815-fig3-data1.zip › Figure 3—Source Data 1/Figure 3A Raw WB data/Figure 3A E 2-day N-FOG1.tif]

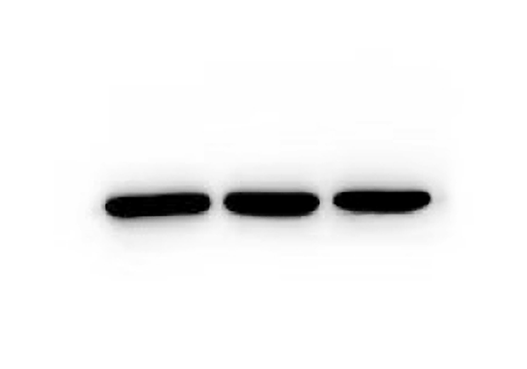

Supplement: Figure 3—source data 1. [file elife-95815-fig3-data1.zip › Figure 3—Source Data 1/Figure 3A Raw WB data/Figure 3A K562 GAPDH.tif]

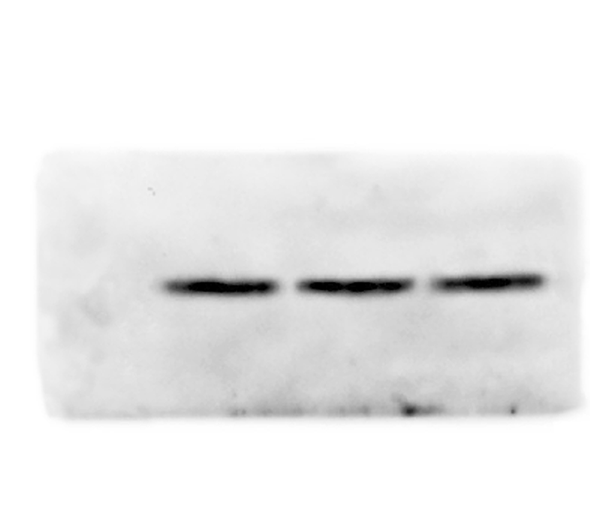

Supplement: Figure 3—source data 1. [file elife-95815-fig3-data1.zip › Figure 3—Source Data 1/Figure 3A Raw WB data/Figure 3A K562 GATA1.tif]

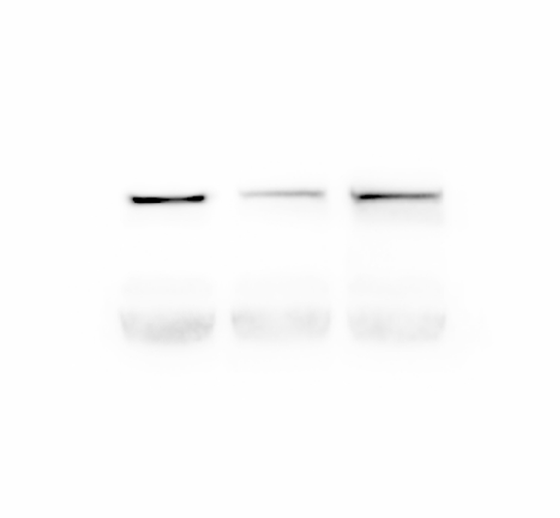

Supplement: Figure 3—source data 1. [file elife-95815-fig3-data1.zip › Figure 3—Source Data 1/Figure 3A Raw WB data/Figure 3A K562 GYPA.tif]

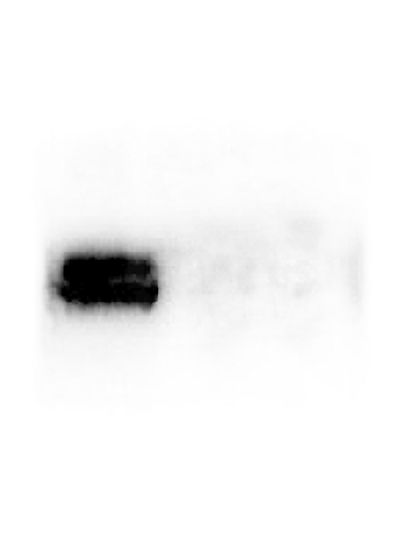

Supplement: Figure 3—source data 1. [file elife-95815-fig3-data1.zip › Figure 3—Source Data 1/Figure 3A Raw WB data/Figure 3A K562 HSCB.tif]

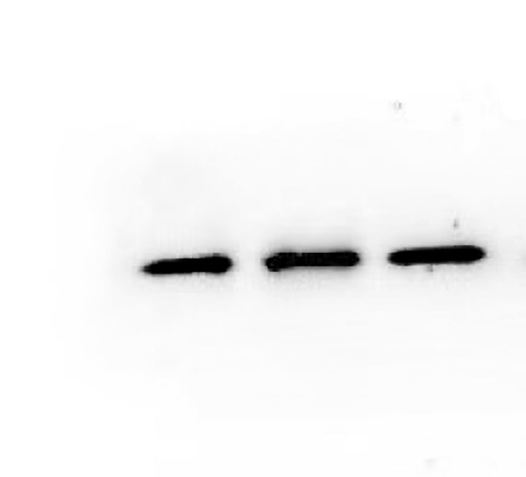

Supplement: Figure 3—source data 1. [file elife-95815-fig3-data1.zip › Figure 3—Source Data 1/Figure 3A Raw WB data/Figure 3A K562 LMNB1.tif]

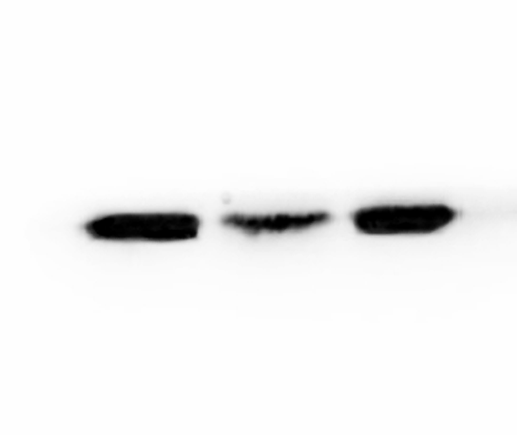

Supplement: Figure 3—source data 1. [file elife-95815-fig3-data1.zip › Figure 3—Source Data 1/Figure 3A Raw WB data/Figure 3A K562 N-FOG1.tif]

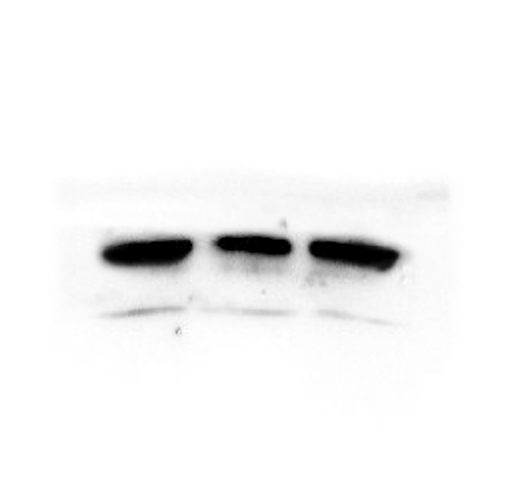

Supplement: Figure 3—source data 1. [file elife-95815-fig3-data1.zip › Figure 3—Source Data 1/Figure 3A Raw WB data/Figure 3A K562 SPTA1.tif]

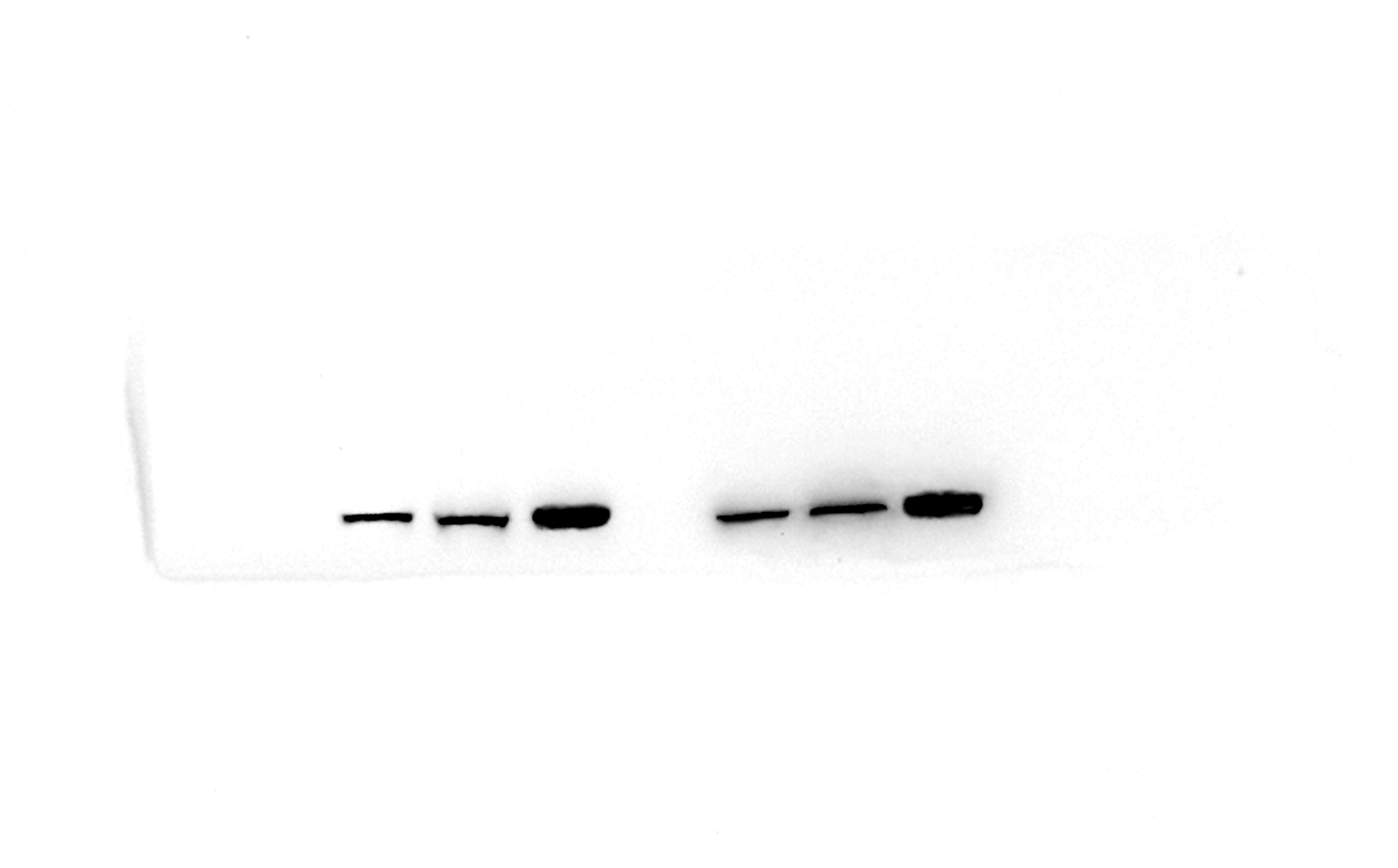

Supplement: Figure 3—source data 1. [file elife-95815-fig3-data1.zip › Figure 3—Source Data 1/Figure 3A Raw WB data/Figure 3A total FOG1.tif]

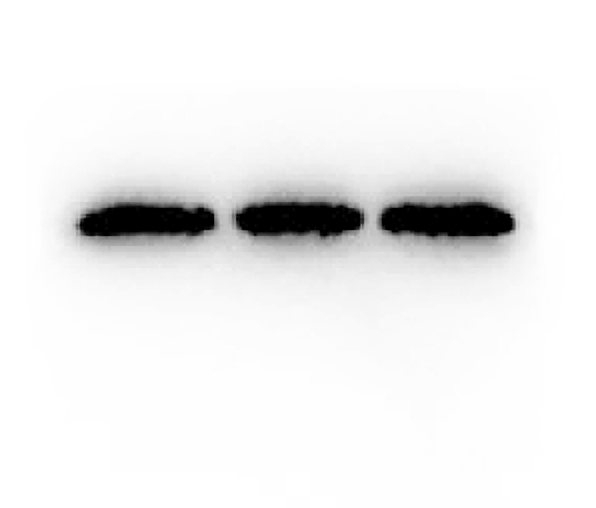

Supplement: Figure 3—source data 1. [file elife-95815-fig3-data1.zip › Figure 3—Source Data 1/Figure 3F Raw WB data/Figure 3F E 2-day ACTB.tif]

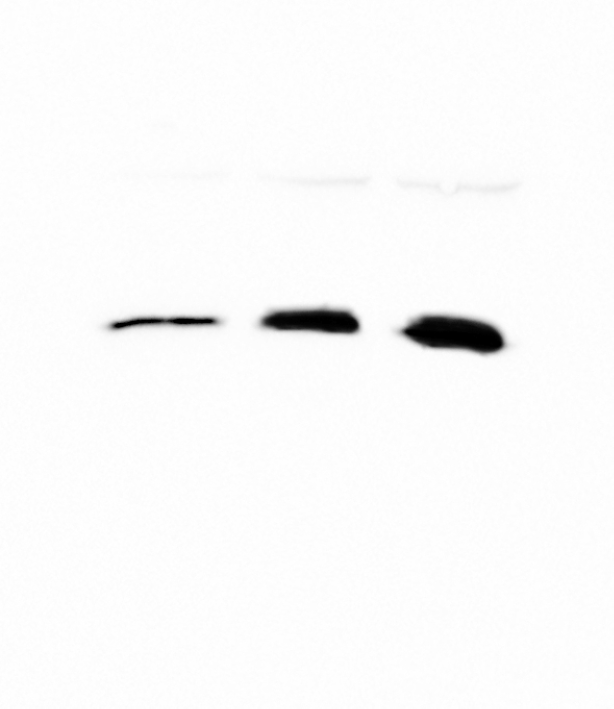

Supplement: Figure 3—source data 1. [file elife-95815-fig3-data1.zip › Figure 3—Source Data 1/Figure 3F Raw WB data/Figure 3F E 2-day C-FOG1.tif]

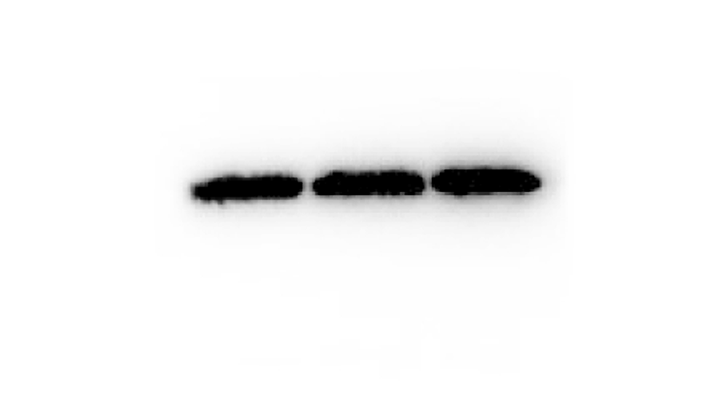

Supplement: Figure 3—source data 1. [file elife-95815-fig3-data1.zip › Figure 3—Source Data 1/Figure 3F Raw WB data/Figure 3F K562 ACTB.tif]

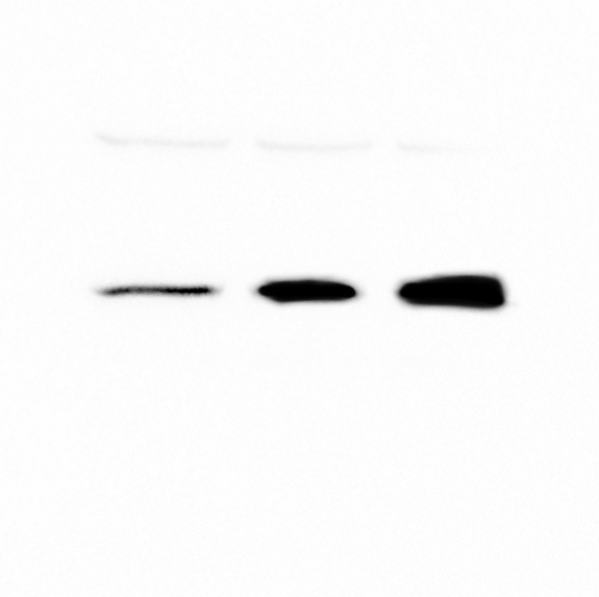

Supplement: Figure 3—source data 1. [file elife-95815-fig3-data1.zip › Figure 3—Source Data 1/Figure 3F Raw WB data/Figure 3F K562 C-FOG1.tif]

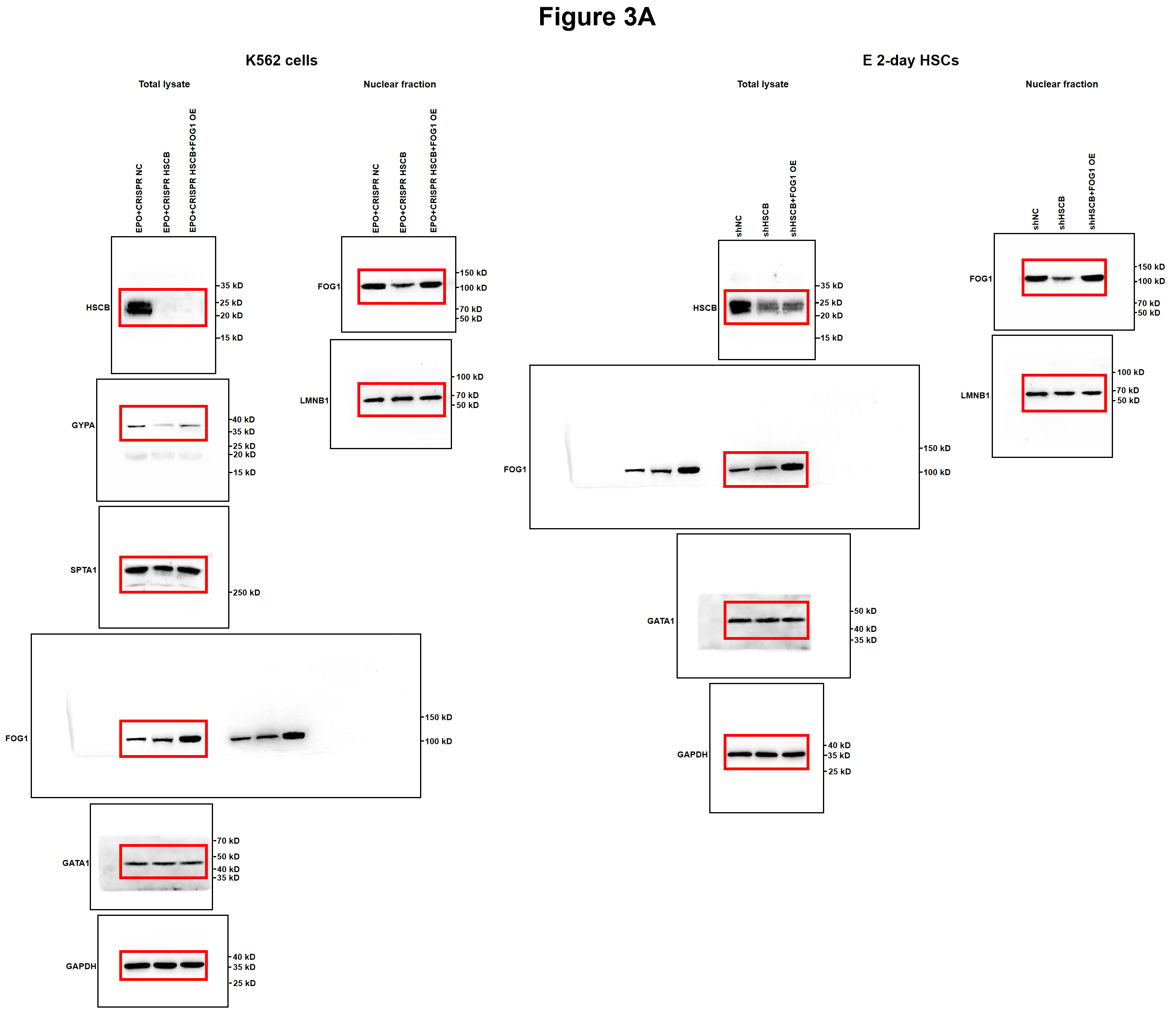

Supplement: Figure 3—source data 1. [file elife-95815-fig3-data1.zip › Figure 3—Source Data 1/Labelled WB data/Source blot data for Figure 3A.tif]

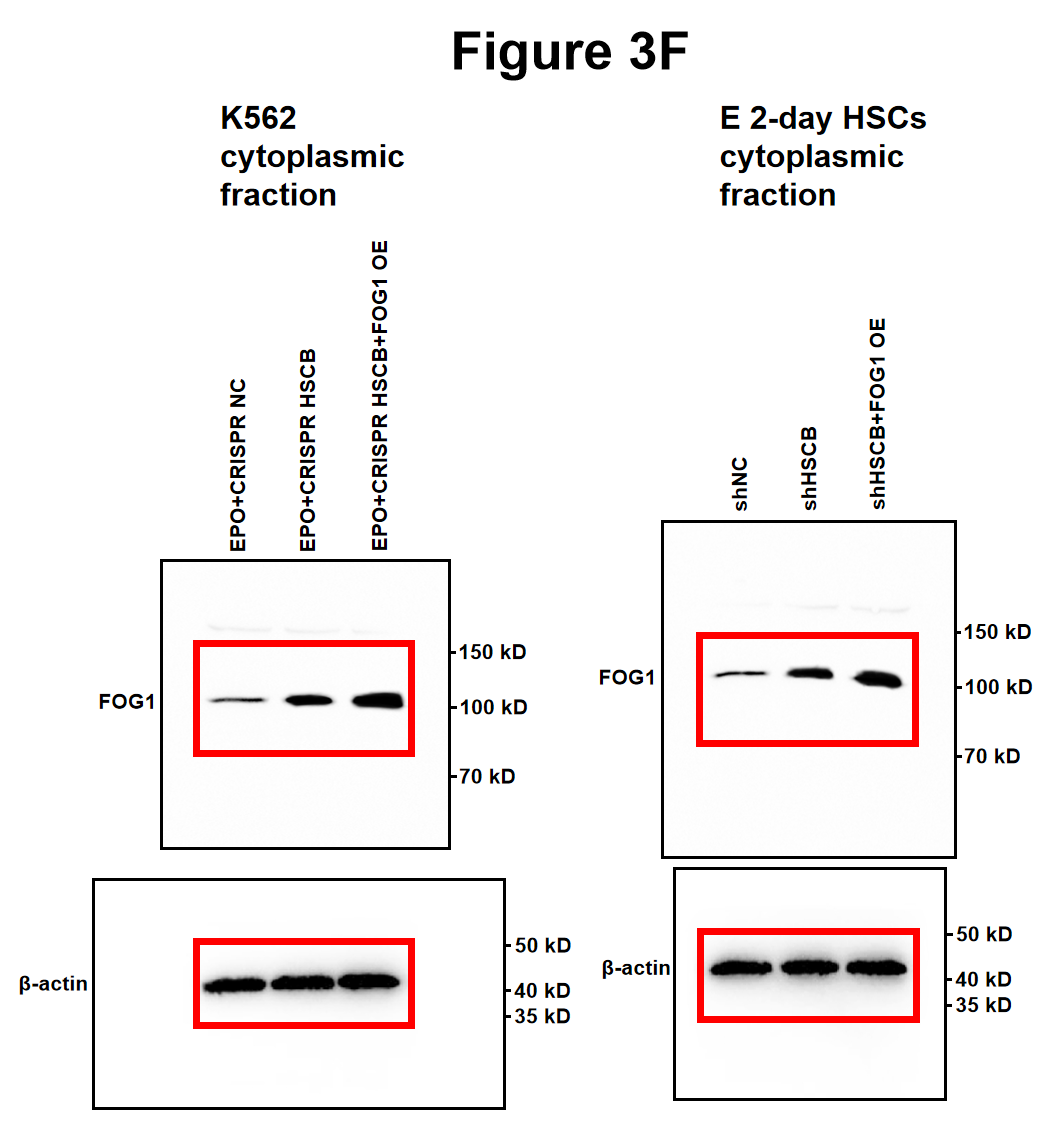

Supplement: Figure 3—source data 1. [file elife-95815-fig3-data1.zip › Figure 3—Source Data 1/Labelled WB data/Source blot data for Figure 3F.tif]

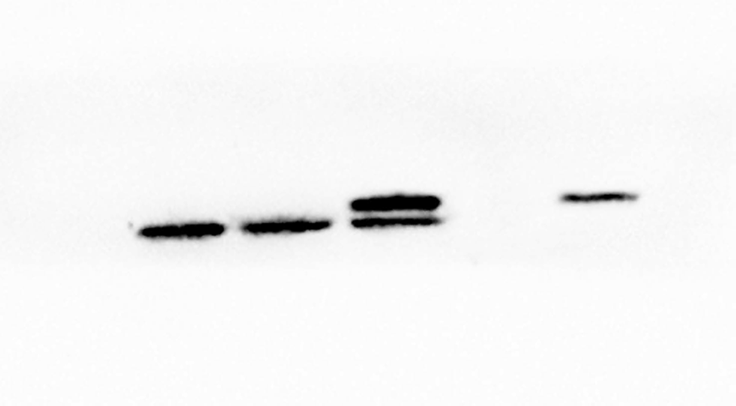

Supplement: Figure 4—source data 1. [file elife-95815-fig4-data1.zip › Figure 4—Source Data 1/Figure 4B Raw WB data/Figure 4B EPO+CRISPR HSCB FOG1.tif]

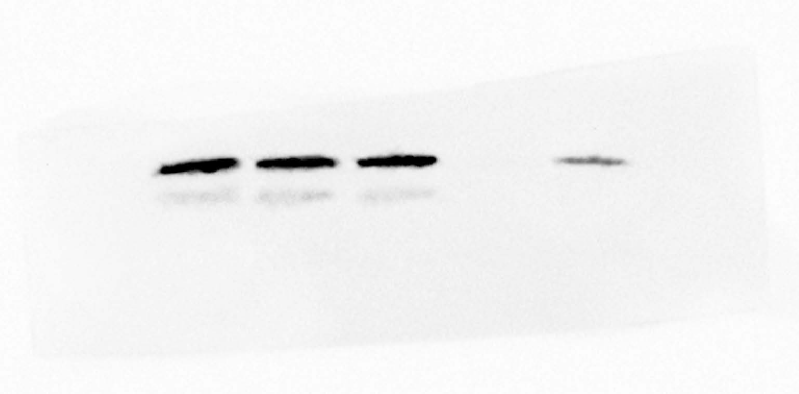

Supplement: Figure 4—source data 1. [file elife-95815-fig4-data1.zip › Figure 4—Source Data 1/Figure 4B Raw WB data/Figure 4B EPO+CRISPR HSCB TACC3.tif]

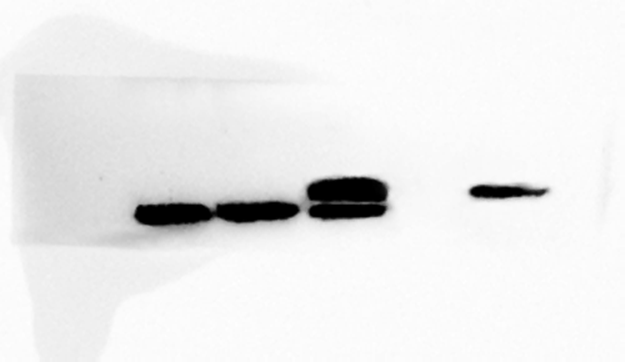

Supplement: Figure 4—source data 1. [file elife-95815-fig4-data1.zip › Figure 4—Source Data 1/Figure 4B Raw WB data/Figure 4B EPO+CRISPR NC FOG1.tif]

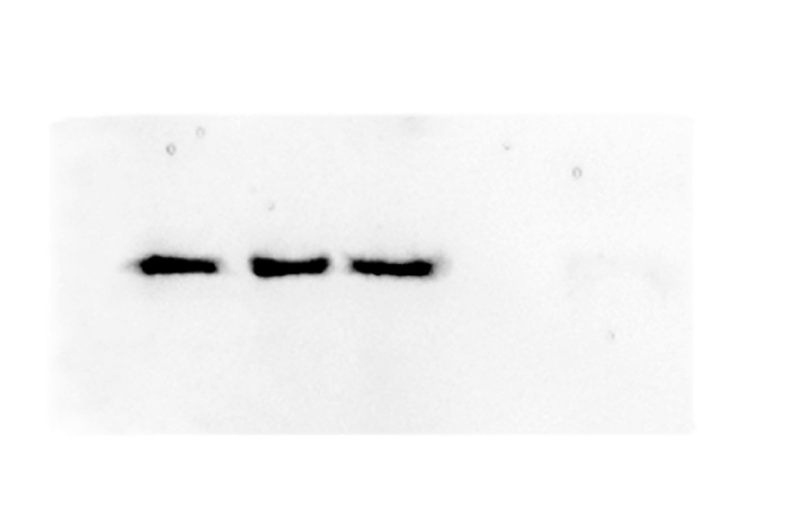

Supplement: Figure 4—source data 1. [file elife-95815-fig4-data1.zip › Figure 4—Source Data 1/Figure 4B Raw WB data/Figure 4B EPO+CRISPR NC TACC3.tif]

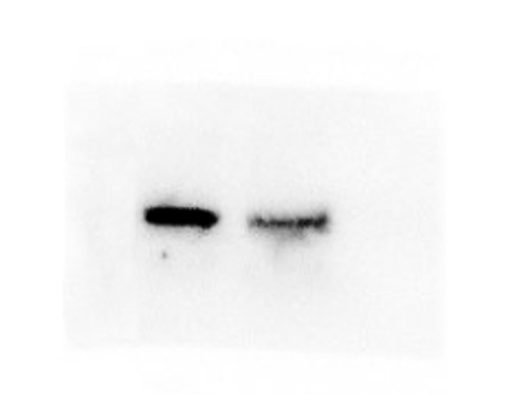

Supplement: Figure 4—source data 1. [file elife-95815-fig4-data1.zip › Figure 4—Source Data 1/Figure 4C Raw WB data/Figure 4C shHSCB FOG1.tif]

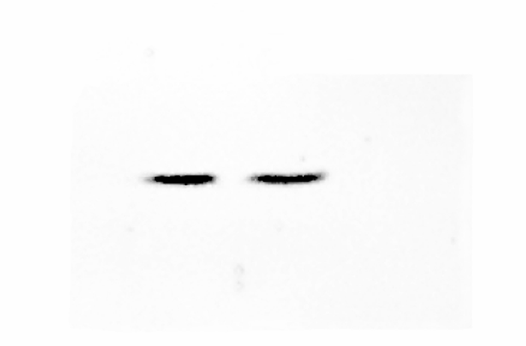

Supplement: Figure 4—source data 1. [file elife-95815-fig4-data1.zip › Figure 4—Source Data 1/Figure 4C Raw WB data/Figure 4C shHSCB TACC3.tif]

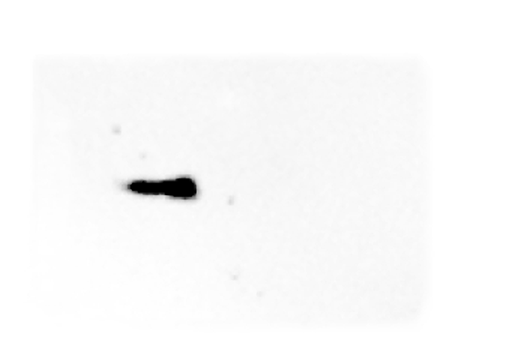

Supplement: Figure 4—source data 1. [file elife-95815-fig4-data1.zip › Figure 4—Source Data 1/Figure 4C Raw WB data/Figure 4C shNC FOG1.tif]

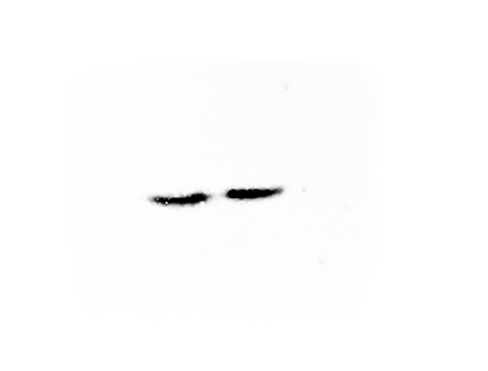

Supplement: Figure 4—source data 1. [file elife-95815-fig4-data1.zip › Figure 4—Source Data 1/Figure 4C Raw WB data/Figure 4C shNC TACC3.tif]

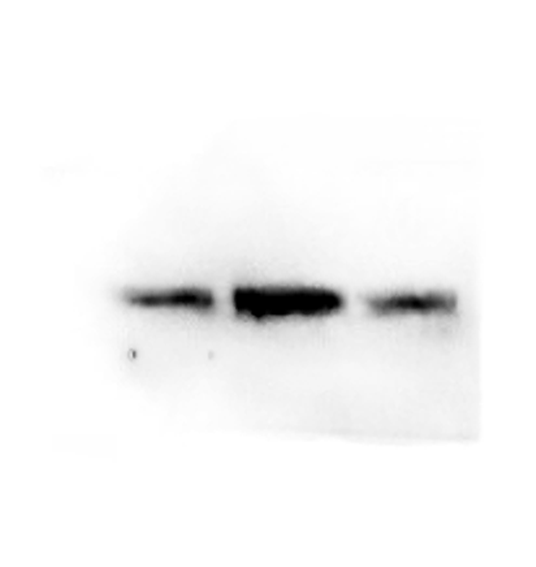

Supplement: Figure 4—source data 1. [file elife-95815-fig4-data1.zip › Figure 4—Source Data 1/Figure 4D Raw WB data/Figure 4D E 2-day C-FOG1.tif]

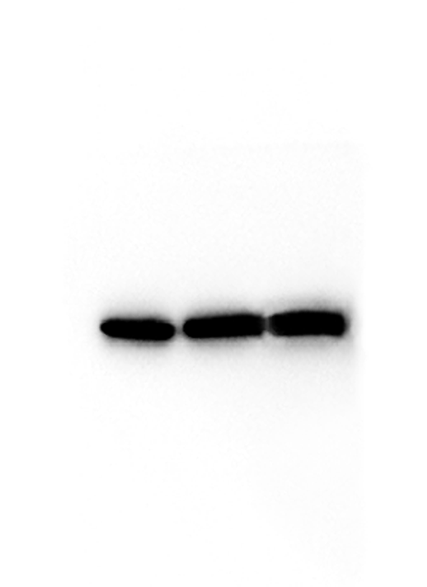

Supplement: Figure 4—source data 1. [file elife-95815-fig4-data1.zip › Figure 4—Source Data 1/Figure 4D Raw WB data/Figure 4D E 2-day C-GAPDH.tif]

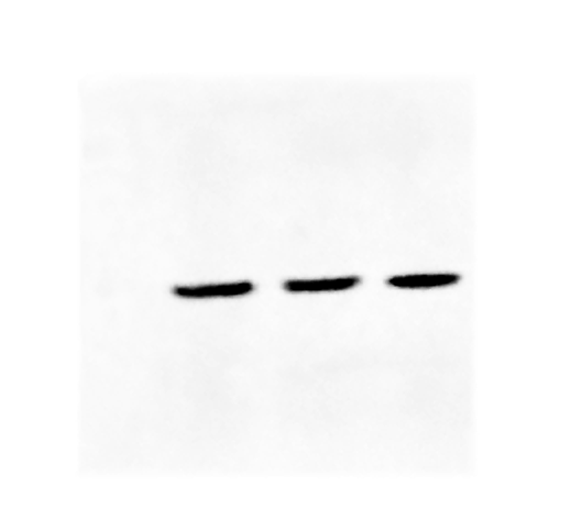

Supplement: Figure 4—source data 1. [file elife-95815-fig4-data1.zip › Figure 4—Source Data 1/Figure 4D Raw WB data/Figure 4D E 2-day FOG1.tif]

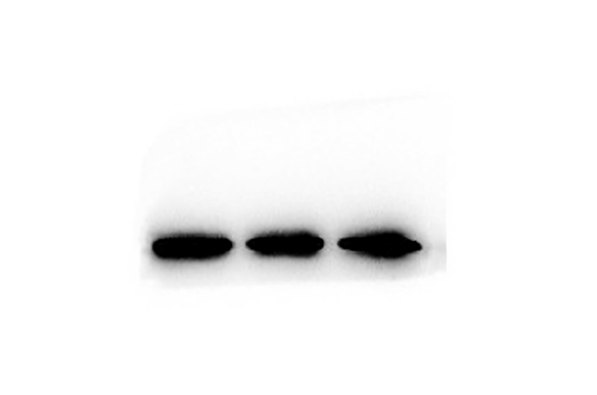

Supplement: Figure 4—source data 1. [file elife-95815-fig4-data1.zip › Figure 4—Source Data 1/Figure 4D Raw WB data/Figure 4D E 2-day GAPDH.tif]

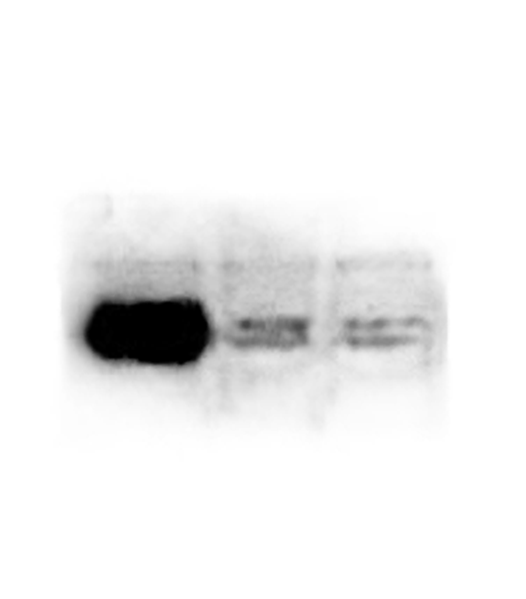

Supplement: Figure 4—source data 1. [file elife-95815-fig4-data1.zip › Figure 4—Source Data 1/Figure 4D Raw WB data/Figure 4D E 2-day HSCB.tif]

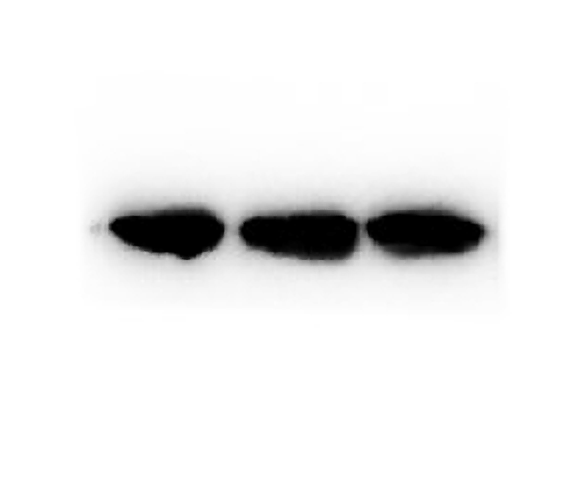

Supplement: Figure 4—source data 1. [file elife-95815-fig4-data1.zip › Figure 4—Source Data 1/Figure 4D Raw WB data/Figure 4D E 2-day LMNB1.tif]

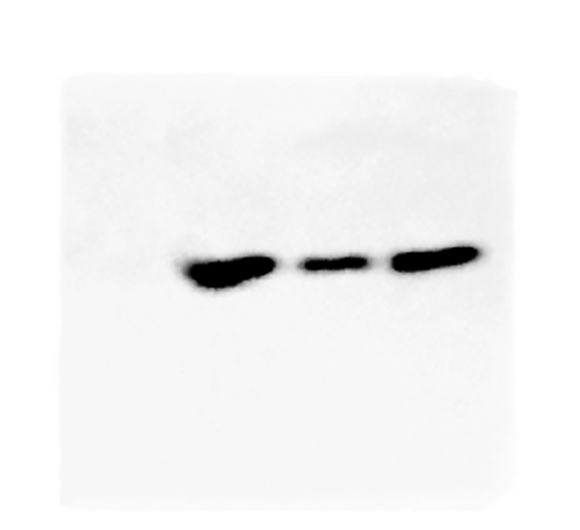

Supplement: Figure 4—source data 1. [file elife-95815-fig4-data1.zip › Figure 4—Source Data 1/Figure 4D Raw WB data/Figure 4D E 2-day N-FOG1.tif]

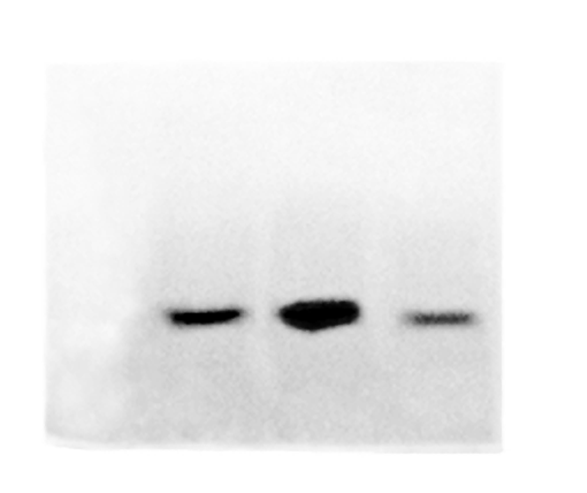

Supplement: Figure 4—source data 1. [file elife-95815-fig4-data1.zip › Figure 4—Source Data 1/Figure 4D Raw WB data/Figure 4D E 2-day TACC3.tif]

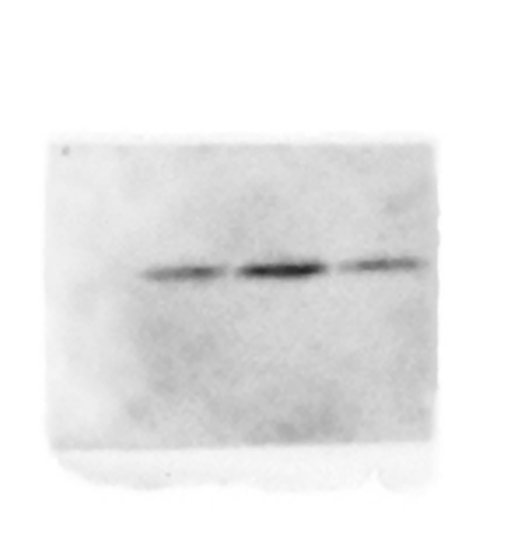

Supplement: Figure 4—source data 1. [file elife-95815-fig4-data1.zip › Figure 4—Source Data 1/Figure 4D Raw WB data/Figure 4D K562 C-FOG1.tif]

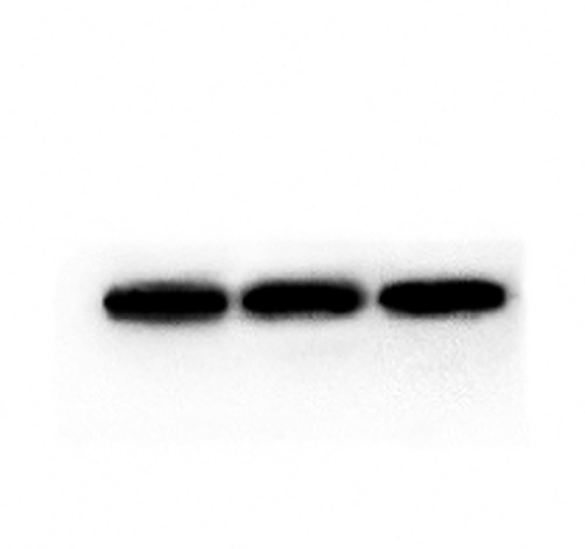

Supplement: Figure 4—source data 1. [file elife-95815-fig4-data1.zip › Figure 4—Source Data 1/Figure 4D Raw WB data/Figure 4D K562 C-GAPDH.tif]

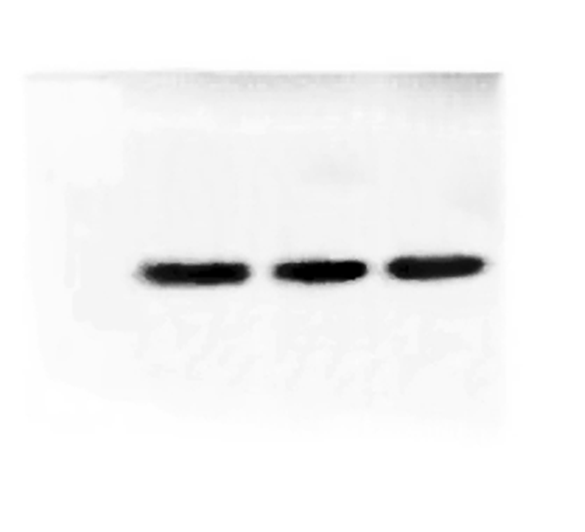

Supplement: Figure 4—source data 1. [file elife-95815-fig4-data1.zip › Figure 4—Source Data 1/Figure 4D Raw WB data/Figure 4D K562 FOG1.tif]

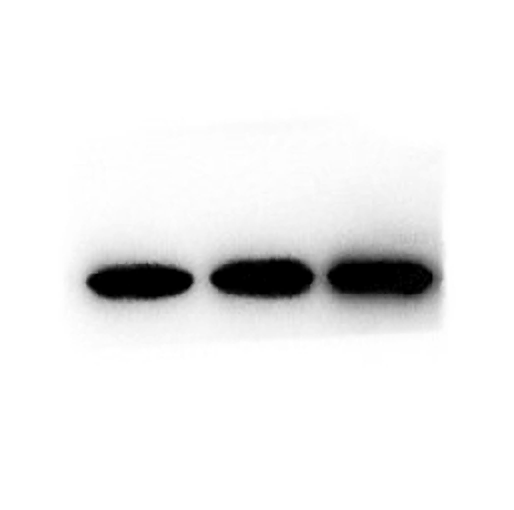

Supplement: Figure 4—source data 1. [file elife-95815-fig4-data1.zip › Figure 4—Source Data 1/Figure 4D Raw WB data/Figure 4D K562 GAPDH.tif]

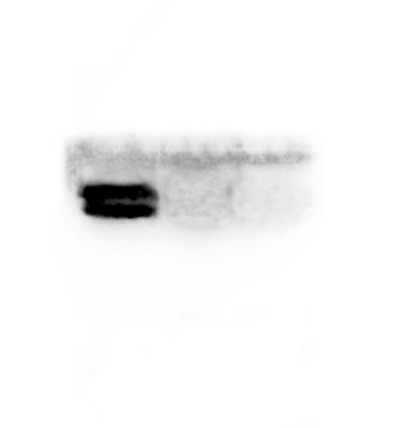

Supplement: Figure 4—source data 1. [file elife-95815-fig4-data1.zip › Figure 4—Source Data 1/Figure 4D Raw WB data/Figure 4D K562 HSCB.tif]

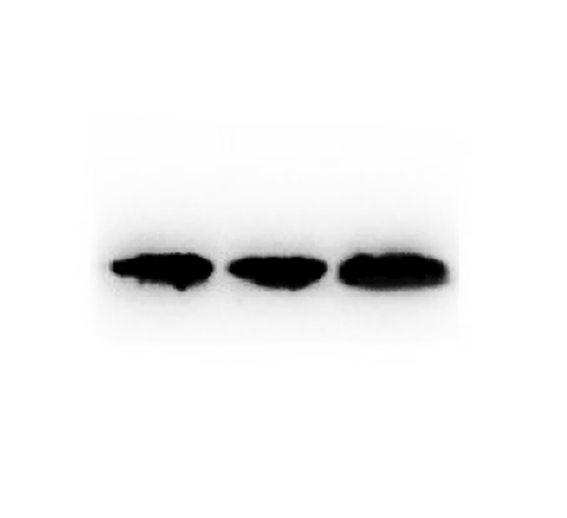

Supplement: Figure 4—source data 1. [file elife-95815-fig4-data1.zip › Figure 4—Source Data 1/Figure 4D Raw WB data/Figure 4D K562 LMNB1.tif]

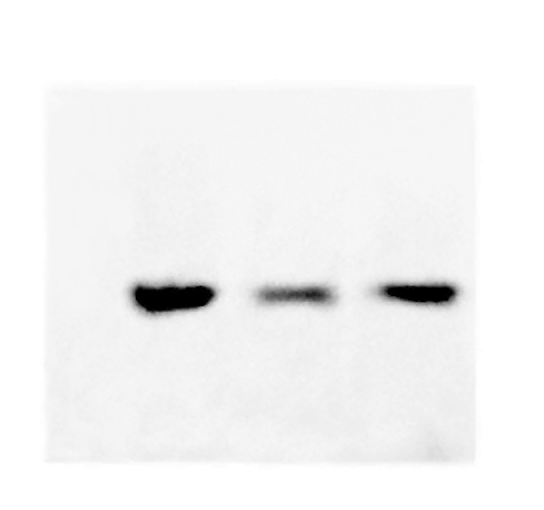

Supplement: Figure 4—source data 1. [file elife-95815-fig4-data1.zip › Figure 4—Source Data 1/Figure 4D Raw WB data/Figure 4D K562 N-FOG1.tif]

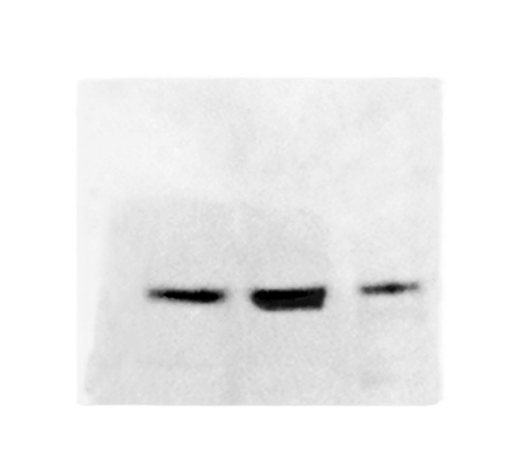

Supplement: Figure 4—source data 1. [file elife-95815-fig4-data1.zip › Figure 4—Source Data 1/Figure 4D Raw WB data/Figure 4D K562 TACC3.tif]

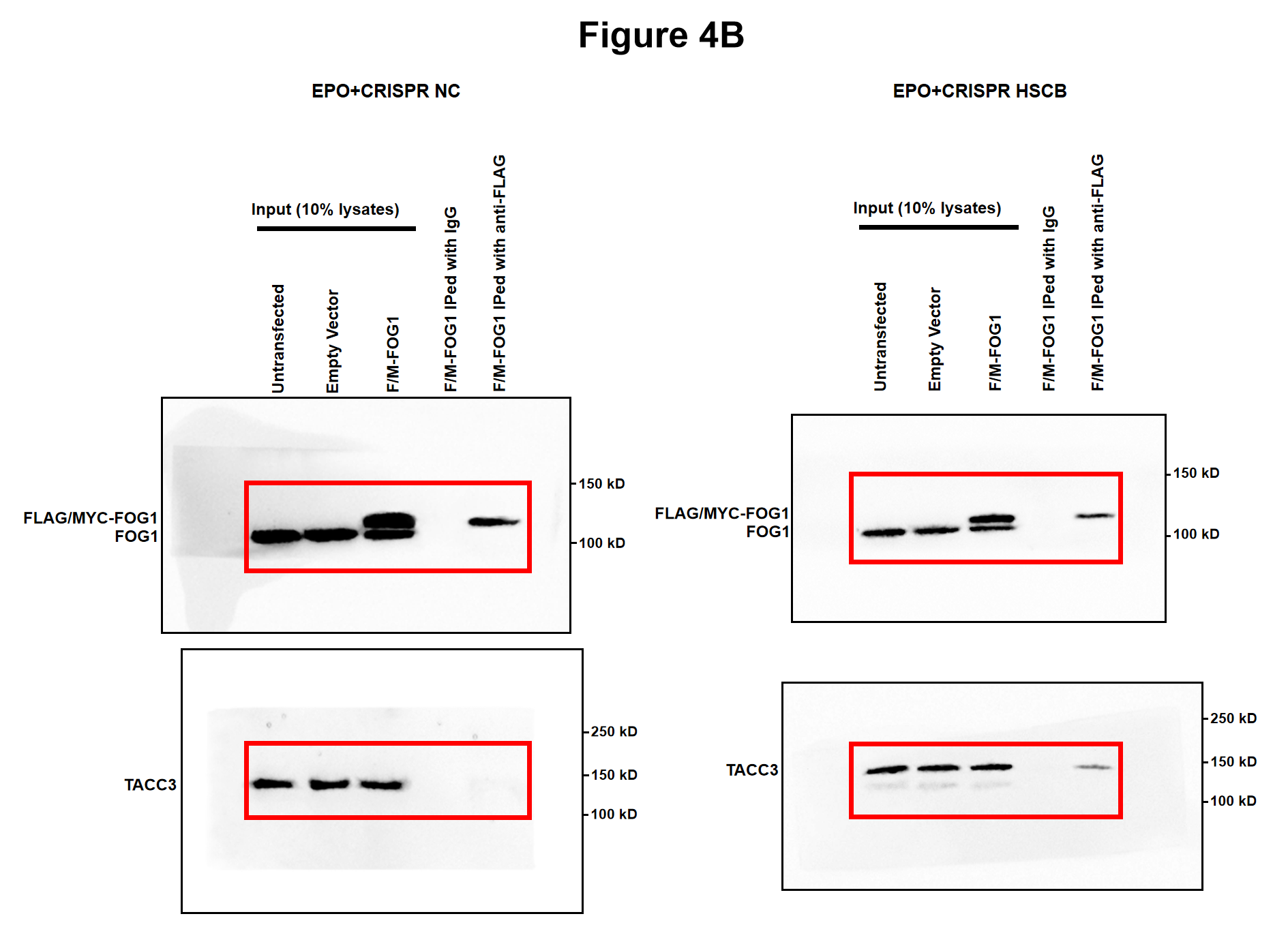

Supplement: Figure 4—source data 1. [file elife-95815-fig4-data1.zip › Figure 4—Source Data 1/Labelled WB data/Source blot data for Figure 4B.tif]

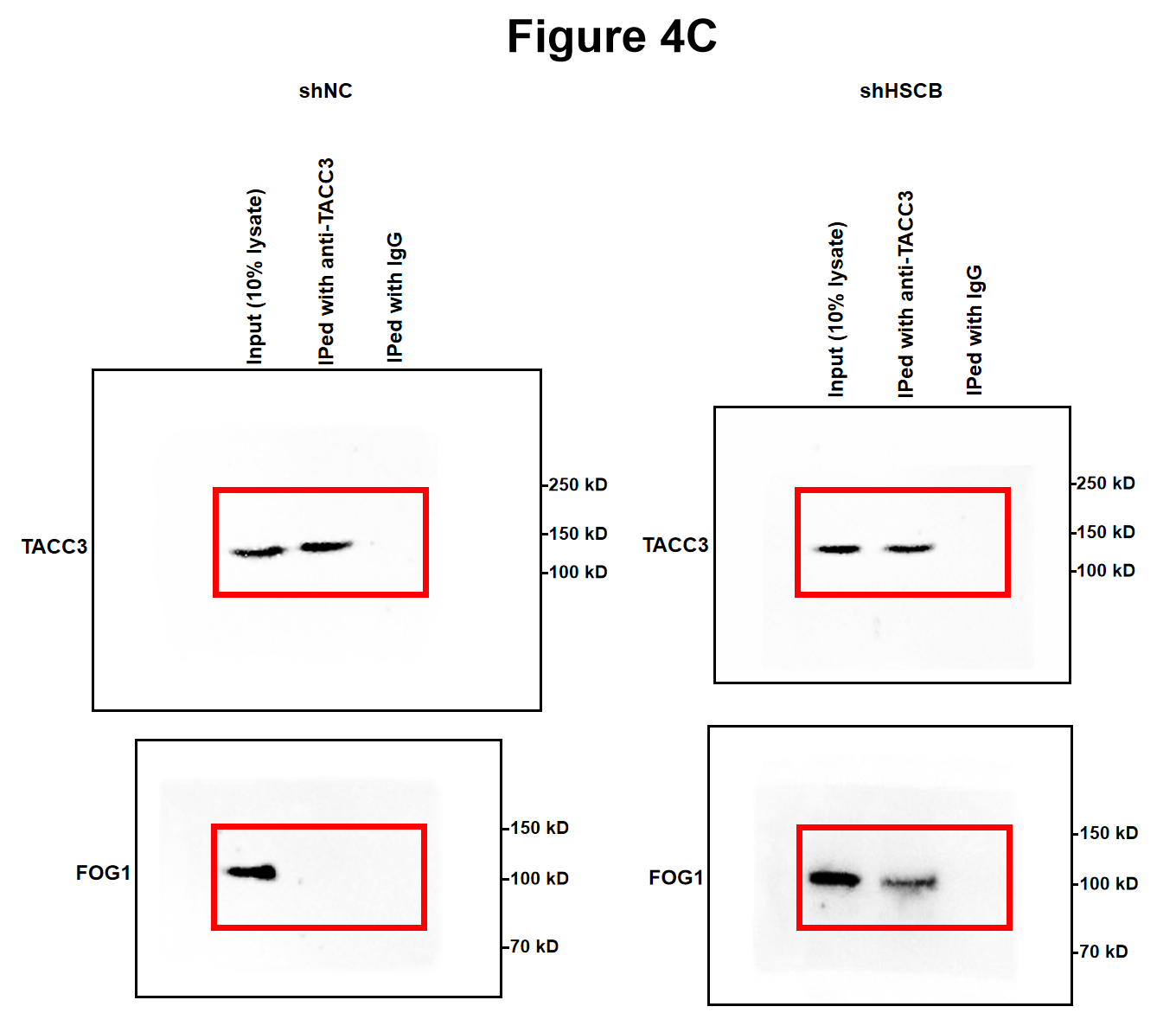

Supplement: Figure 4—source data 1. [file elife-95815-fig4-data1.zip › Figure 4—Source Data 1/Labelled WB data/Source blot data for Figure 4C.tif]

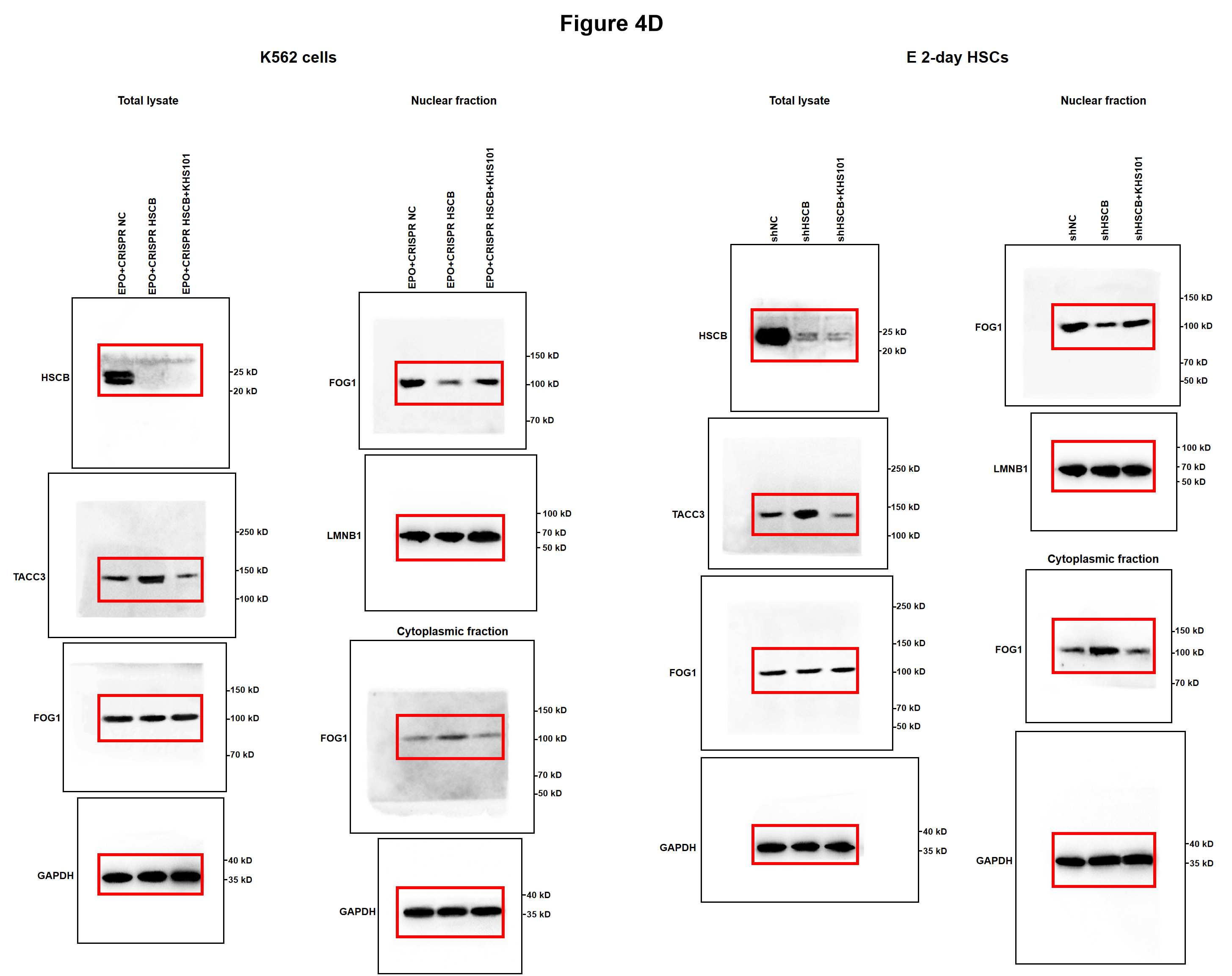

Supplement: Figure 4—source data 1. [file elife-95815-fig4-data1.zip › Figure 4—Source Data 1/Labelled WB data/Source blot data for Figure 4D.tif]

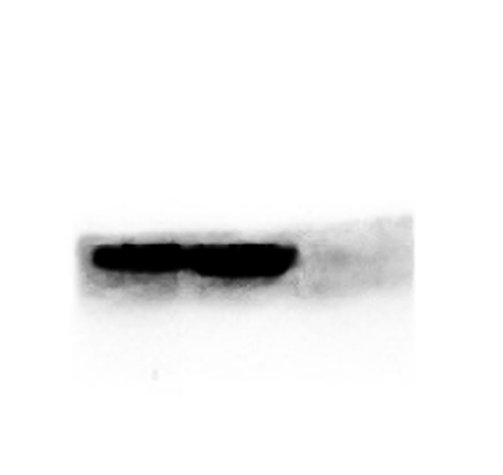

Supplement: Figure 4—figure supplement 1—source data 1. [file elife-95815-fig4-figsupp1-data1.zip › Figure 4–Figure Supplement 1–Source Data 1/Figure 4—figure supplement 1 Raw WB data/Figure 4—figure supplement 1 shHSCB FOG1.tif]

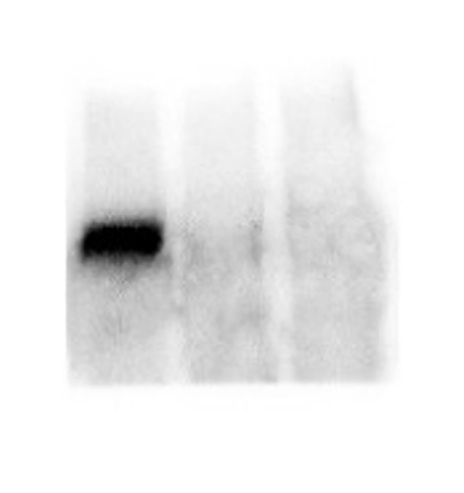

Supplement: Figure 4—figure supplement 1—source data 1. [file elife-95815-fig4-figsupp1-data1.zip › Figure 4–Figure Supplement 1–Source Data 1/Figure 4—figure supplement 1 Raw WB data/Figure 4—figure supplement 1 shHSCB MYO1E.tif]

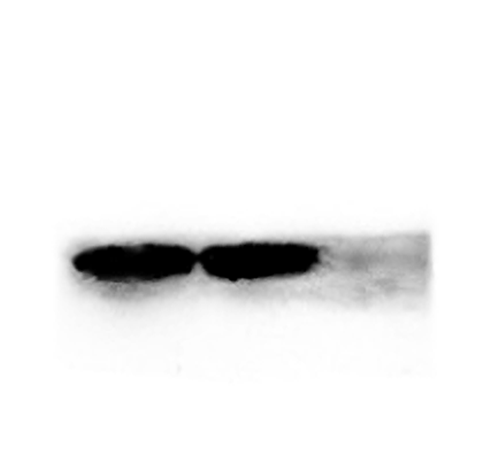

Supplement: Figure 4—figure supplement 1—source data 1. [file elife-95815-fig4-figsupp1-data1.zip › Figure 4–Figure Supplement 1–Source Data 1/Figure 4—figure supplement 1 Raw WB data/Figure 4—figure supplement 1 shNC FOG1.tif]

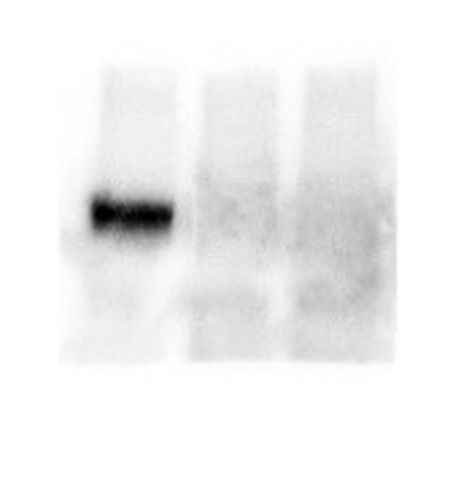

Supplement: Figure 4—figure supplement 1—source data 1. [file elife-95815-fig4-figsupp1-data1.zip › Figure 4–Figure Supplement 1–Source Data 1/Figure 4—figure supplement 1 Raw WB data/Figure 4—figure supplement 1 shNC MYO1E.tif]

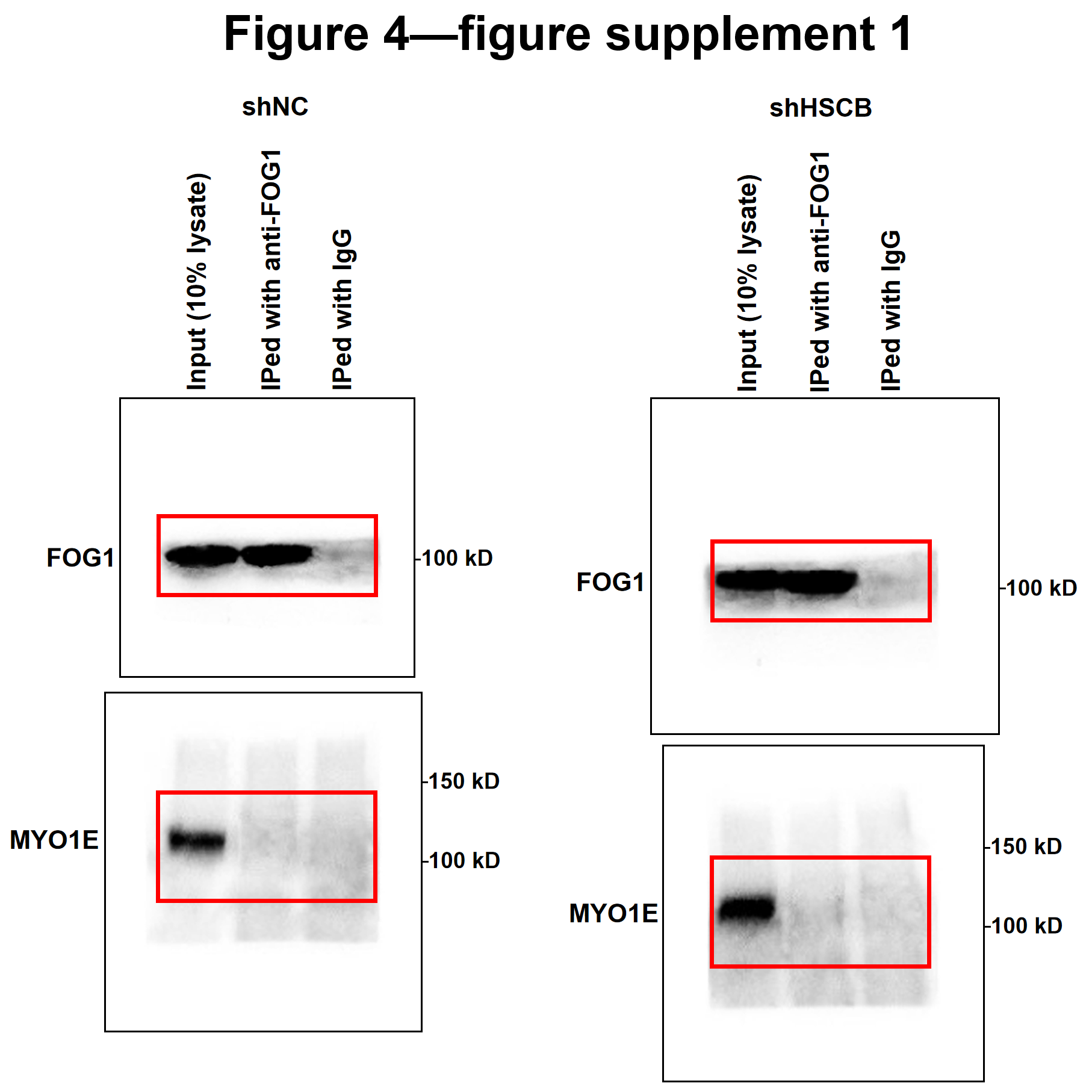

Supplement: Figure 4—figure supplement 1—source data 1. [file elife-95815-fig4-figsupp1-data1.zip › Figure 4–Figure Supplement 1–Source Data 1/Labelled blot data for Figure 4—figure supplement 1.tif]

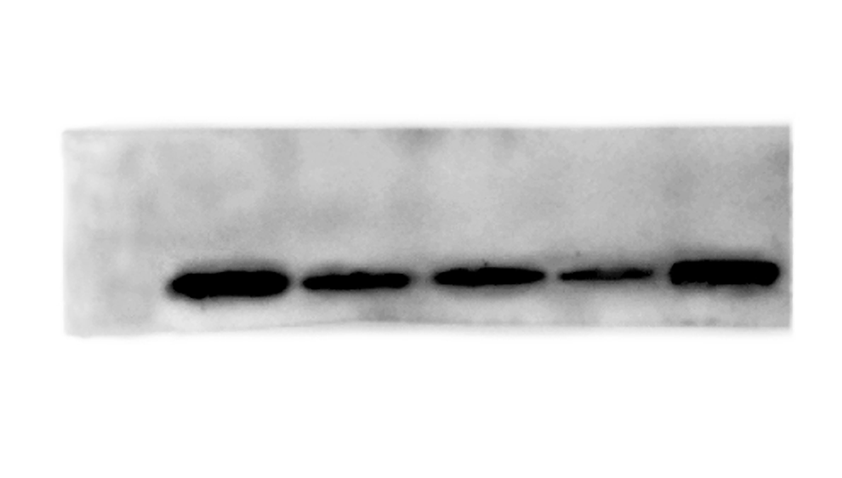

Supplement: Figure 5—source data 1. [file elife-95815-fig5-data1.zip › Figure 5—Source Data 1/Figure 5A Raw WB data/Figure 5A C-FOG1.tif]

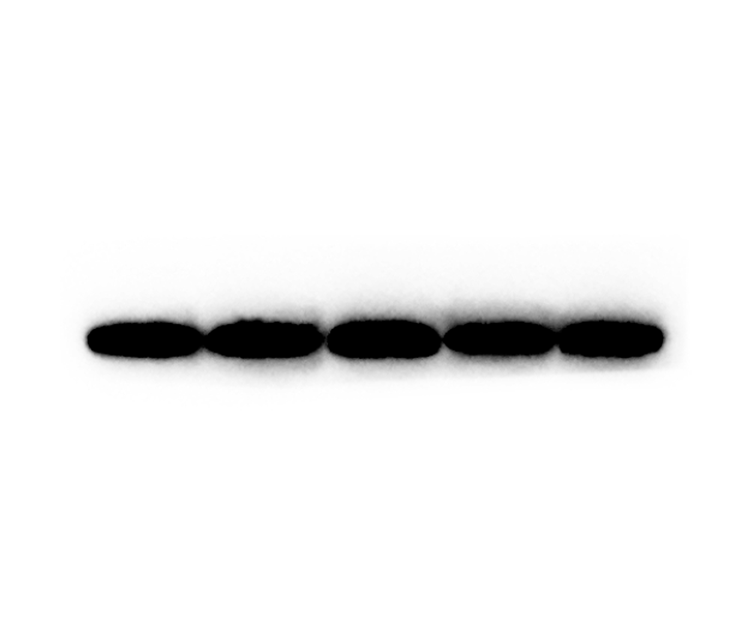

Supplement: Figure 5—source data 1. [file elife-95815-fig5-data1.zip › Figure 5—Source Data 1/Figure 5A Raw WB data/Figure 5A C-GAPDH.tif]

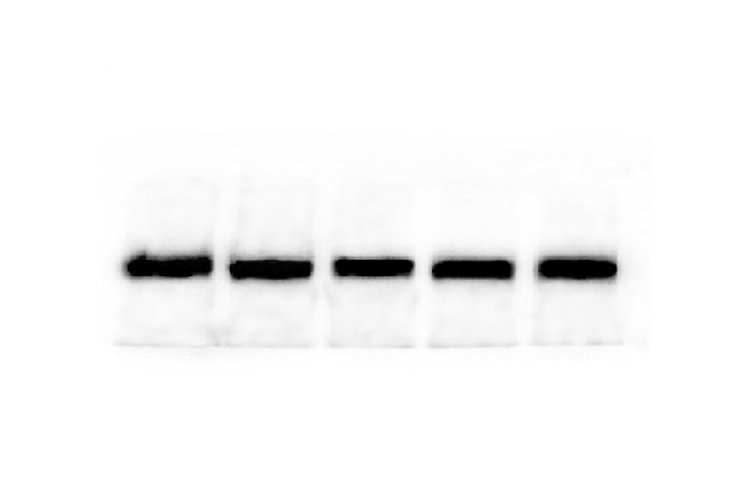

Supplement: Figure 5—source data 1. [file elife-95815-fig5-data1.zip › Figure 5—Source Data 1/Figure 5A Raw WB data/Figure 5A FOG1.tif]

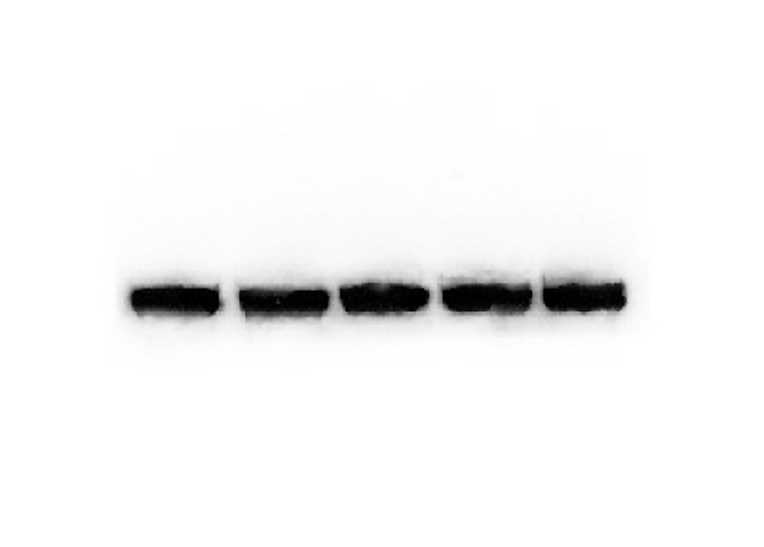

Supplement: Figure 5—source data 1. [file elife-95815-fig5-data1.zip › Figure 5—Source Data 1/Figure 5A Raw WB data/Figure 5A GAPDH.tif]

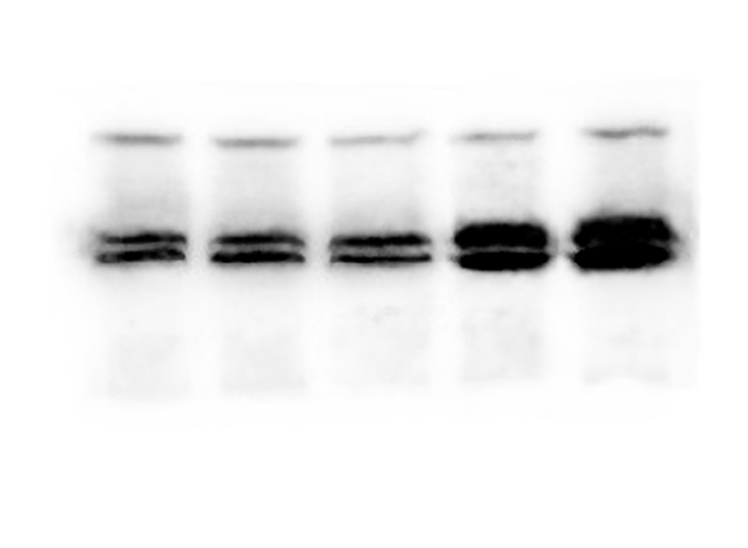

Supplement: Figure 5—source data 1. [file elife-95815-fig5-data1.zip › Figure 5—Source Data 1/Figure 5A Raw WB data/Figure 5A HSCB.tif]

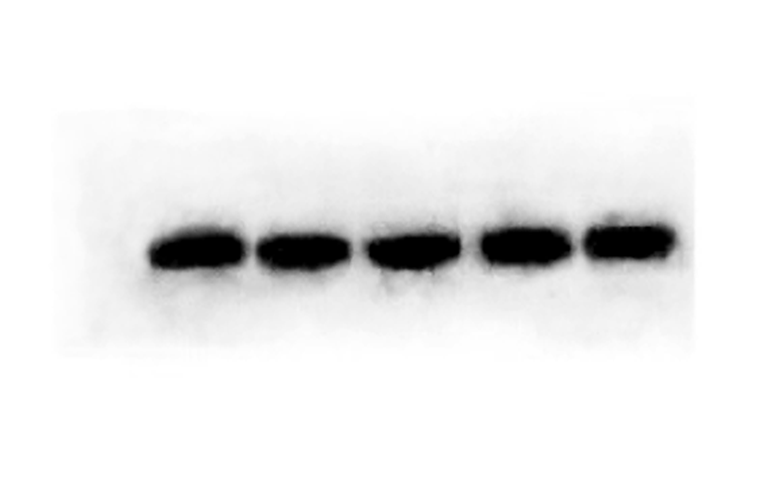

Supplement: Figure 5—source data 1. [file elife-95815-fig5-data1.zip › Figure 5—Source Data 1/Figure 5A Raw WB data/Figure 5A LMNB1.tif]

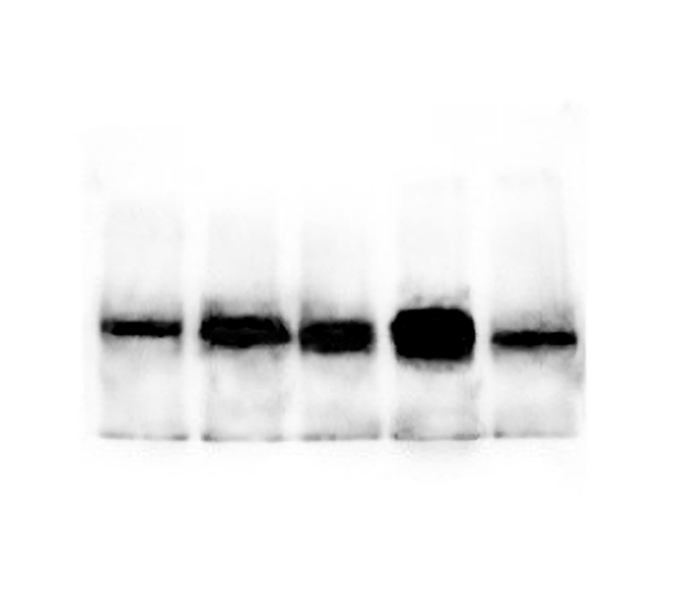

Supplement: Figure 5—source data 1. [file elife-95815-fig5-data1.zip › Figure 5—Source Data 1/Figure 5A Raw WB data/Figure 5A N-FOG1.tif]

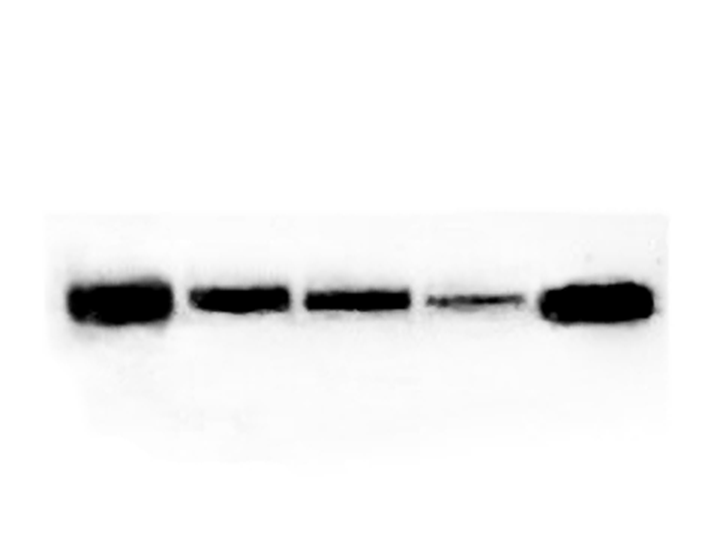

Supplement: Figure 5—source data 1. [file elife-95815-fig5-data1.zip › Figure 5—Source Data 1/Figure 5A Raw WB data/Figure 5A TACC3.tif]

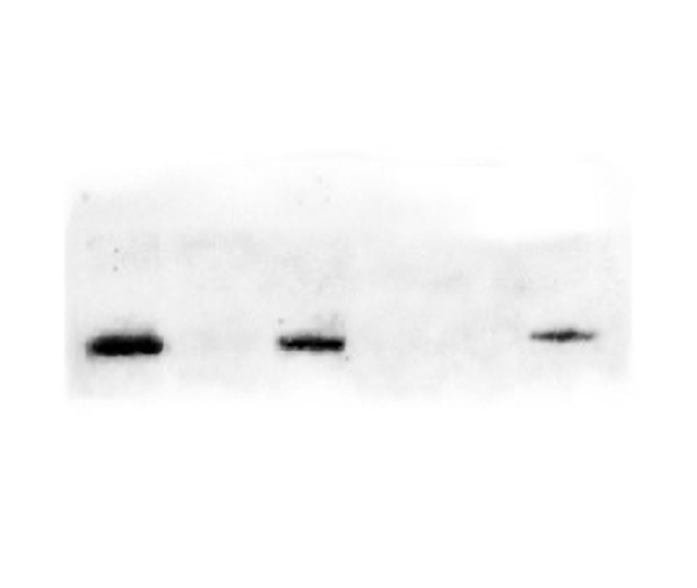

Supplement: Figure 5—source data 1. [file elife-95815-fig5-data1.zip › Figure 5—Source Data 1/Figure 5B Raw WB data/Figure 5B FOG1.tif]

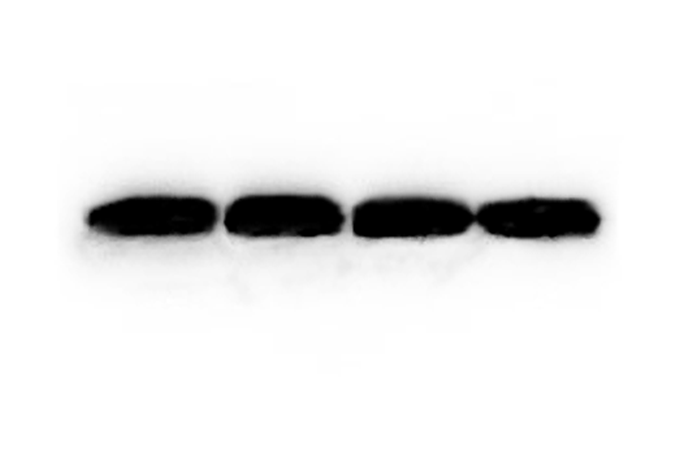

Supplement: Figure 5—source data 1. [file elife-95815-fig5-data1.zip › Figure 5—Source Data 1/Figure 5B Raw WB data/Figure 5B GAPDH.tif]

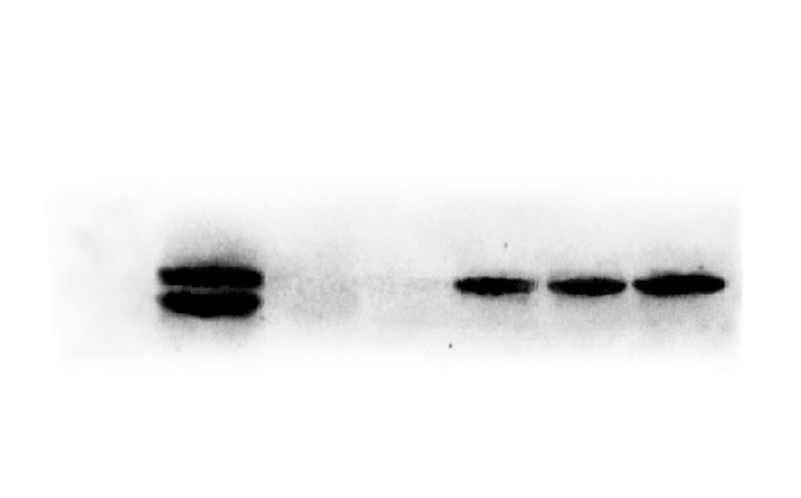

Supplement: Figure 5—source data 1. [file elife-95815-fig5-data1.zip › Figure 5—Source Data 1/Figure 5B Raw WB data/Figure 5B HSCB.tif]

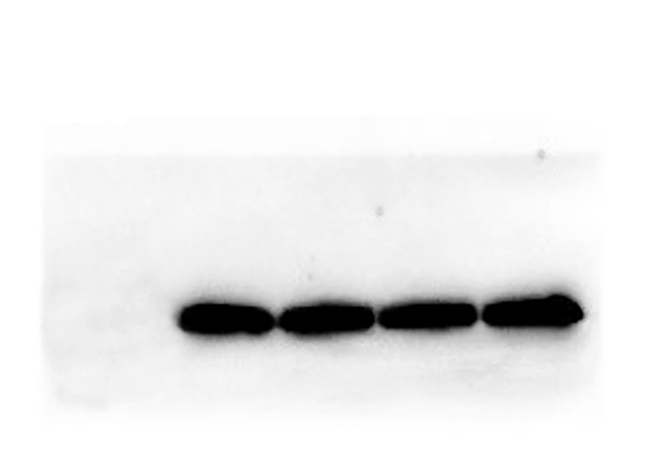

Supplement: Figure 5—source data 1. [file elife-95815-fig5-data1.zip › Figure 5—Source Data 1/Figure 5B Raw WB data/Figure 5B LMNB1.tif]

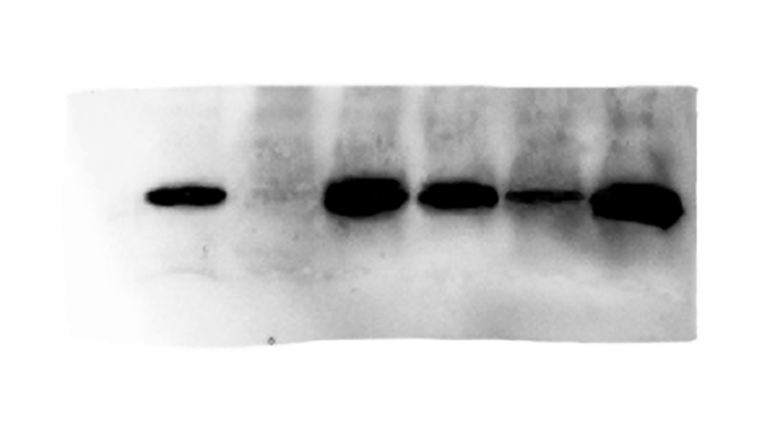

Supplement: Figure 5—source data 1. [file elife-95815-fig5-data1.zip › Figure 5—Source Data 1/Figure 5B Raw WB data/Figure 5B TACC3.tif]

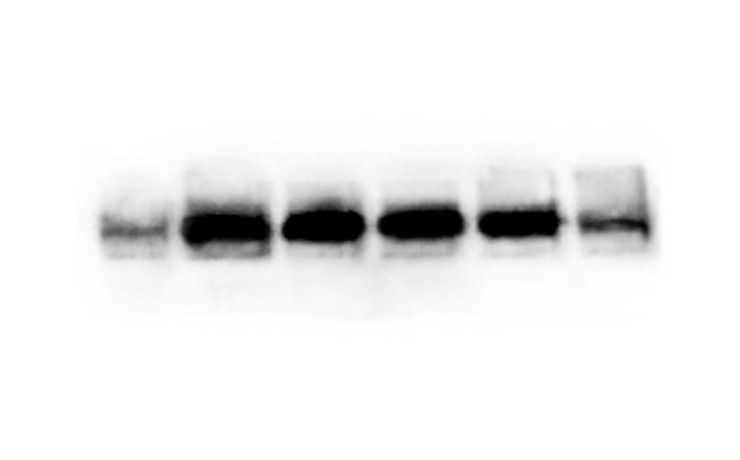

Supplement: Figure 5—source data 1. [file elife-95815-fig5-data1.zip › Figure 5—Source Data 1/Figure 5D Raw WB data/Figure 5D C-FOG1.tif]

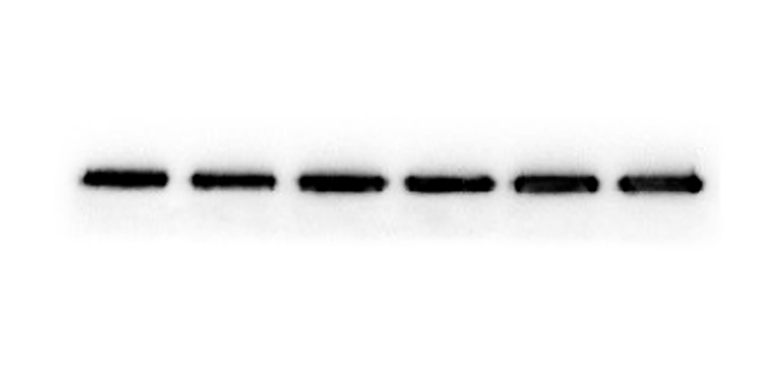

Supplement: Figure 5—source data 1. [file elife-95815-fig5-data1.zip › Figure 5—Source Data 1/Figure 5D Raw WB data/Figure 5D GAPDH.tif]

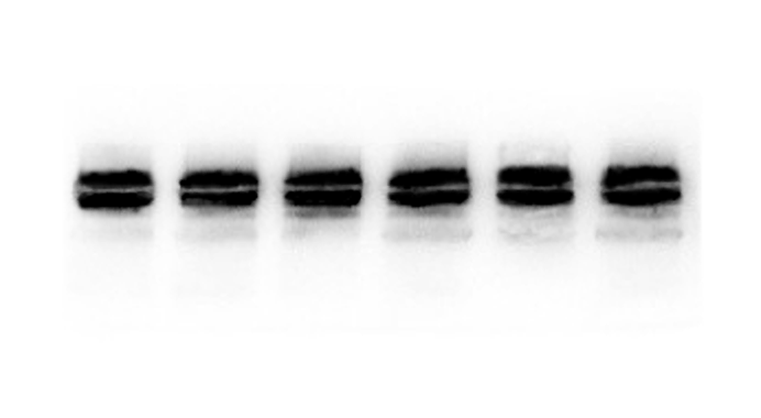

Supplement: Figure 5—source data 1. [file elife-95815-fig5-data1.zip › Figure 5—Source Data 1/Figure 5D Raw WB data/Figure 5D HSCB.tif]

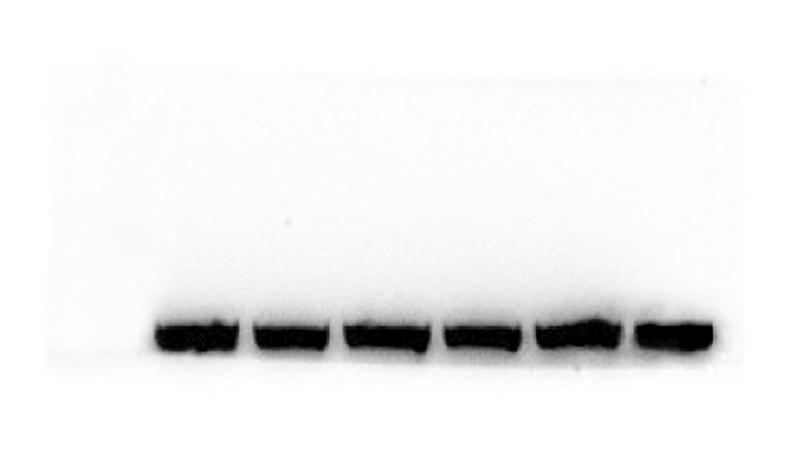

Supplement: Figure 5—source data 1. [file elife-95815-fig5-data1.zip › Figure 5—Source Data 1/Figure 5D Raw WB data/Figure 5D LMNB1.tif]

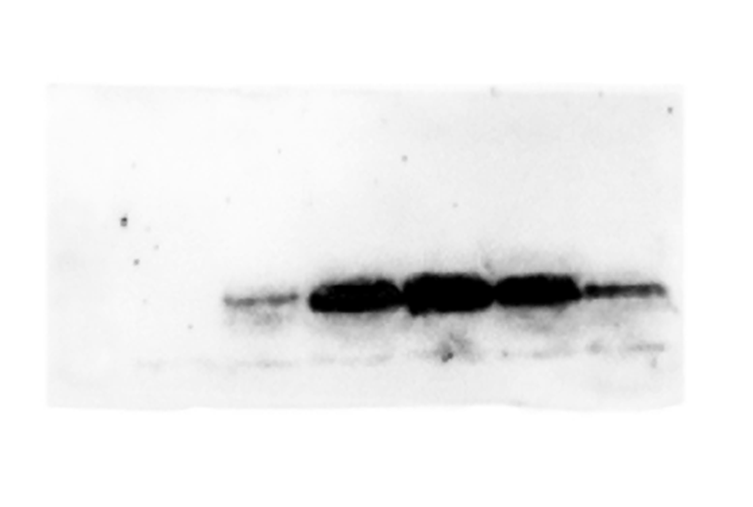

Supplement: Figure 5—source data 1. [file elife-95815-fig5-data1.zip › Figure 5—Source Data 1/Figure 5D Raw WB data/Figure 5D N-FOG1.tif]

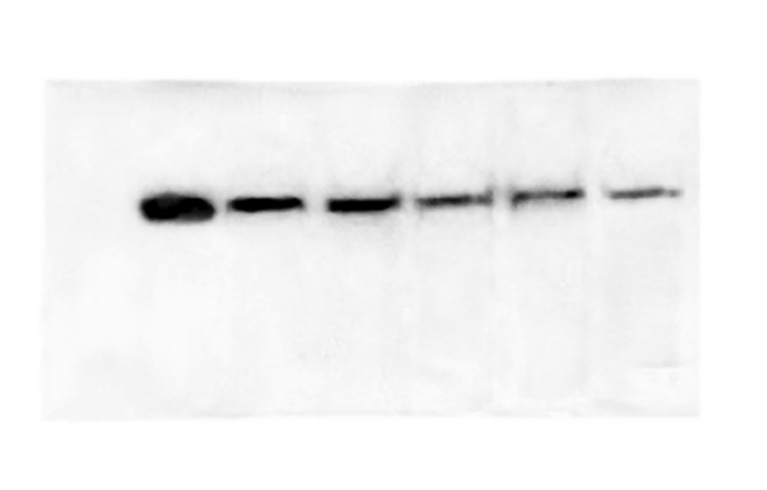

Supplement: Figure 5—source data 1. [file elife-95815-fig5-data1.zip › Figure 5—Source Data 1/Figure 5D Raw WB data/Figure 5D TACC3.tif]

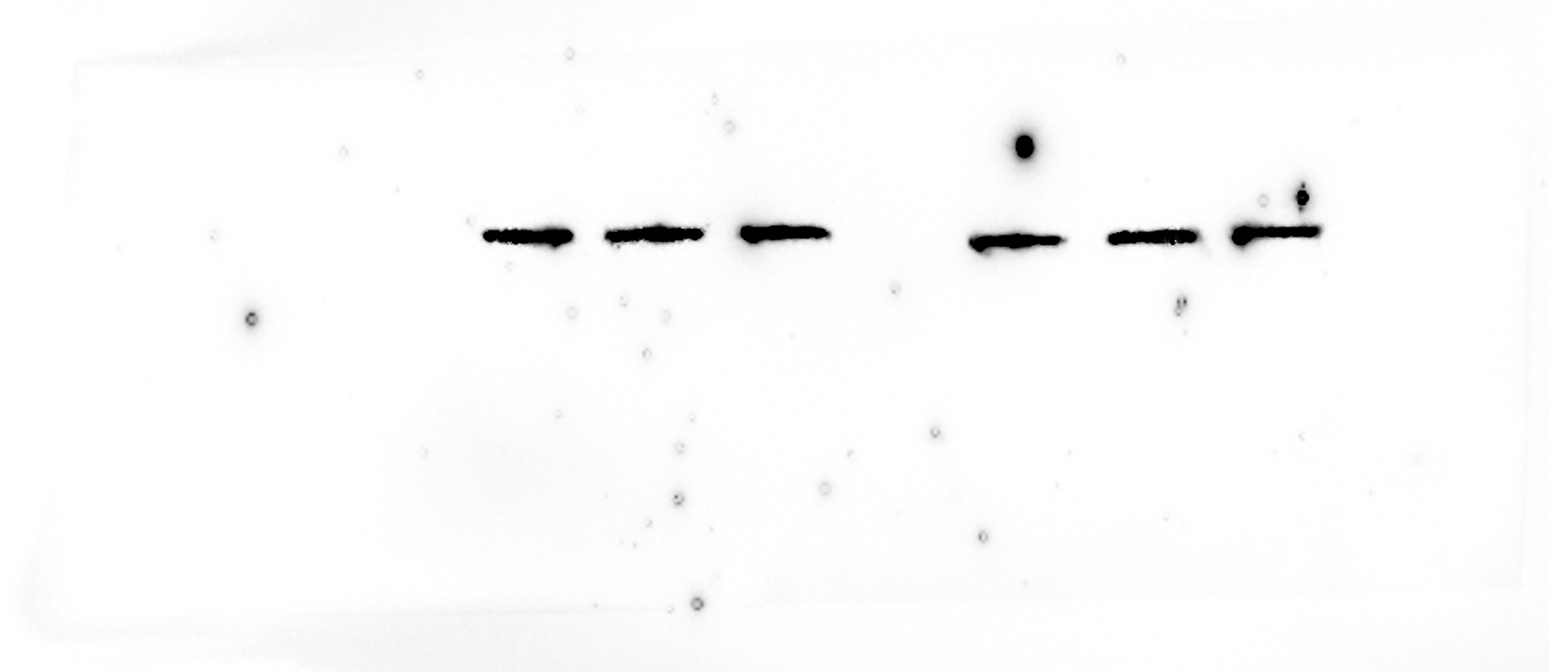

Supplement: Figure 5—source data 1. [file elife-95815-fig5-data1.zip › Figure 5—Source Data 1/Figure 5E Raw WB data/Figure 5E GAPDH.tif]

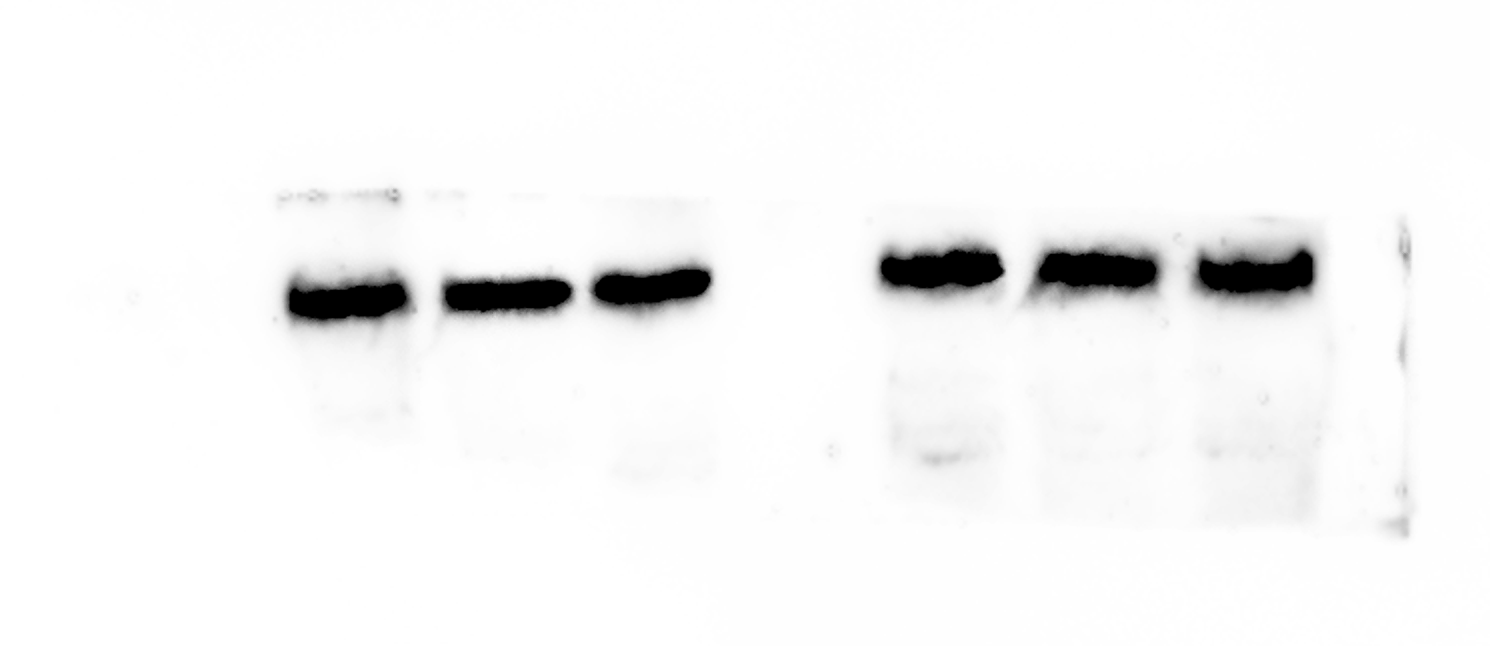

Supplement: Figure 5—source data 1. [file elife-95815-fig5-data1.zip › Figure 5—Source Data 1/Figure 5E Raw WB data/Figure 5E TACC3.tif]

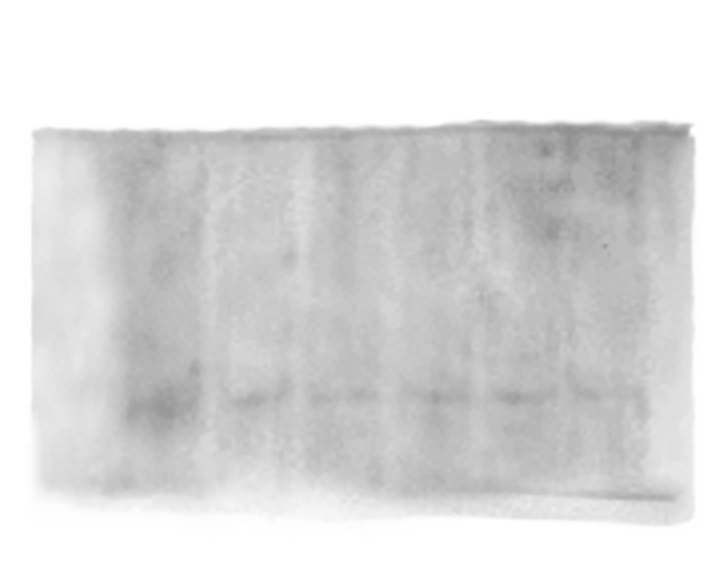

Supplement: Figure 5—source data 1. [file elife-95815-fig5-data1.zip › Figure 5—Source Data 1/Figure 5F Raw WB data/Figure 5F FOG1 IgG.tif]

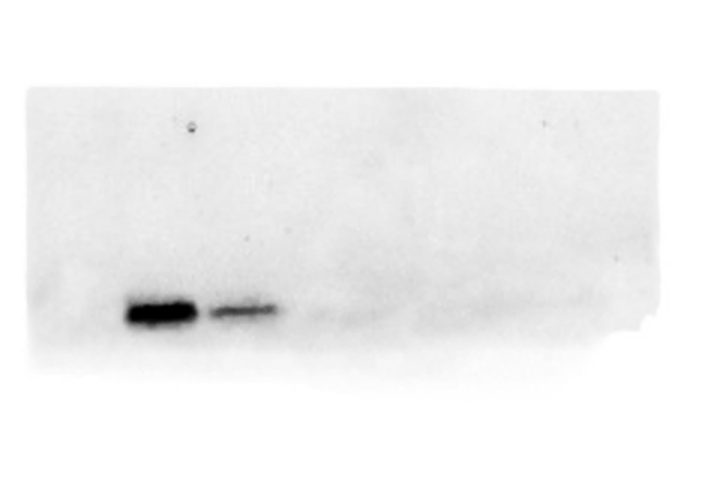

Supplement: Figure 5—source data 1. [file elife-95815-fig5-data1.zip › Figure 5—Source Data 1/Figure 5F Raw WB data/Figure 5F FOG1.tif]

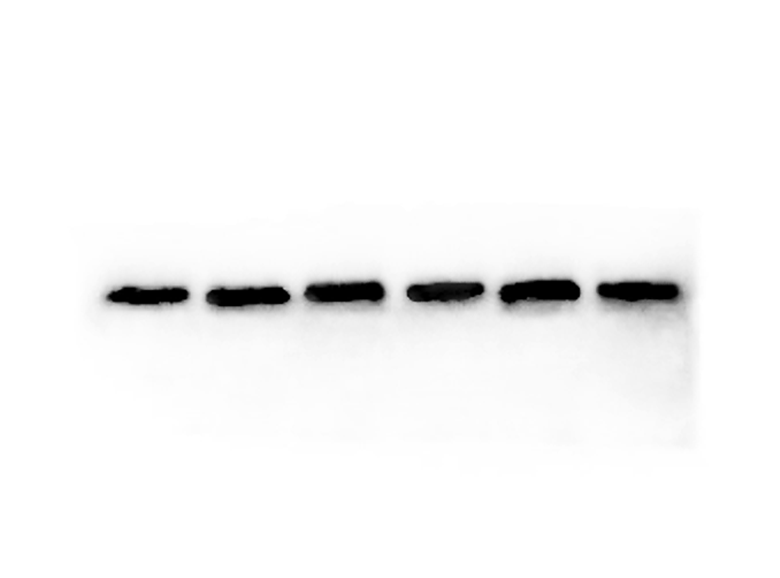

Supplement: Figure 5—source data 1. [file elife-95815-fig5-data1.zip › Figure 5—Source Data 1/Figure 5F Raw WB data/Figure 5F GAPDH.tif]

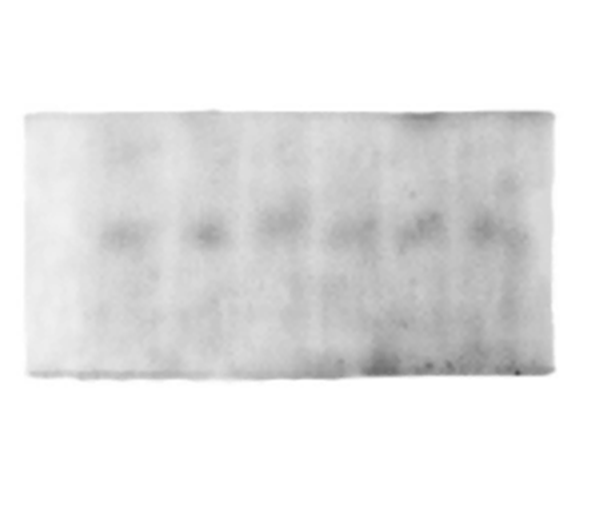

Supplement: Figure 5—source data 1. [file elife-95815-fig5-data1.zip › Figure 5—Source Data 1/Figure 5F Raw WB data/Figure 5F HSCB IgG.tif]

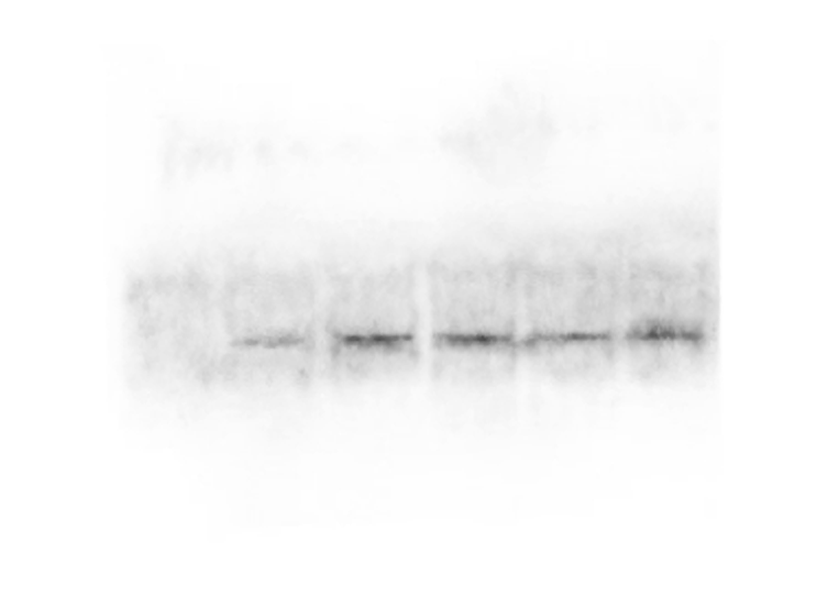

Supplement: Figure 5—source data 1. [file elife-95815-fig5-data1.zip › Figure 5—Source Data 1/Figure 5F Raw WB data/Figure 5F HSCB.tif]

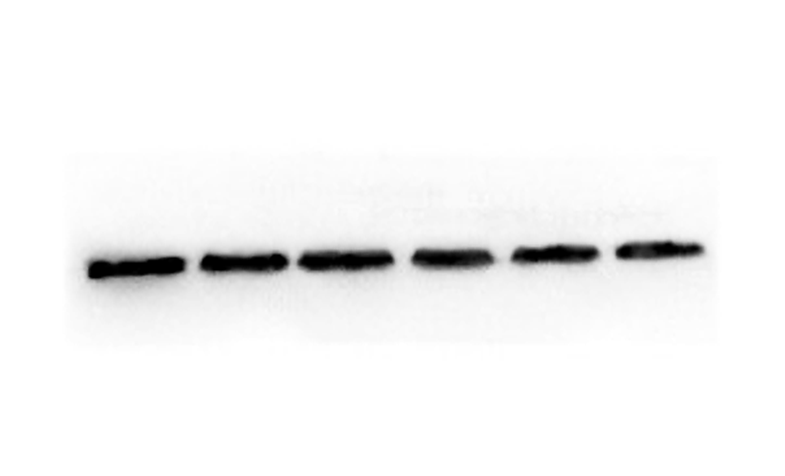

Supplement: Figure 5—source data 1. [file elife-95815-fig5-data1.zip › Figure 5—Source Data 1/Figure 5F Raw WB data/Figure 5F LMNB1.tif]

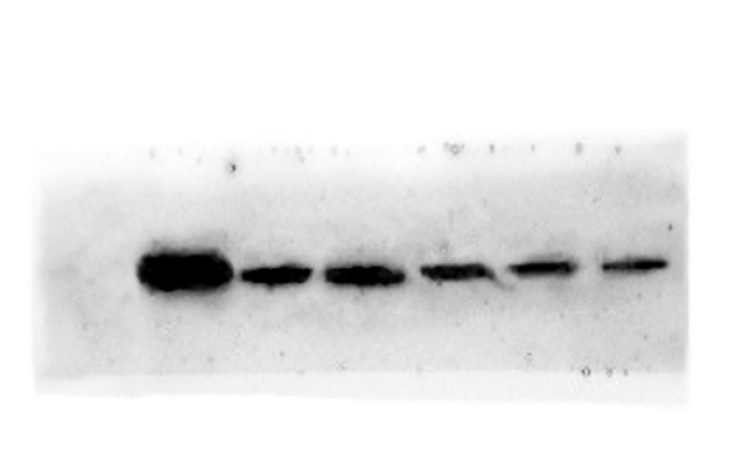

Supplement: Figure 5—source data 1. [file elife-95815-fig5-data1.zip › Figure 5—Source Data 1/Figure 5F Raw WB data/Figure 5F TACC3.tif]
