# Supplementary figures and images for: PI3K/HSCB axis facilitates FOG1 nuclear translocation to promote erythropoiesis and megakaryopoiesis (part 2 of 2)
Source: eLife. 2024 May 17;13:RP95815. doi: 10.7554/eLife.95815 (PMC11101173; doi:10.7554/eLife.95815)

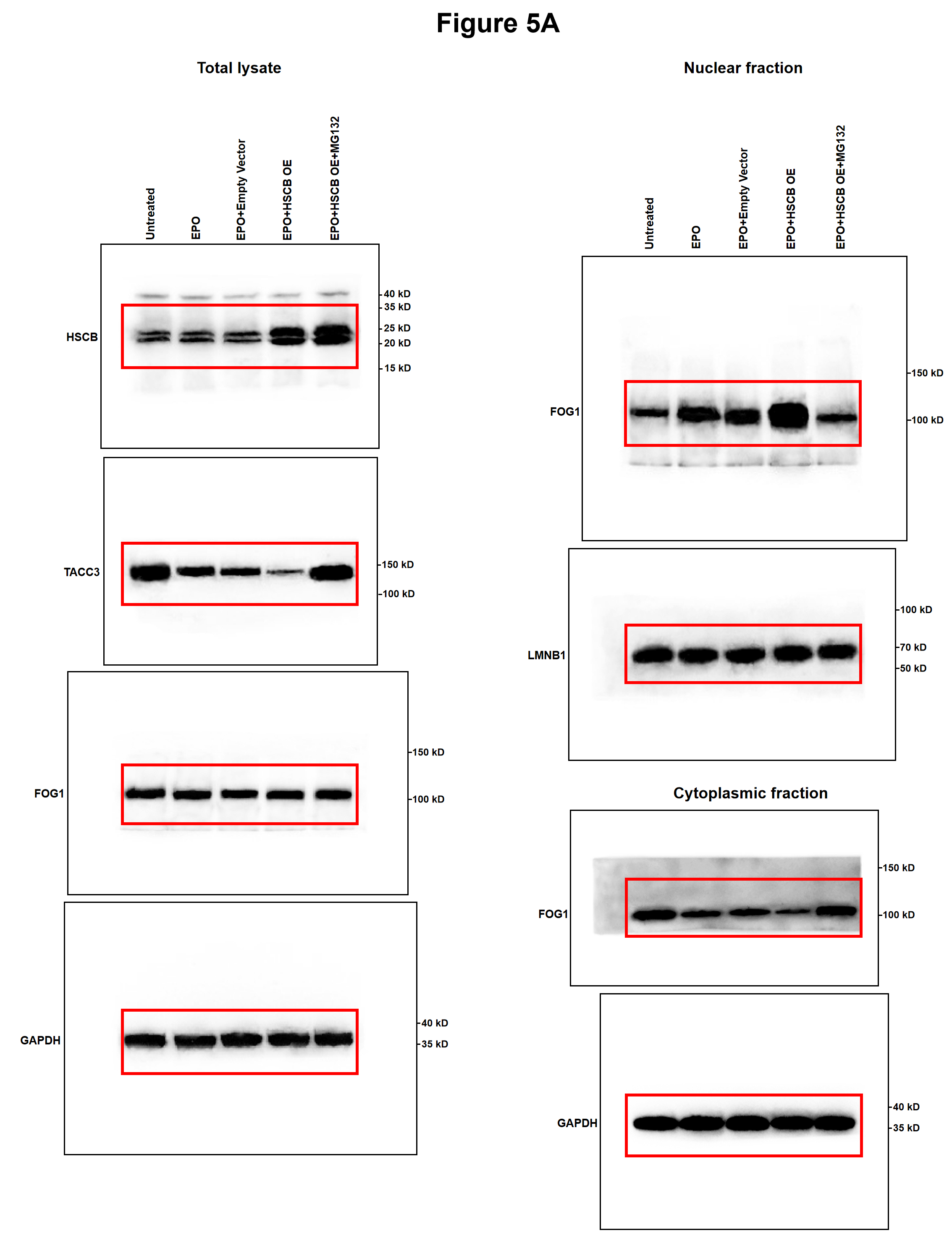

Supplement: Figure 5—source data 1. [file elife-95815-fig5-data1.zip › Figure 5—Source Data 1/Labelled WB data/Source blot data for Figure 5A.tif]

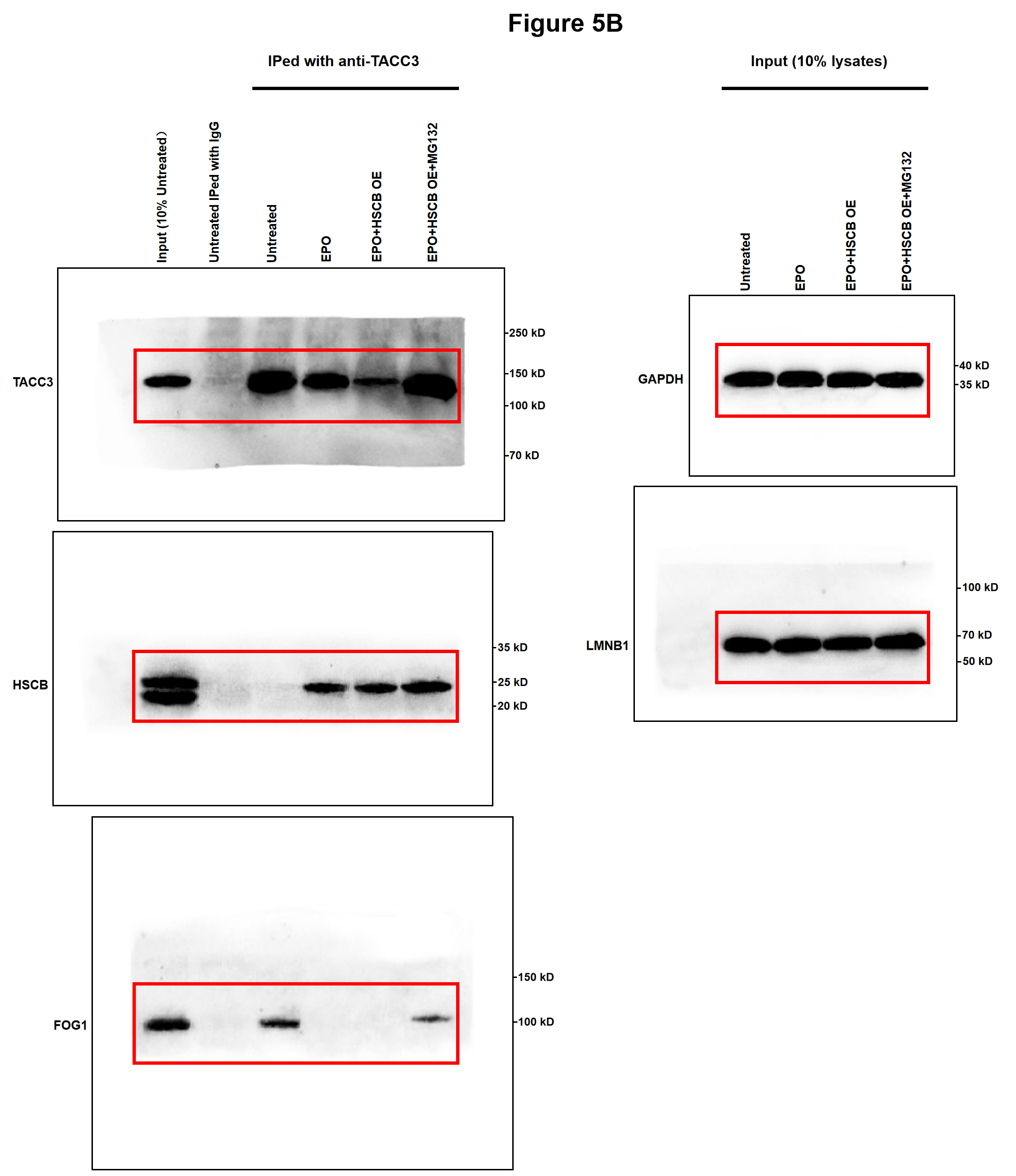

Supplement: Figure 5—source data 1. [file elife-95815-fig5-data1.zip › Figure 5—Source Data 1/Labelled WB data/Source blot data for Figure 5B.tif]

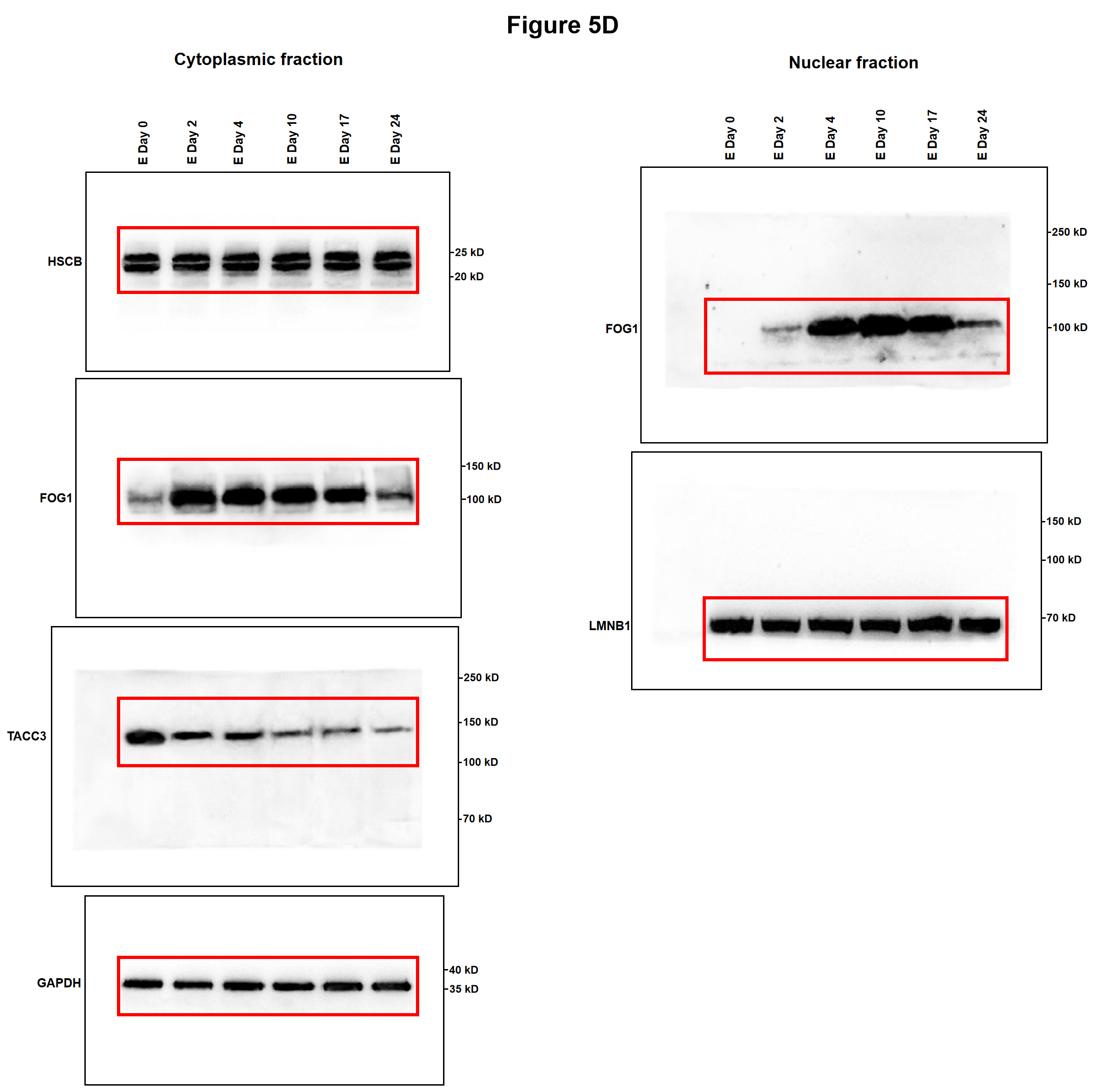

Supplement: Figure 5—source data 1. [file elife-95815-fig5-data1.zip › Figure 5—Source Data 1/Labelled WB data/Source blot data for Figure 5D.tif]

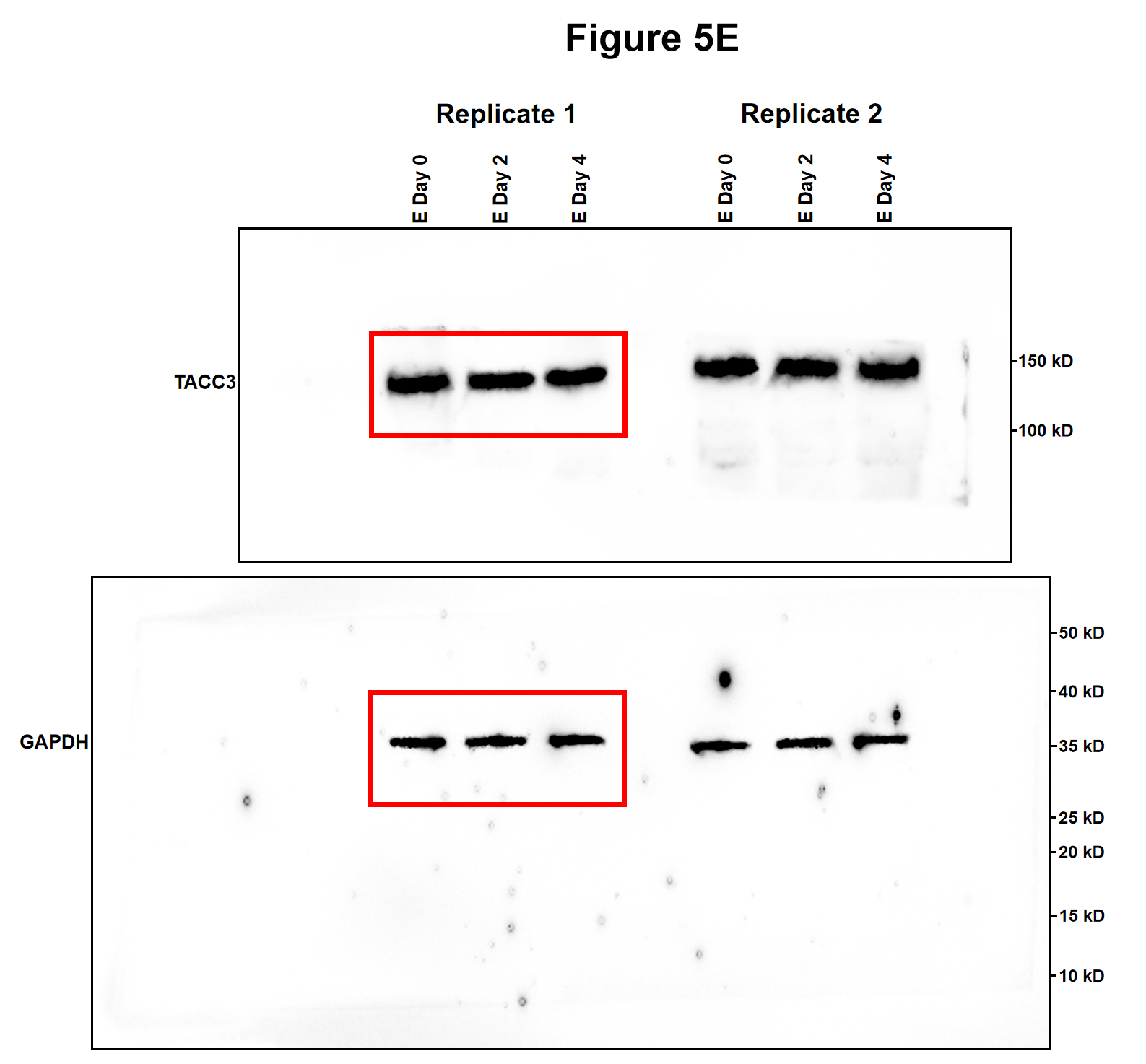

Supplement: Figure 5—source data 1. [file elife-95815-fig5-data1.zip › Figure 5—Source Data 1/Labelled WB data/Source blot data for Figure 5E.tif]

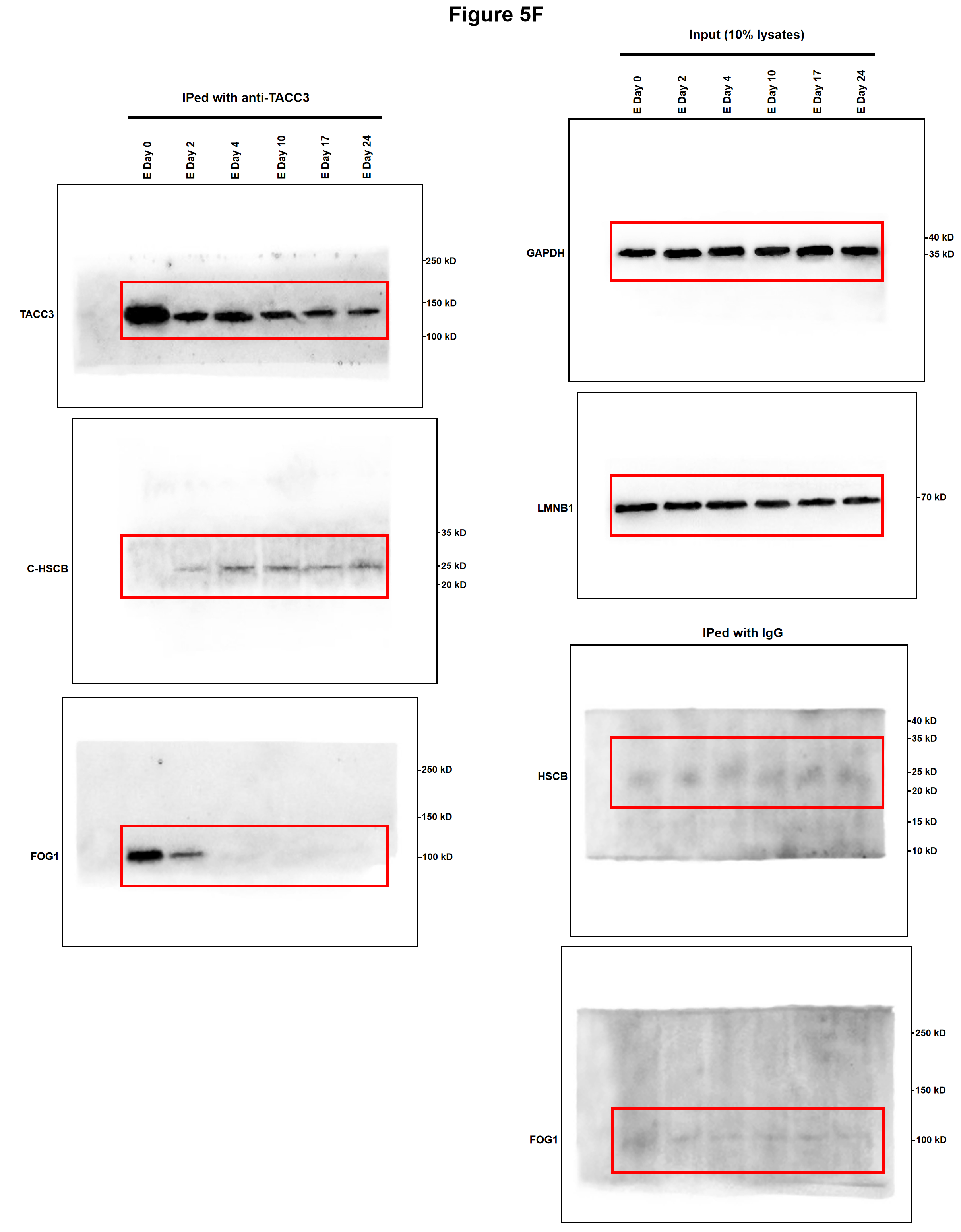

Supplement: Figure 5—source data 1. [file elife-95815-fig5-data1.zip › Figure 5—Source Data 1/Labelled WB data/Source blot data for Figure 5F.tif]

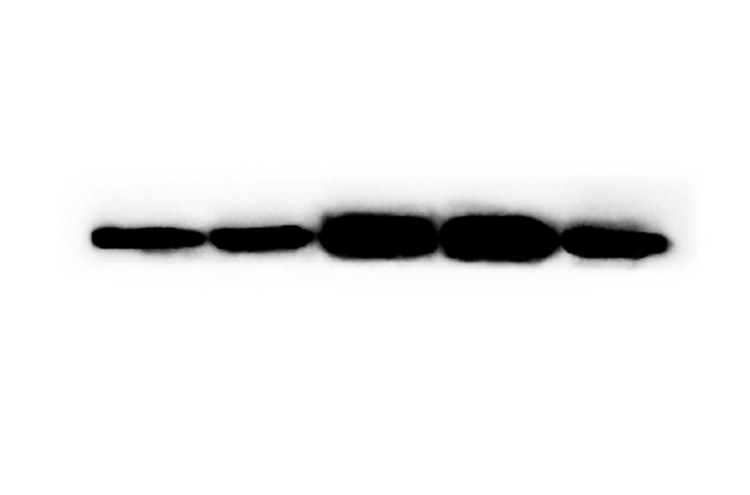

Supplement: Figure 6—source data 1. [file elife-95815-fig6-data1.zip › Figure 6—Source Data 1/Figure 6A Raw WB data/Figure 6A C-FOG1.tif]

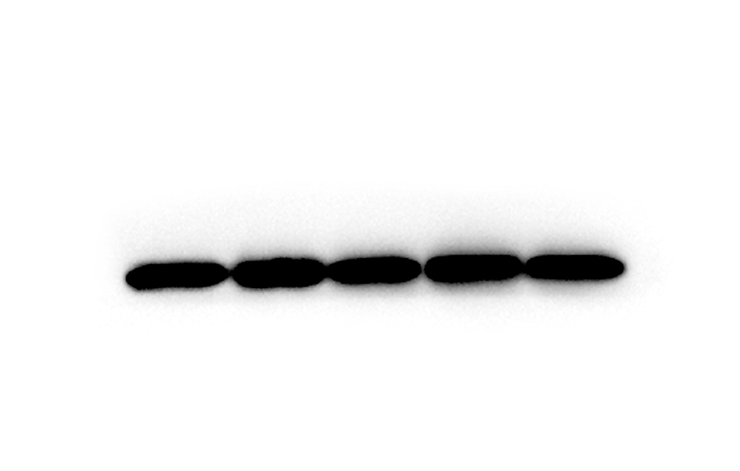

Supplement: Figure 6—source data 1. [file elife-95815-fig6-data1.zip › Figure 6—Source Data 1/Figure 6A Raw WB data/Figure 6A C-GAPDH.tif]

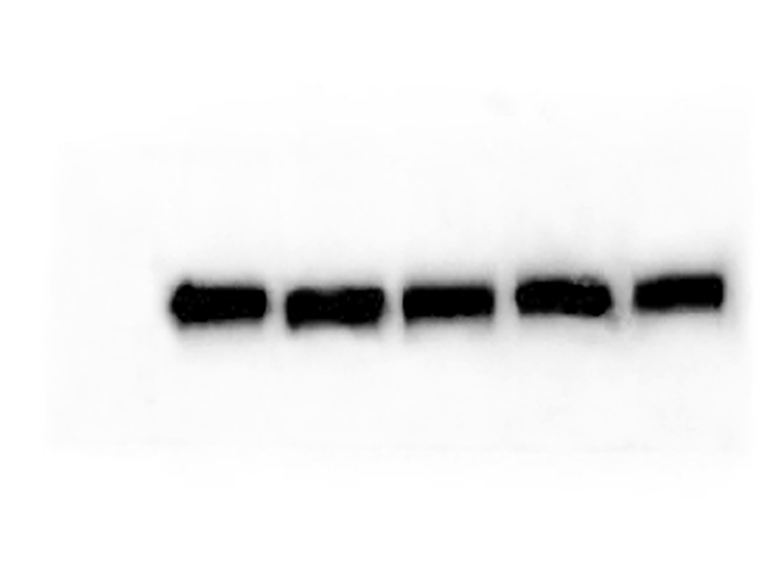

Supplement: Figure 6—source data 1. [file elife-95815-fig6-data1.zip › Figure 6—Source Data 1/Figure 6A Raw WB data/Figure 6A FOG1.tif]

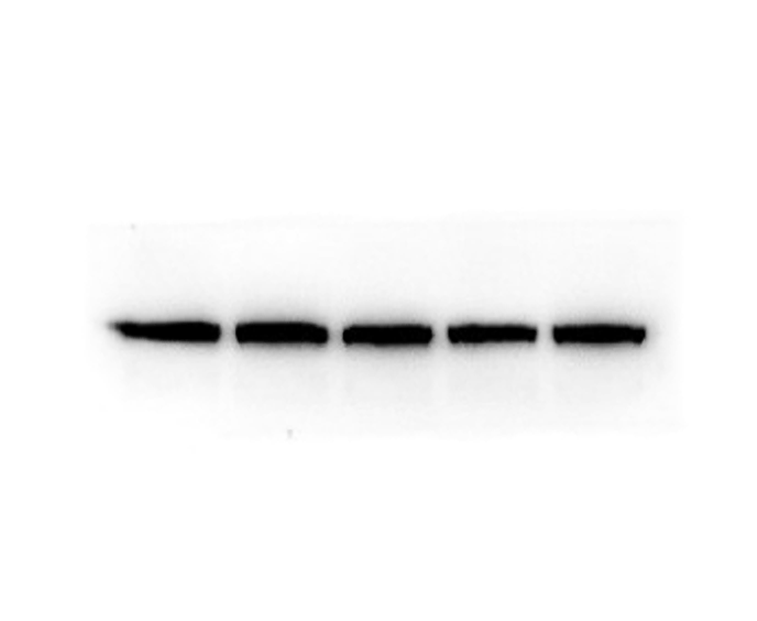

Supplement: Figure 6—source data 1. [file elife-95815-fig6-data1.zip › Figure 6—Source Data 1/Figure 6A Raw WB data/Figure 6A GAPDH.tif]

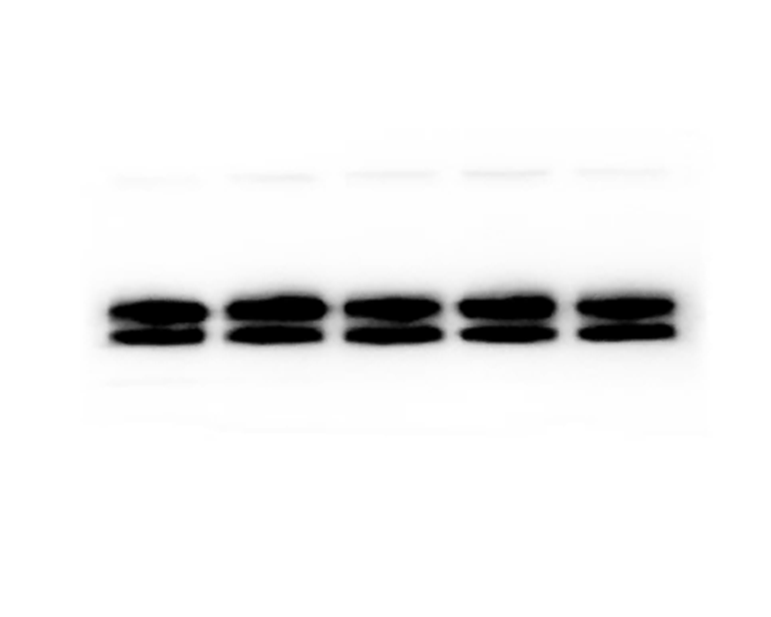

Supplement: Figure 6—source data 1. [file elife-95815-fig6-data1.zip › Figure 6—Source Data 1/Figure 6A Raw WB data/Figure 6A HSCB.tif]

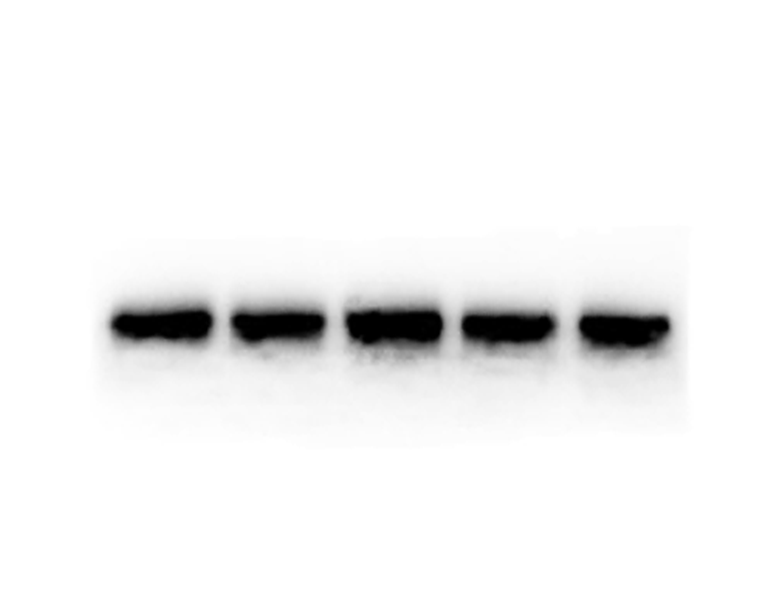

Supplement: Figure 6—source data 1. [file elife-95815-fig6-data1.zip › Figure 6—Source Data 1/Figure 6A Raw WB data/Figure 6A LMNB1.tif]

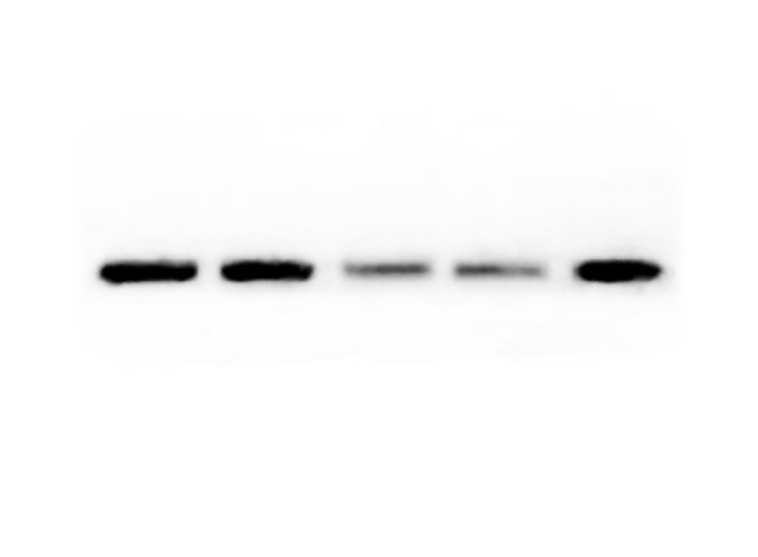

Supplement: Figure 6—source data 1. [file elife-95815-fig6-data1.zip › Figure 6—Source Data 1/Figure 6A Raw WB data/Figure 6A N-FOG1.tif]

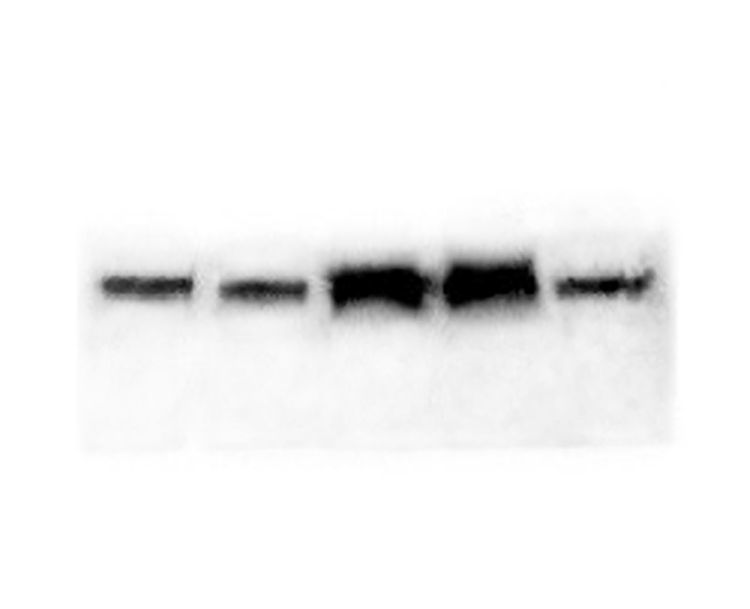

Supplement: Figure 6—source data 1. [file elife-95815-fig6-data1.zip › Figure 6—Source Data 1/Figure 6A Raw WB data/Figure 6A TACC3.tif]

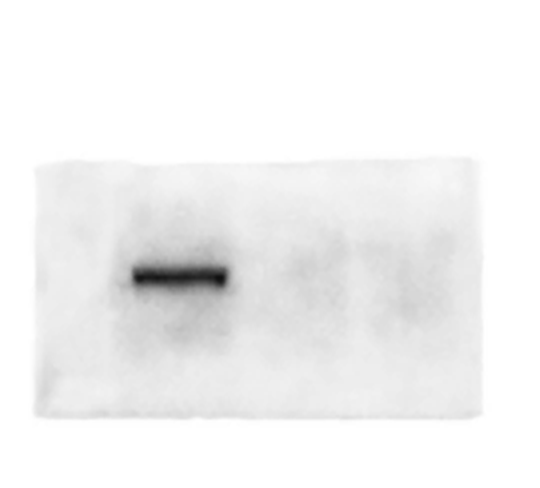

Supplement: Figure 6—source data 1. [file elife-95815-fig6-data1.zip › Figure 6—Source Data 1/Figure 6B Raw WB data/Figure 6B EPO+Vehicle FOG1.tif]

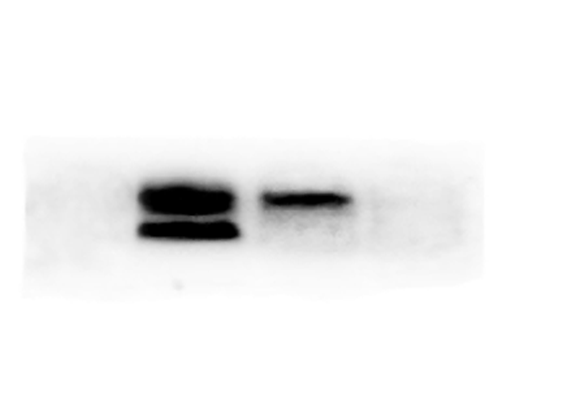

Supplement: Figure 6—source data 1. [file elife-95815-fig6-data1.zip › Figure 6—Source Data 1/Figure 6B Raw WB data/Figure 6B EPO+Vehicle HSCB.tif]

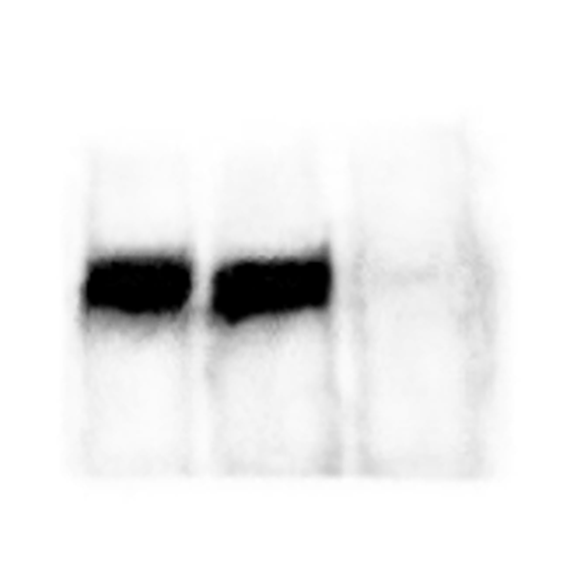

Supplement: Figure 6—source data 1. [file elife-95815-fig6-data1.zip › Figure 6—Source Data 1/Figure 6B Raw WB data/Figure 6B EPO+Vehicle TACC3.tif]

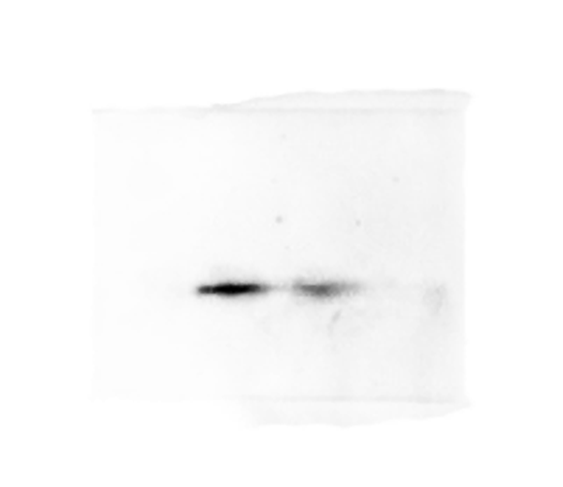

Supplement: Figure 6—source data 1. [file elife-95815-fig6-data1.zip › Figure 6—Source Data 1/Figure 6B Raw WB data/Figure 6B EPO+Wortmannin FOG1.tif]

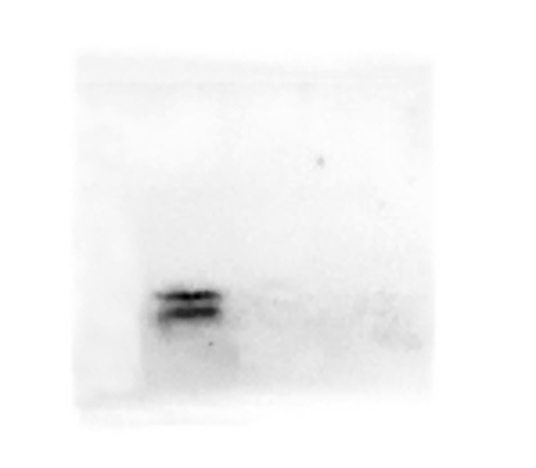

Supplement: Figure 6—source data 1. [file elife-95815-fig6-data1.zip › Figure 6—Source Data 1/Figure 6B Raw WB data/Figure 6B EPO+Wortmannin HSCB.tif]

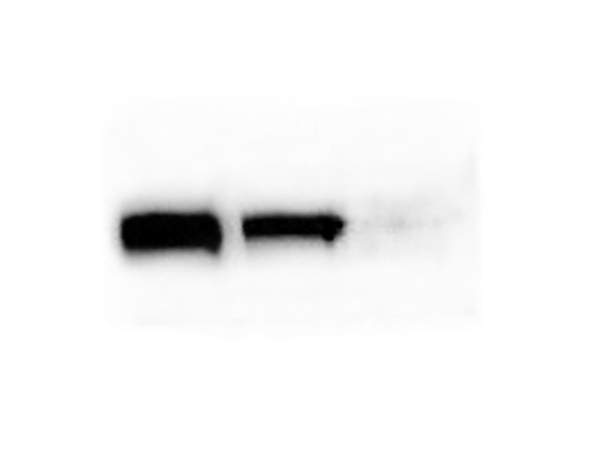

Supplement: Figure 6—source data 1. [file elife-95815-fig6-data1.zip › Figure 6—Source Data 1/Figure 6B Raw WB data/Figure 6B EPO+Wortmannin TACC3.tif]

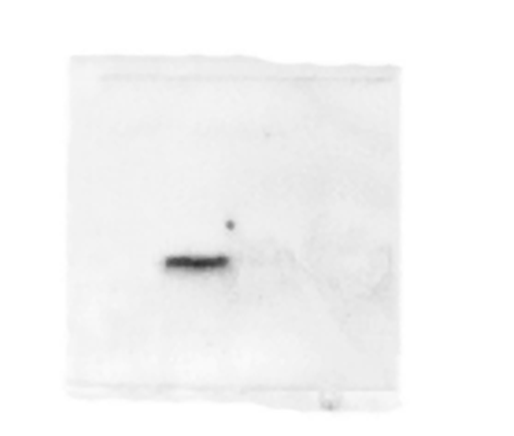

Supplement: Figure 6—source data 1. [file elife-95815-fig6-data1.zip › Figure 6—Source Data 1/Figure 6B Raw WB data/Figure 6B Vehicle FOG1.tif]

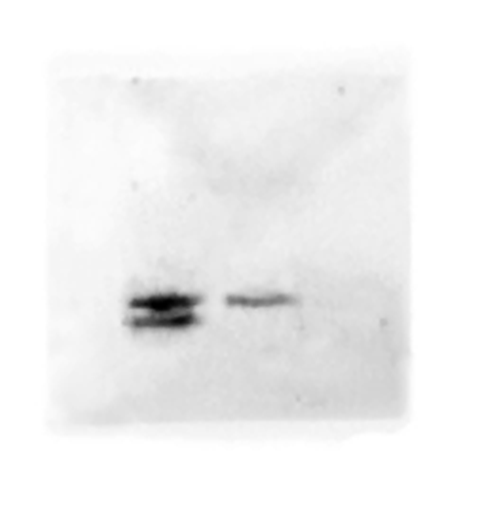

Supplement: Figure 6—source data 1. [file elife-95815-fig6-data1.zip › Figure 6—Source Data 1/Figure 6B Raw WB data/Figure 6B Vehicle HSCB.tif]

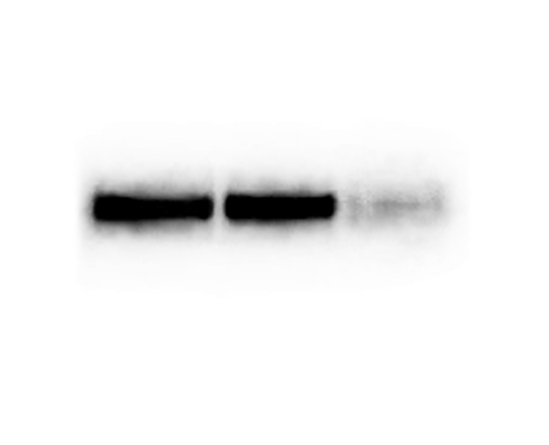

Supplement: Figure 6—source data 1. [file elife-95815-fig6-data1.zip › Figure 6—Source Data 1/Figure 6B Raw WB data/Figure 6B Vehicle TACC3.tif]

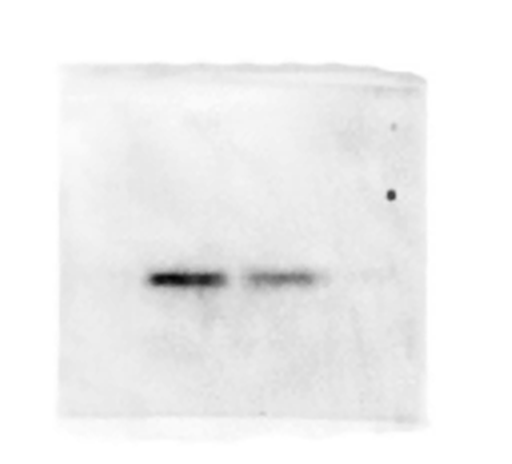

Supplement: Figure 6—source data 1. [file elife-95815-fig6-data1.zip › Figure 6—Source Data 1/Figure 6B Raw WB data/Figure 6B Wortmannin FOG1.tif]

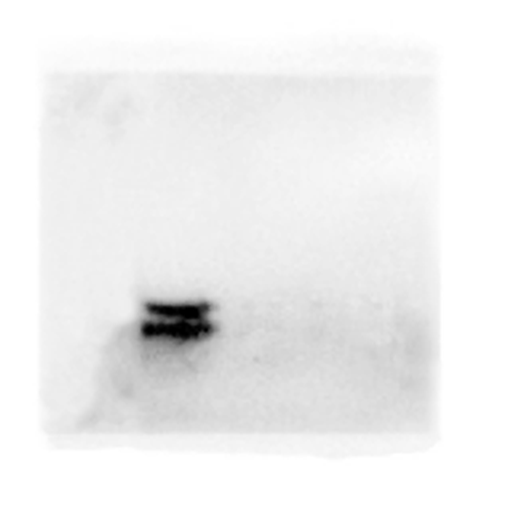

Supplement: Figure 6—source data 1. [file elife-95815-fig6-data1.zip › Figure 6—Source Data 1/Figure 6B Raw WB data/Figure 6B Wortmannin HSCB.tif]

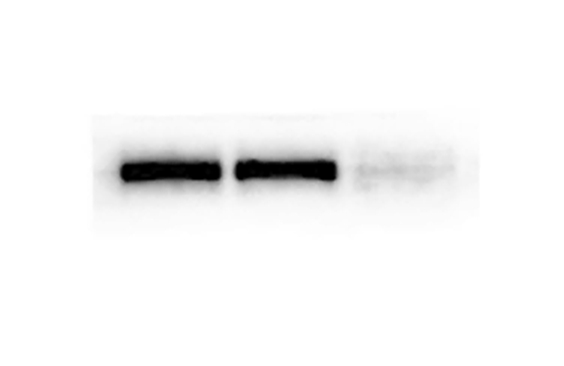

Supplement: Figure 6—source data 1. [file elife-95815-fig6-data1.zip › Figure 6—Source Data 1/Figure 6B Raw WB data/Figure 6B Wortmannin TACC3.tif]

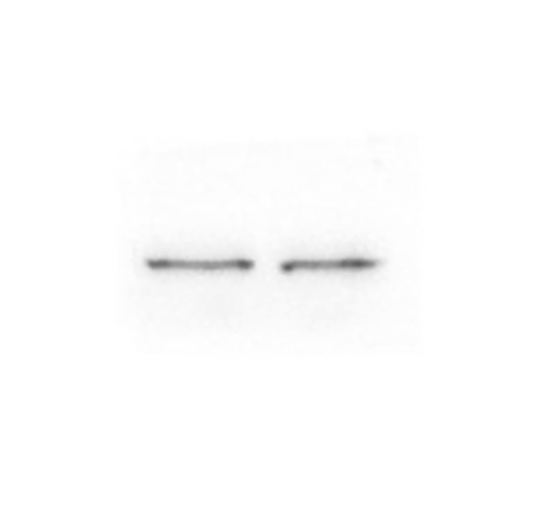

Supplement: Figure 6—source data 1. [file elife-95815-fig6-data1.zip › Figure 6—Source Data 1/Figure 6C Raw WB data/Figure 6C E 2-day C-HSCB.tif]

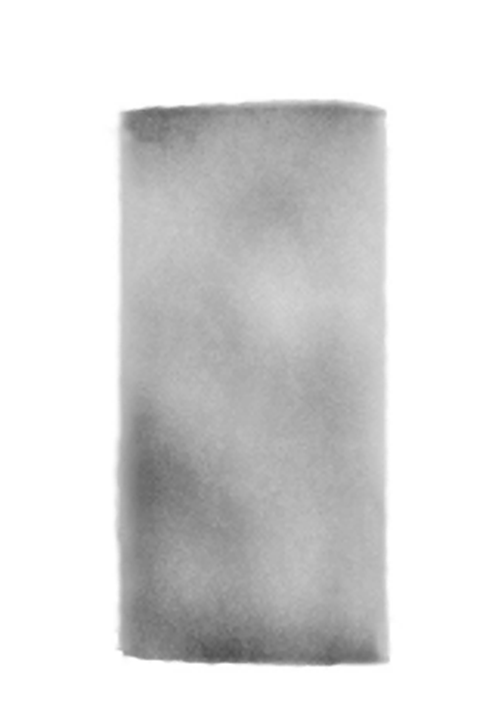

Supplement: Figure 6—source data 1. [file elife-95815-fig6-data1.zip › Figure 6—Source Data 1/Figure 6C Raw WB data/Figure 6C E 2-day IgG.tif]

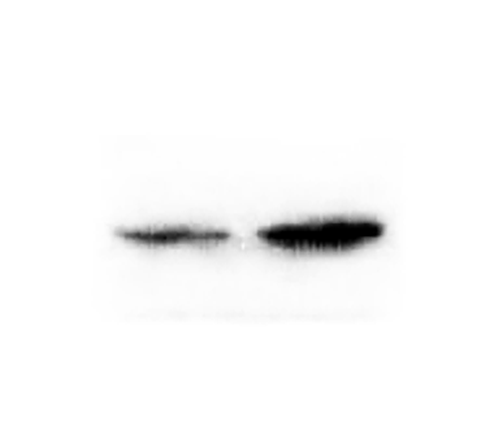

Supplement: Figure 6—source data 1. [file elife-95815-fig6-data1.zip › Figure 6—Source Data 1/Figure 6C Raw WB data/Figure 6C E 2-day TACC3.tif]

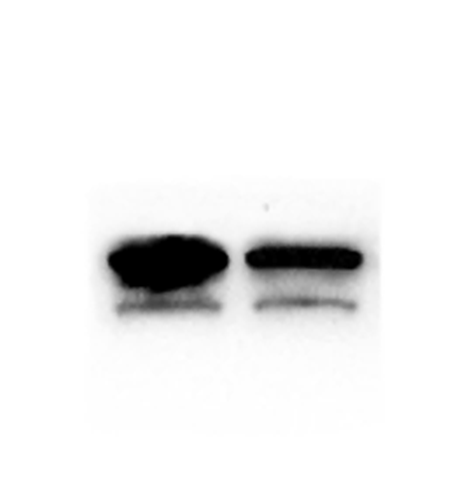

Supplement: Figure 6—source data 1. [file elife-95815-fig6-data1.zip › Figure 6—Source Data 1/Figure 6C Raw WB data/Figure 6C E 2-day pan-phospho for C-HSCB.tif]

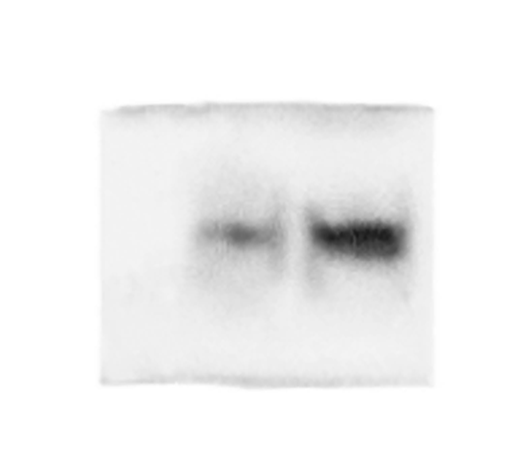

Supplement: Figure 6—source data 1. [file elife-95815-fig6-data1.zip › Figure 6—Source Data 1/Figure 6C Raw WB data/Figure 6C E 2-day pan-phospho for TACC3.tif]

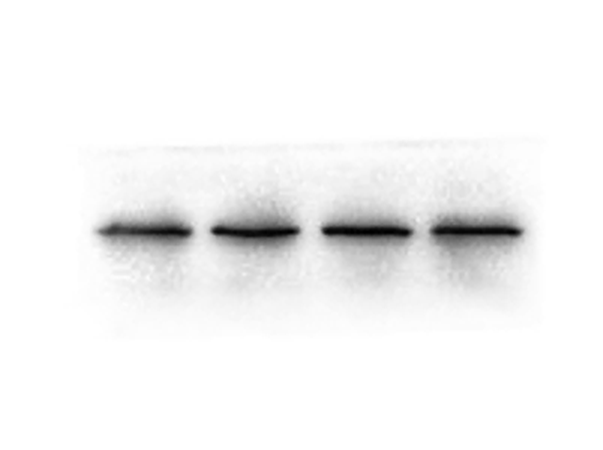

Supplement: Figure 6—source data 1. [file elife-95815-fig6-data1.zip › Figure 6—Source Data 1/Figure 6C Raw WB data/Figure 6C K562 C-HSCB.tif]

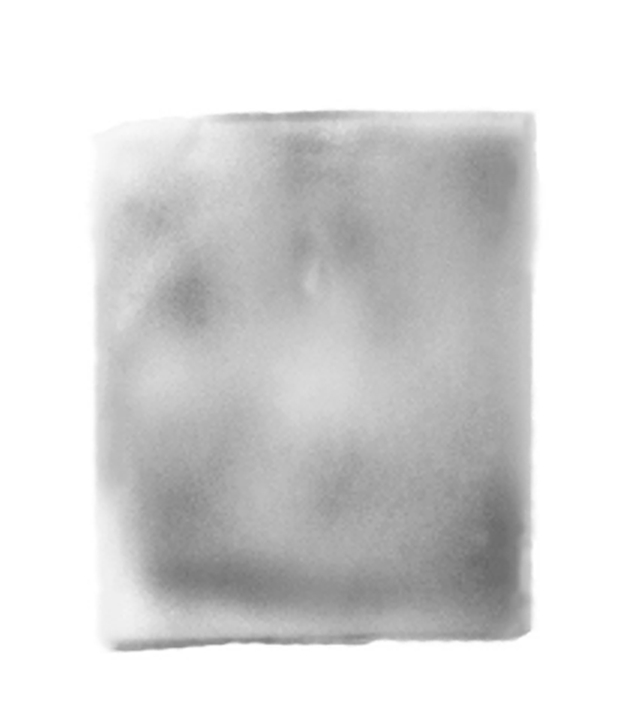

Supplement: Figure 6—source data 1. [file elife-95815-fig6-data1.zip › Figure 6—Source Data 1/Figure 6C Raw WB data/Figure 6C K562 IgG.tif]

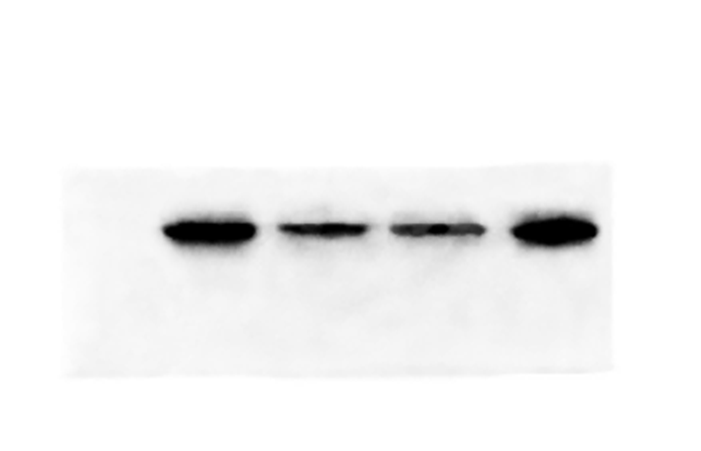

Supplement: Figure 6—source data 1. [file elife-95815-fig6-data1.zip › Figure 6—Source Data 1/Figure 6C Raw WB data/Figure 6C K562 TACC3.tif]

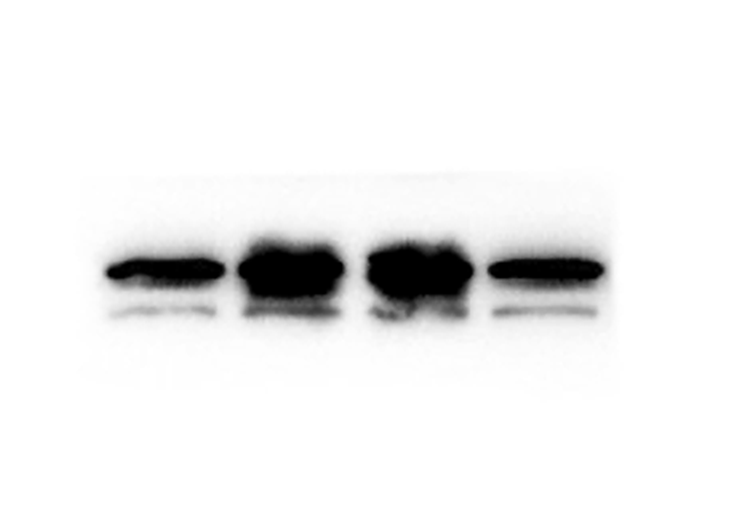

Supplement: Figure 6—source data 1. [file elife-95815-fig6-data1.zip › Figure 6—Source Data 1/Figure 6C Raw WB data/Figure 6C K562 pan-phospho for C-HSCB.tif]

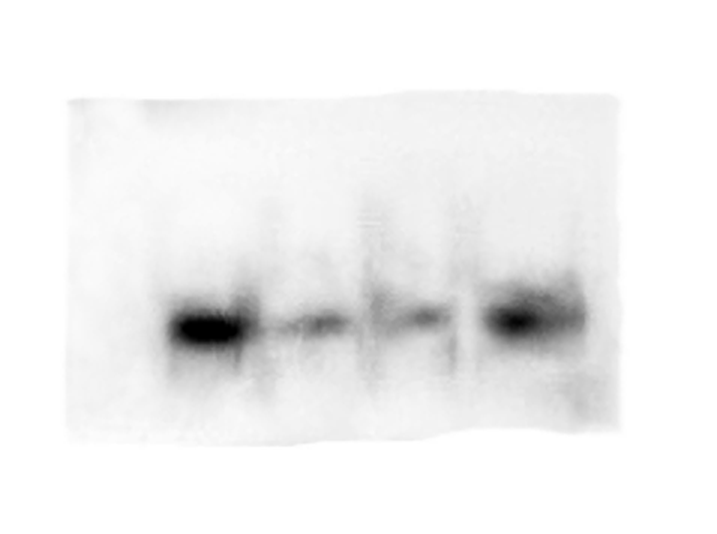

Supplement: Figure 6—source data 1. [file elife-95815-fig6-data1.zip › Figure 6—Source Data 1/Figure 6C Raw WB data/Figure 6C K562 pan-phospho for TACC3.tif]

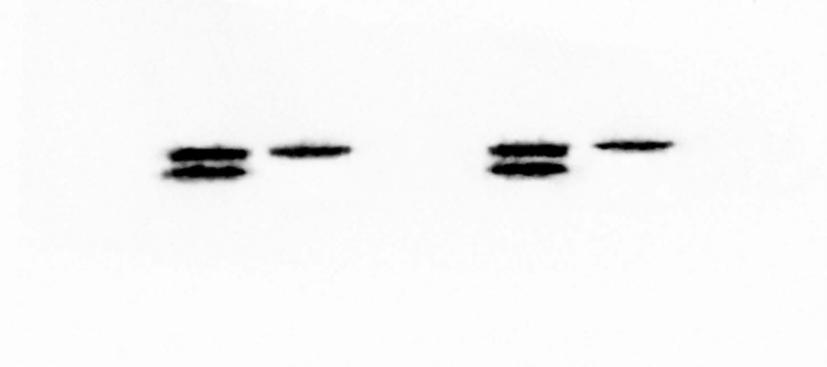

Supplement: Figure 6—source data 1. [file elife-95815-fig6-data1.zip › Figure 6—Source Data 1/Figure 6D Raw WB data/Figure 6D E 2-day HSCB.tif]

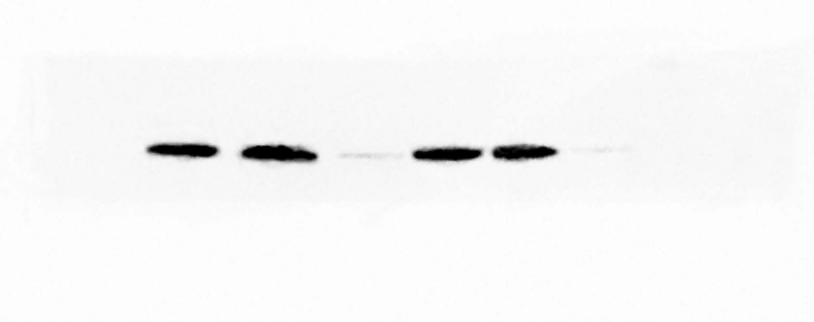

Supplement: Figure 6—source data 1. [file elife-95815-fig6-data1.zip › Figure 6—Source Data 1/Figure 6D Raw WB data/Figure 6D E 2-day PIK3R1.tif]

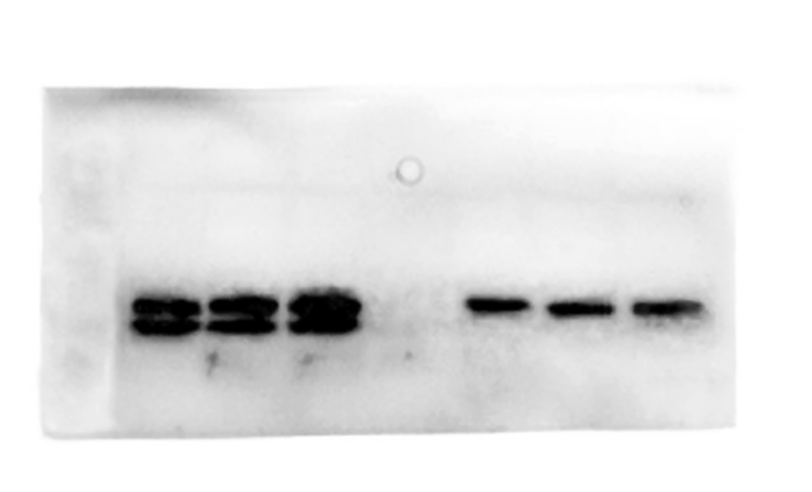

Supplement: Figure 6—source data 1. [file elife-95815-fig6-data1.zip › Figure 6—Source Data 1/Figure 6D Raw WB data/Figure 6D K562 HSCB.tif]

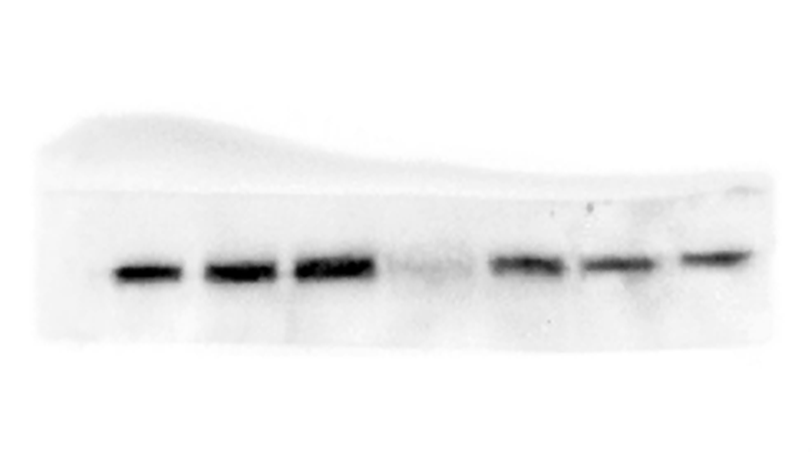

Supplement: Figure 6—source data 1. [file elife-95815-fig6-data1.zip › Figure 6—Source Data 1/Figure 6D Raw WB data/Figure 6D K562 PIK3R1.tif]

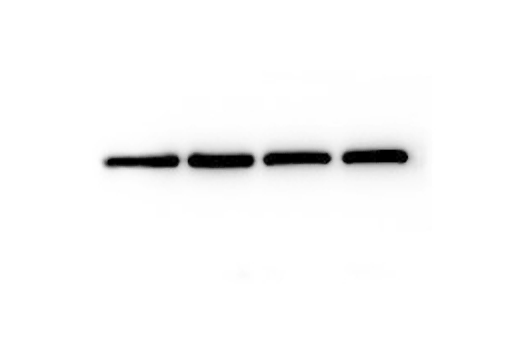

Supplement: Figure 6—source data 1. [file elife-95815-fig6-data1.zip › Figure 6—Source Data 1/Figure 6E Raw WB data/Figure 6E GAPDH.tif]

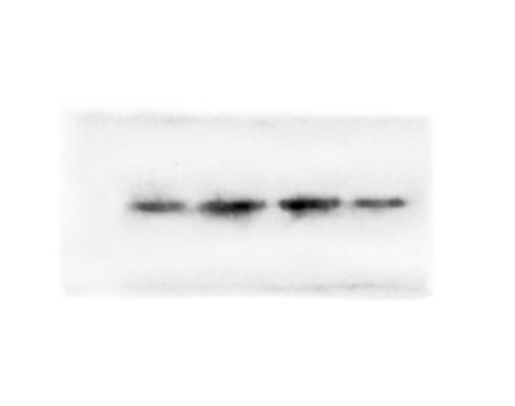

Supplement: Figure 6—source data 1. [file elife-95815-fig6-data1.zip › Figure 6—Source Data 1/Figure 6E Raw WB data/Figure 6E GYPA.tif]

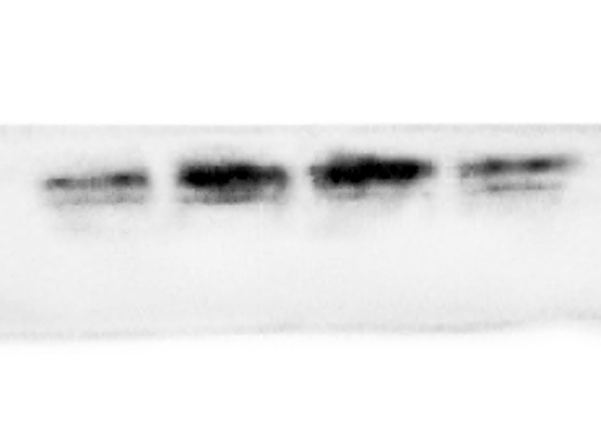

Supplement: Figure 6—source data 1. [file elife-95815-fig6-data1.zip › Figure 6—Source Data 1/Figure 6E Raw WB data/Figure 6E SPTA1.tif]

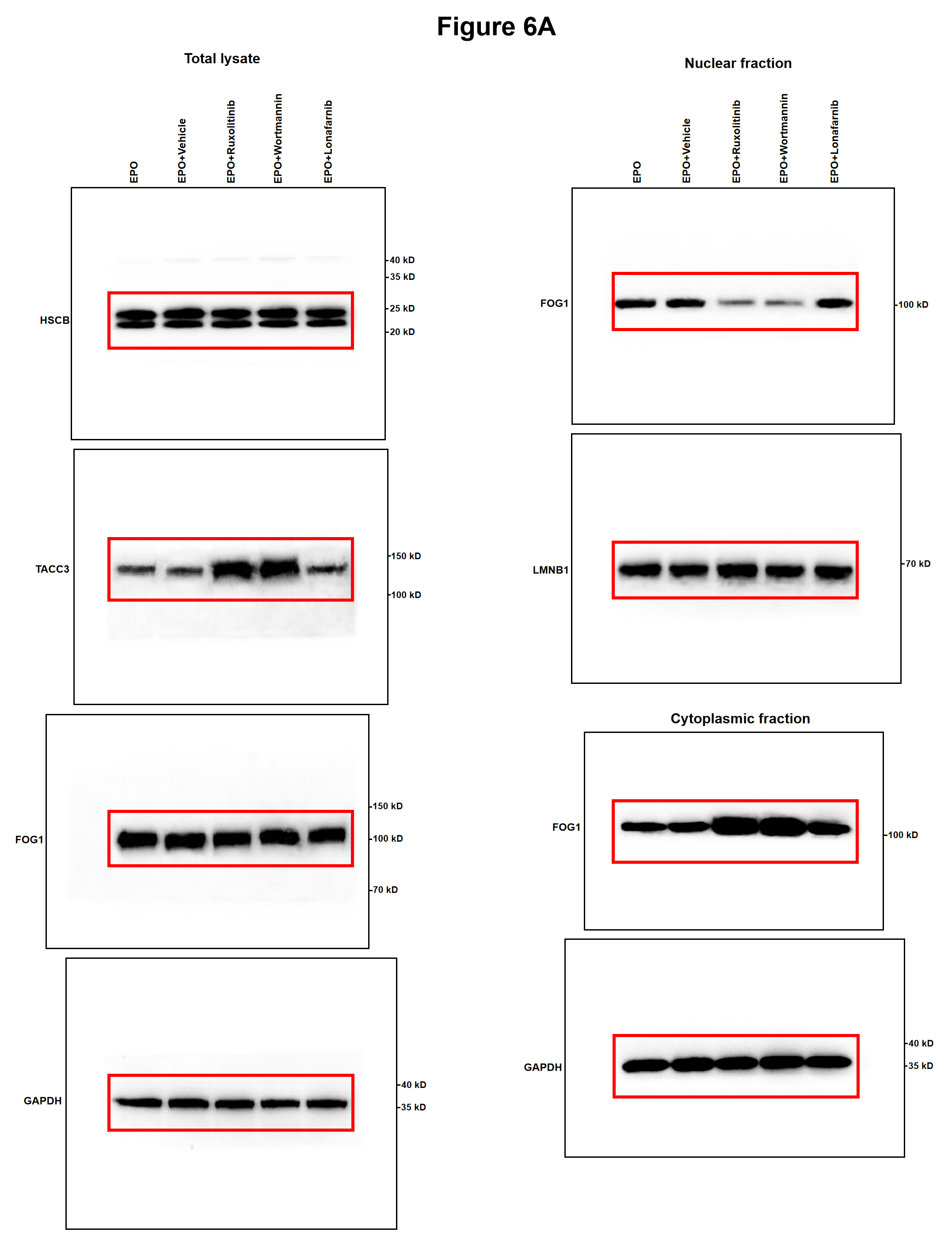

Supplement: Figure 6—source data 1. [file elife-95815-fig6-data1.zip › Figure 6—Source Data 1/Labelled WB data/Source blot data for Figure 6A.tif]

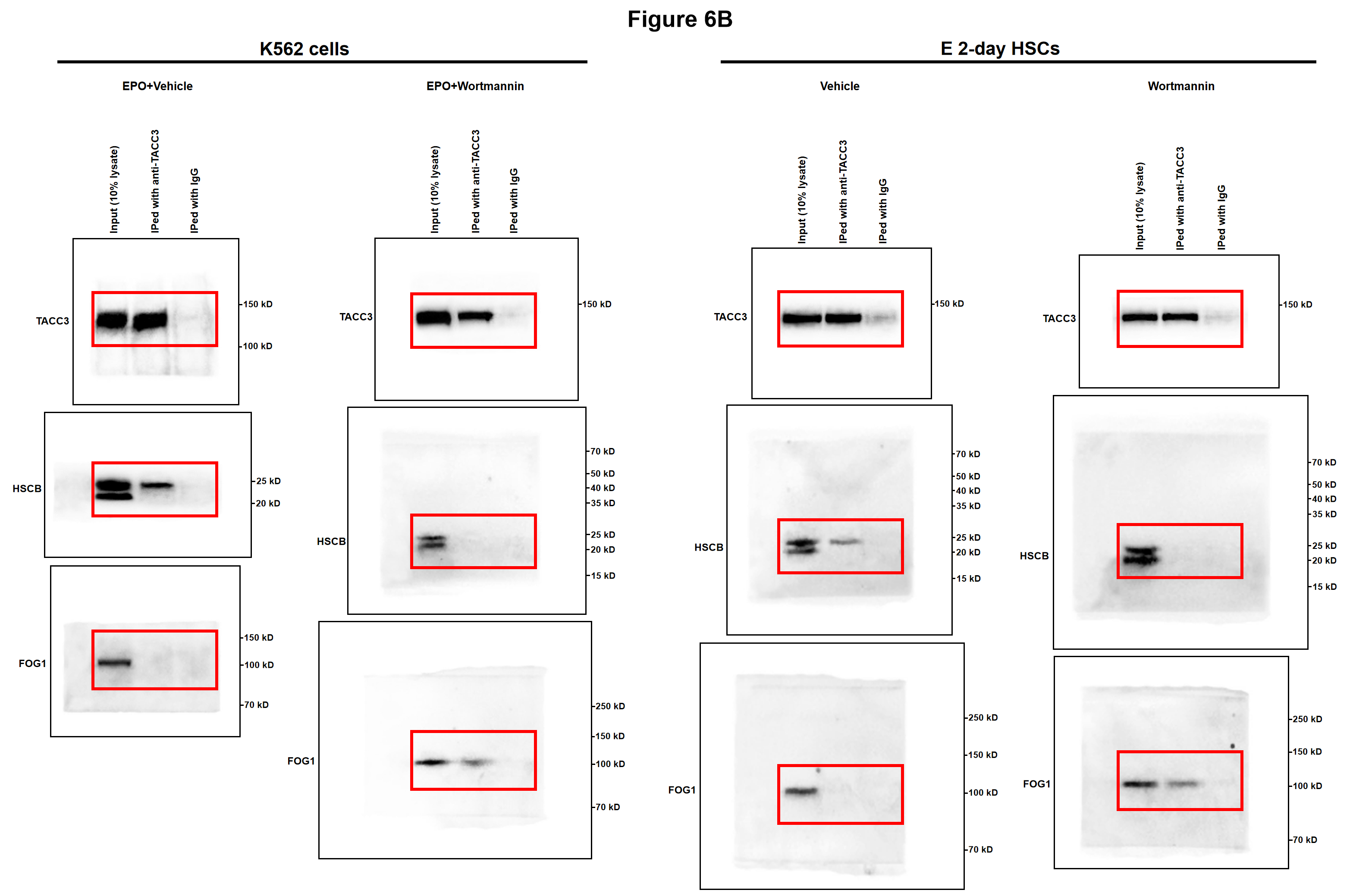

Supplement: Figure 6—source data 1. [file elife-95815-fig6-data1.zip › Figure 6—Source Data 1/Labelled WB data/Source blot data for Figure 6B.tif]

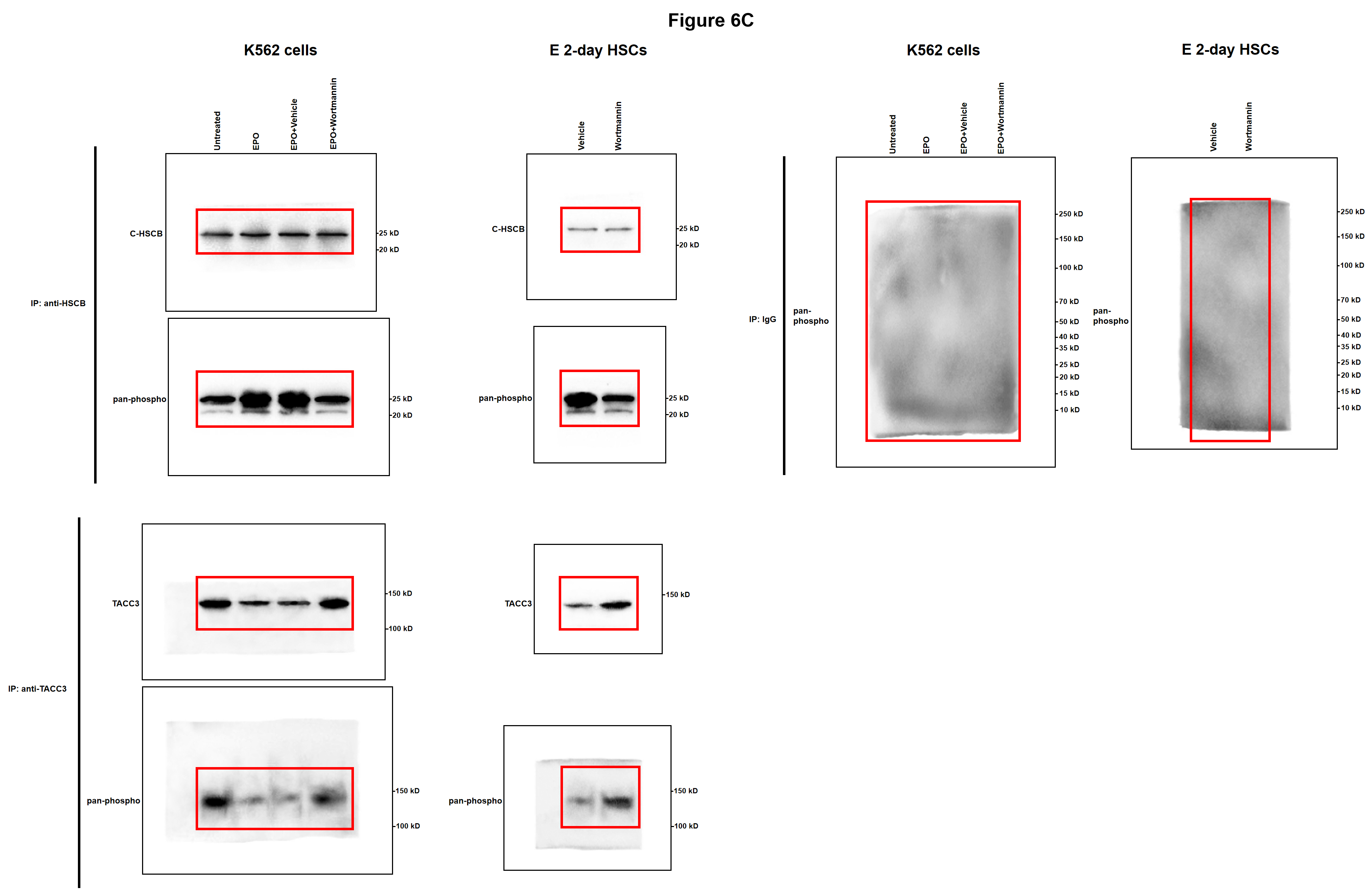

Supplement: Figure 6—source data 1. [file elife-95815-fig6-data1.zip › Figure 6—Source Data 1/Labelled WB data/Source blot data for Figure 6C.tif]

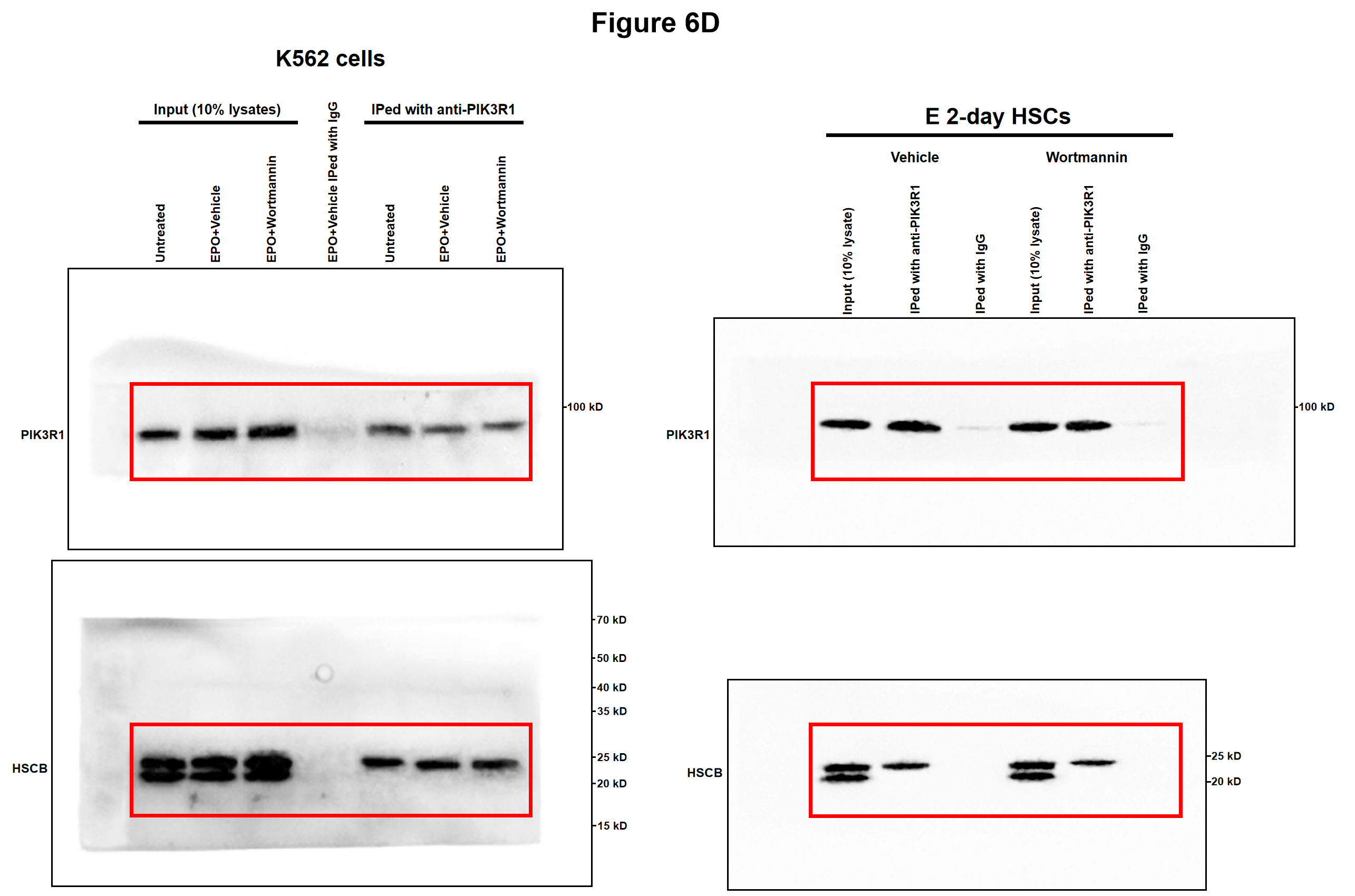

Supplement: Figure 6—source data 1. [file elife-95815-fig6-data1.zip › Figure 6—Source Data 1/Labelled WB data/Source blot data for Figure 6D.tif]

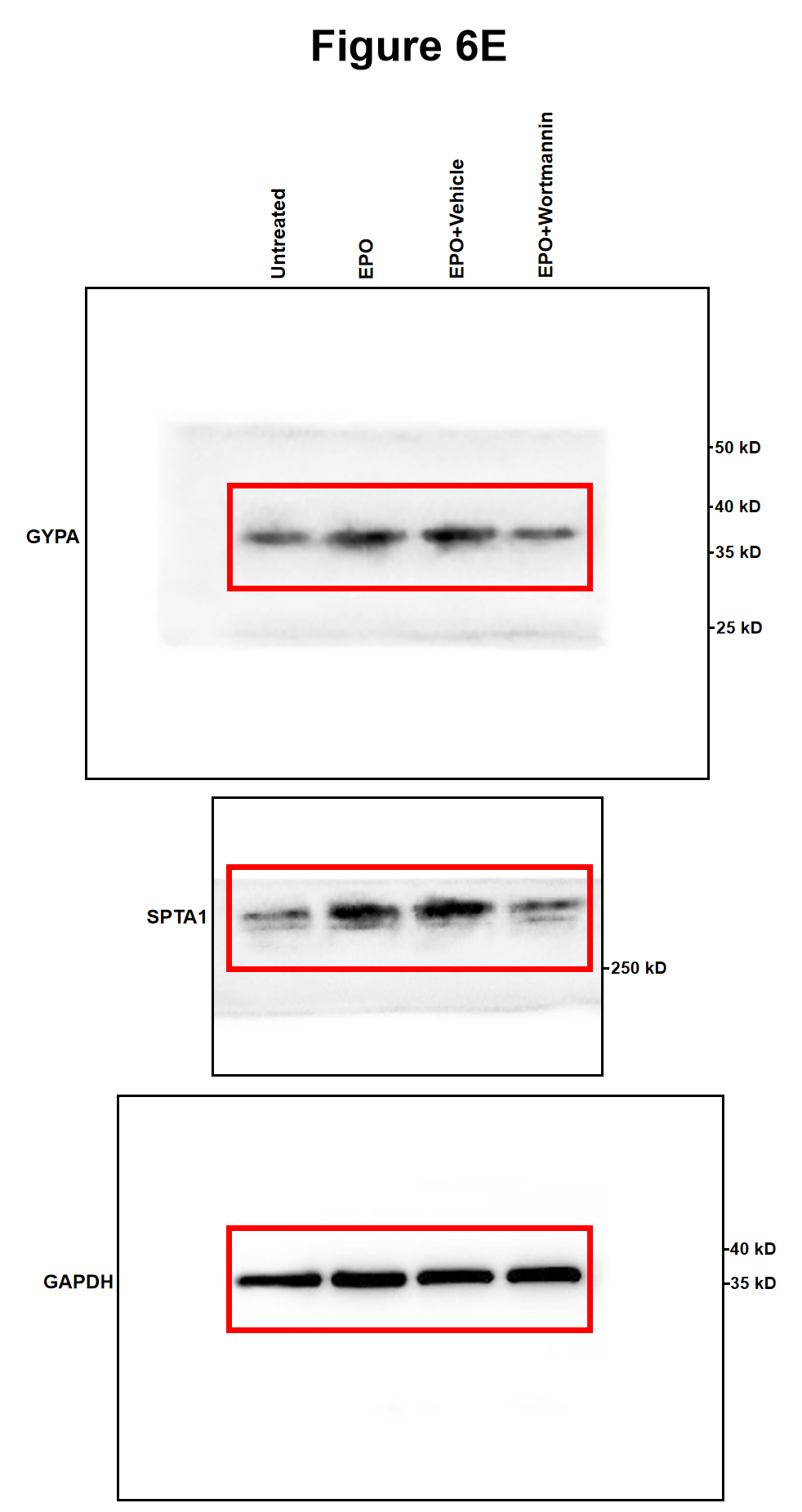

Supplement: Figure 6—source data 1. [file elife-95815-fig6-data1.zip › Figure 6—Source Data 1/Labelled WB data/Source blot data for Figure 6E.tif]

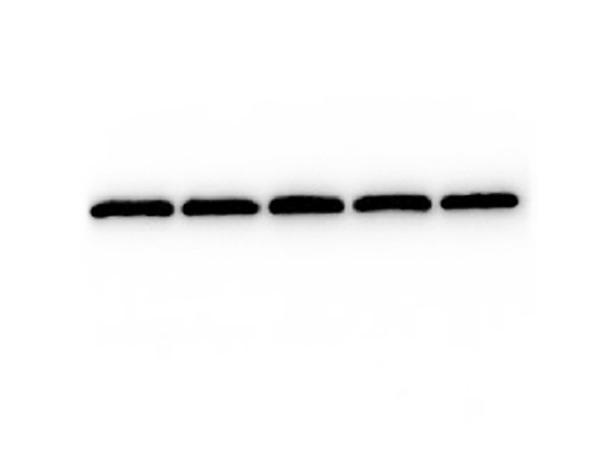

Supplement: Figure 6—figure supplement 1—source data 1. [file elife-95815-fig6-figsupp1-data1.zip › Figure 6–Figure Supplement 1–Source Data 1/Figure 6—figure supplement 1 Raw WB data/Figure 6—figure supplement 1 GAPDH.tif]

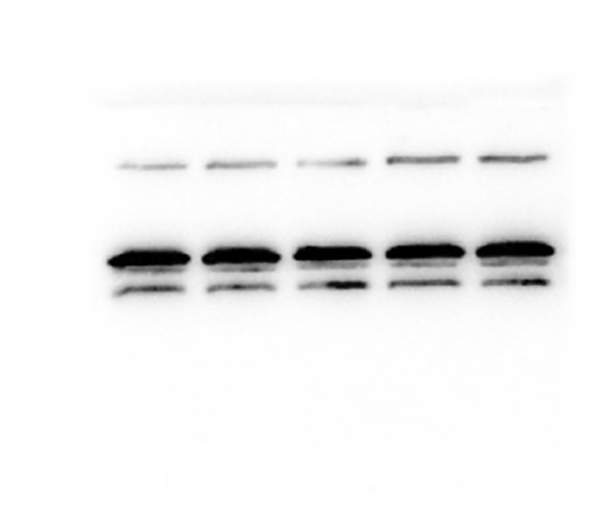

Supplement: Figure 6—figure supplement 1—source data 1. [file elife-95815-fig6-figsupp1-data1.zip › Figure 6–Figure Supplement 1–Source Data 1/Figure 6—figure supplement 1 Raw WB data/Figure 6—figure supplement 1 MEK1 2.tif]

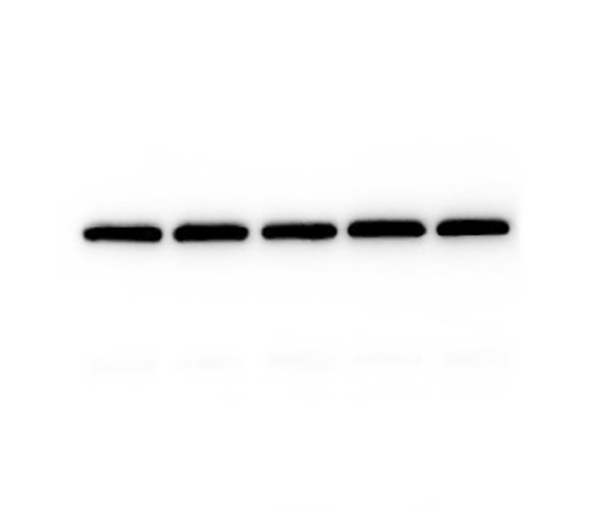

Supplement: Figure 6—figure supplement 1—source data 1. [file elife-95815-fig6-figsupp1-data1.zip › Figure 6–Figure Supplement 1–Source Data 1/Figure 6—figure supplement 1 Raw WB data/Figure 6—figure supplement 1 STAT5.tif]

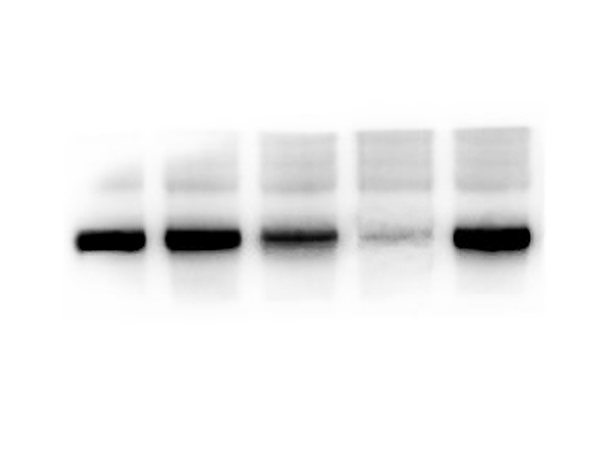

Supplement: Figure 6—figure supplement 1—source data 1. [file elife-95815-fig6-figsupp1-data1.zip › Figure 6–Figure Supplement 1–Source Data 1/Figure 6—figure supplement 1 Raw WB data/Figure 6—figure supplement 1 p-AKT.tif]

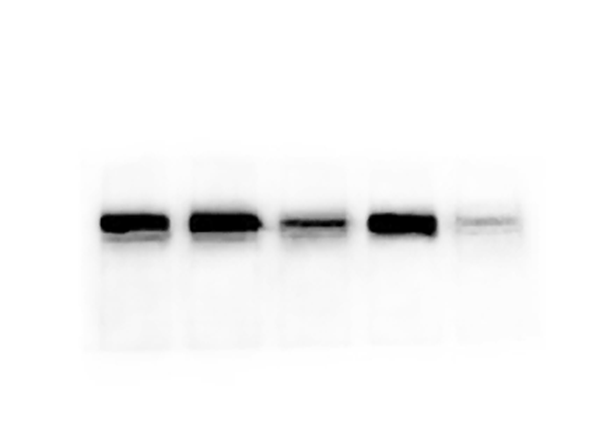

Supplement: Figure 6—figure supplement 1—source data 1. [file elife-95815-fig6-figsupp1-data1.zip › Figure 6–Figure Supplement 1–Source Data 1/Figure 6—figure supplement 1 Raw WB data/Figure 6—figure supplement 1 p-MEK1 2.tif]

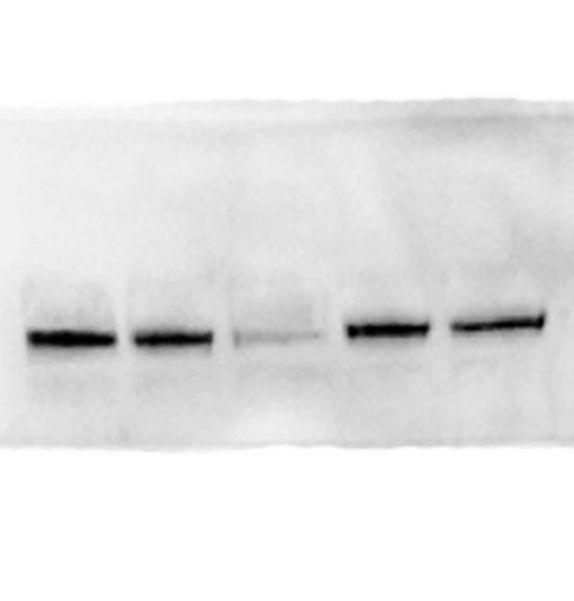

Supplement: Figure 6—figure supplement 1—source data 1. [file elife-95815-fig6-figsupp1-data1.zip › Figure 6–Figure Supplement 1–Source Data 1/Figure 6—figure supplement 1 Raw WB data/Figure 6—figure supplement 1 p-STAT5.tif]

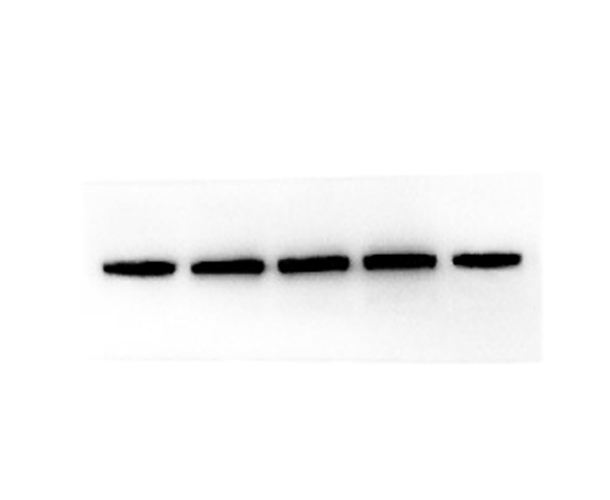

Supplement: Figure 6—figure supplement 1—source data 1. [file elife-95815-fig6-figsupp1-data1.zip › Figure 6–Figure Supplement 1–Source Data 1/Figure 6—figure supplement 1 Raw WB data/Figure 6—figure supplement 1 pan-AKT.tif]

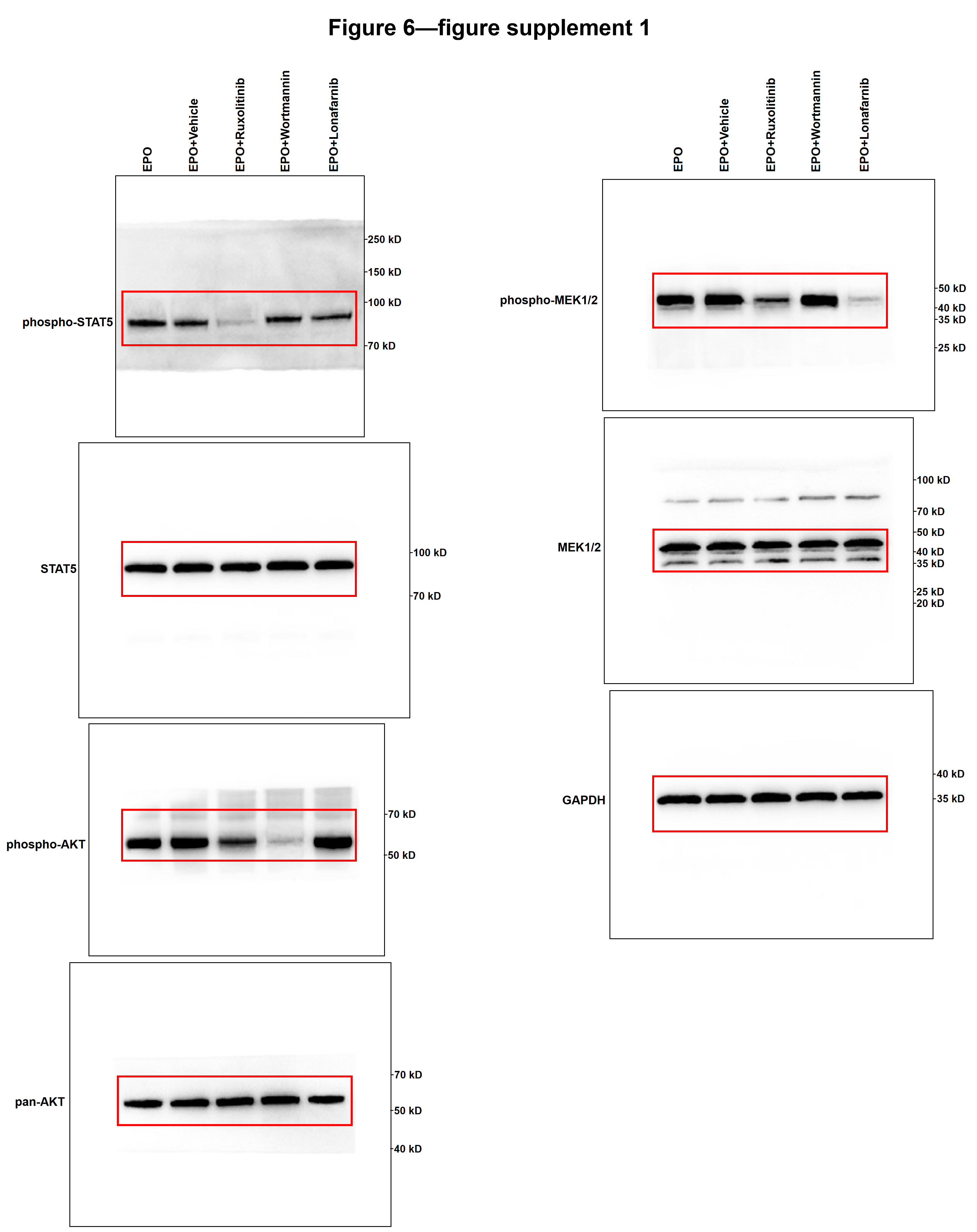

Supplement: Figure 6—figure supplement 1—source data 1. [file elife-95815-fig6-figsupp1-data1.zip › Figure 6–Figure Supplement 1–Source Data 1/Labelled blot data for Figure 6—figure supplement 1.tif]

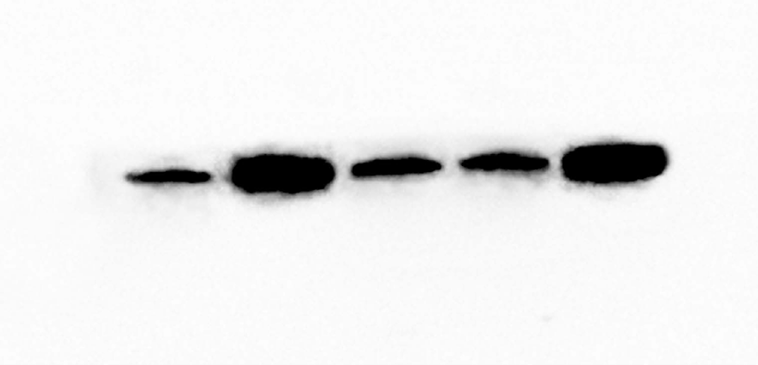

Supplement: Figure 7—source data 1. [file elife-95815-fig7-data1.zip › Figure 7—Source Data 1/Figure 7E Raw WB data/Figure 7E C-FOG1.tif]

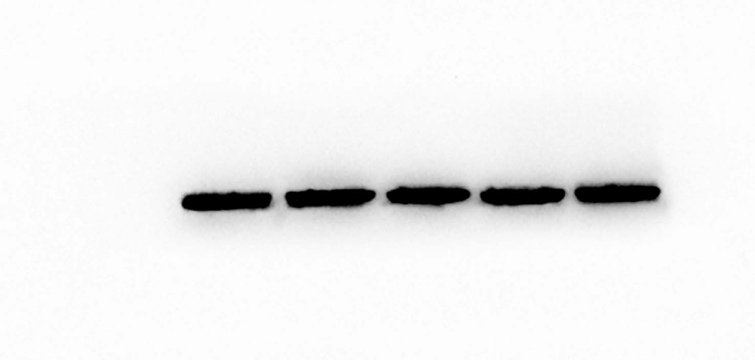

Supplement: Figure 7—source data 1. [file elife-95815-fig7-data1.zip › Figure 7—Source Data 1/Figure 7E Raw WB data/Figure 7E C-GAPDH.tif]

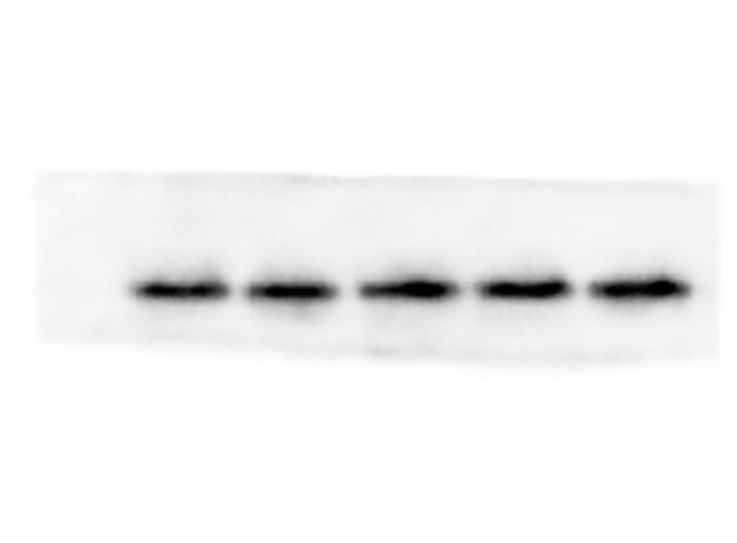

Supplement: Figure 7—source data 1. [file elife-95815-fig7-data1.zip › Figure 7—Source Data 1/Figure 7E Raw WB data/Figure 7E FOG1.tif]

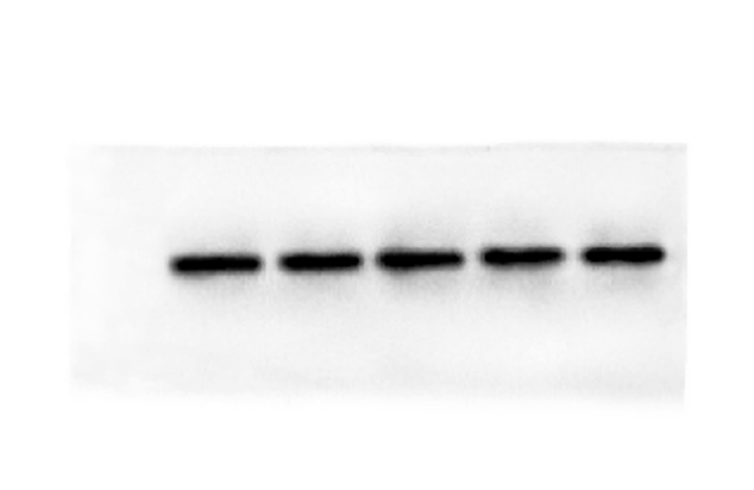

Supplement: Figure 7—source data 1. [file elife-95815-fig7-data1.zip › Figure 7—Source Data 1/Figure 7E Raw WB data/Figure 7E GAPDH.tif]

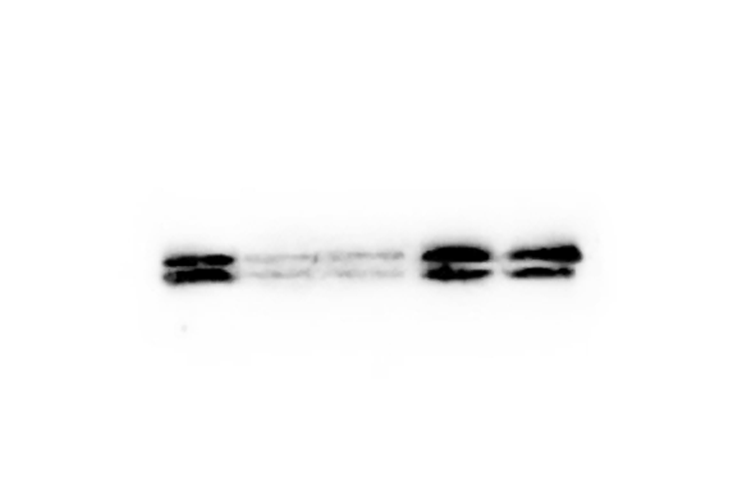

Supplement: Figure 7—source data 1. [file elife-95815-fig7-data1.zip › Figure 7—Source Data 1/Figure 7E Raw WB data/Figure 7E HSCB.tif]

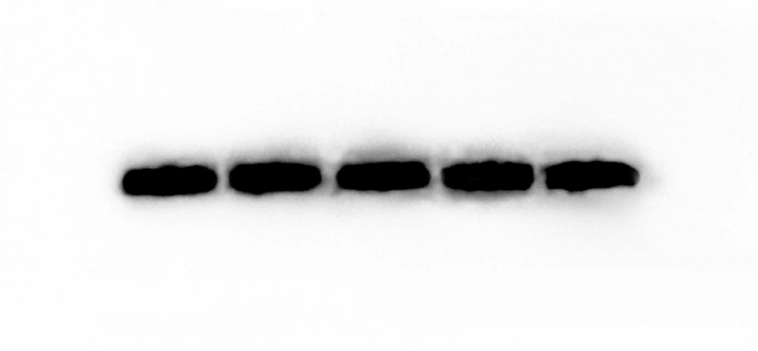

Supplement: Figure 7—source data 1. [file elife-95815-fig7-data1.zip › Figure 7—Source Data 1/Figure 7E Raw WB data/Figure 7E LMNB1.tif]

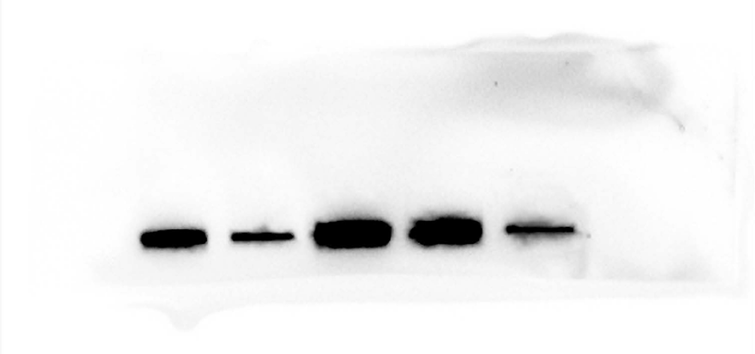

Supplement: Figure 7—source data 1. [file elife-95815-fig7-data1.zip › Figure 7—Source Data 1/Figure 7E Raw WB data/Figure 7E N-FOG1.tif]

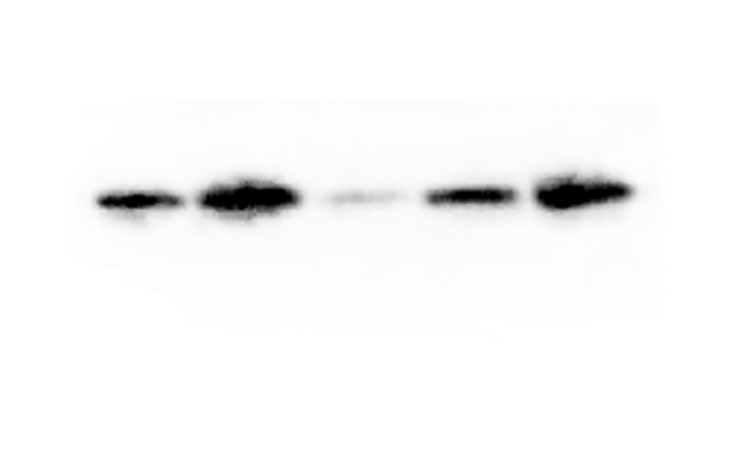

Supplement: Figure 7—source data 1. [file elife-95815-fig7-data1.zip › Figure 7—Source Data 1/Figure 7E Raw WB data/Figure 7E TACC3.tif]

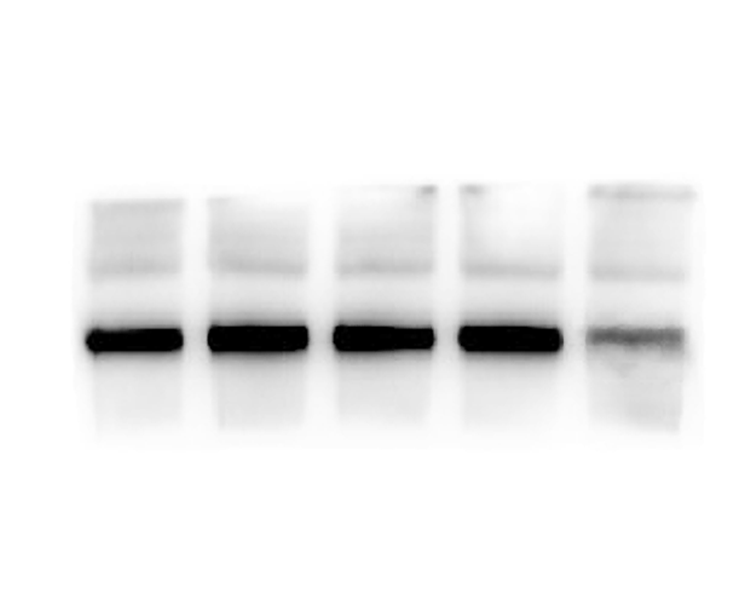

Supplement: Figure 7—source data 1. [file elife-95815-fig7-data1.zip › Figure 7—Source Data 1/Figure 7E Raw WB data/Figure 7E p-AKT.tif]

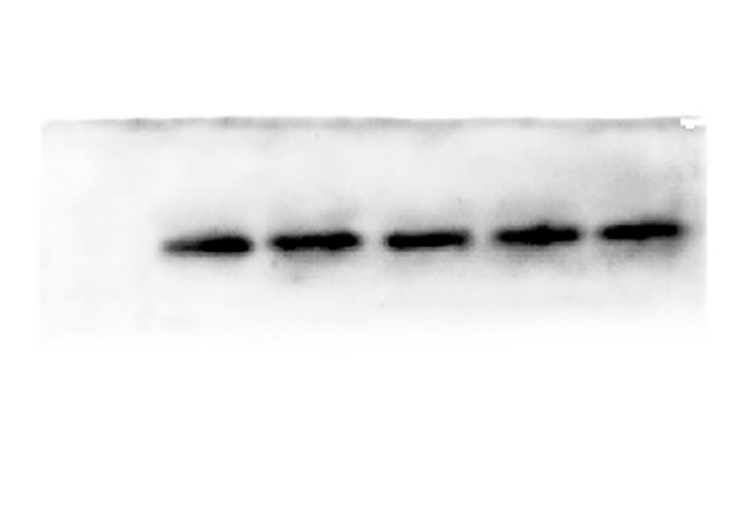

Supplement: Figure 7—source data 1. [file elife-95815-fig7-data1.zip › Figure 7—Source Data 1/Figure 7E Raw WB data/Figure 7E pan-AKT.tif]

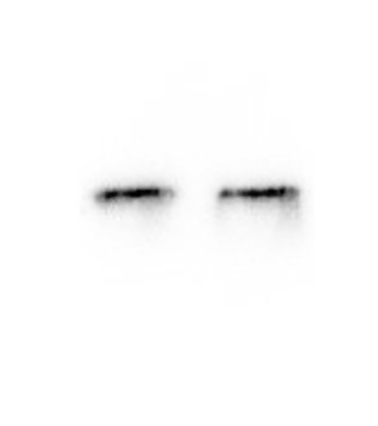

Supplement: Figure 7—source data 1. [file elife-95815-fig7-data1.zip › Figure 7—Source Data 1/Figure 7F Raw WB data/Figure 7F C-HSCB.tif]

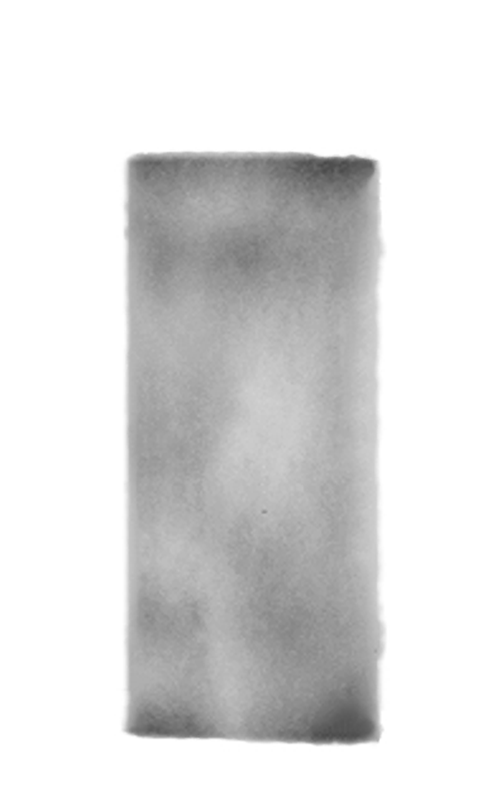

Supplement: Figure 7—source data 1. [file elife-95815-fig7-data1.zip › Figure 7—Source Data 1/Figure 7F Raw WB data/Figure 7F IgG.tif]

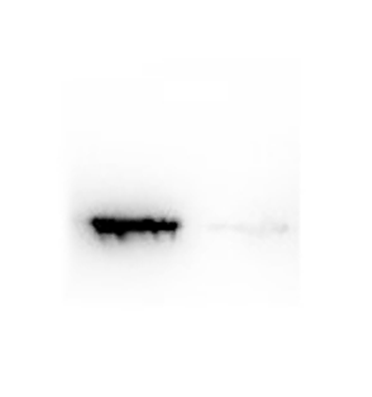

Supplement: Figure 7—source data 1. [file elife-95815-fig7-data1.zip › Figure 7—Source Data 1/Figure 7F Raw WB data/Figure 7F TACC3.tif]

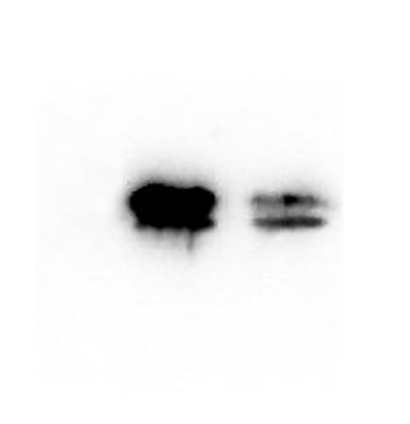

Supplement: Figure 7—source data 1. [file elife-95815-fig7-data1.zip › Figure 7—Source Data 1/Figure 7F Raw WB data/Figure 7F pan-phospho for C-HSCB.tif]

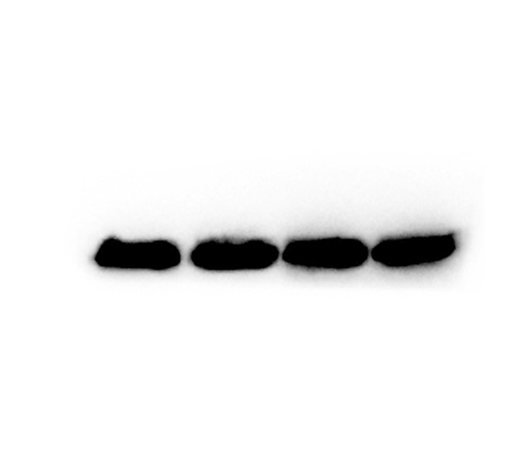

Supplement: Figure 7—source data 1. [file elife-95815-fig7-data1.zip › Figure 7—Source Data 1/Figure 7G Raw WB data/Figure 7G LMNB1.tif]

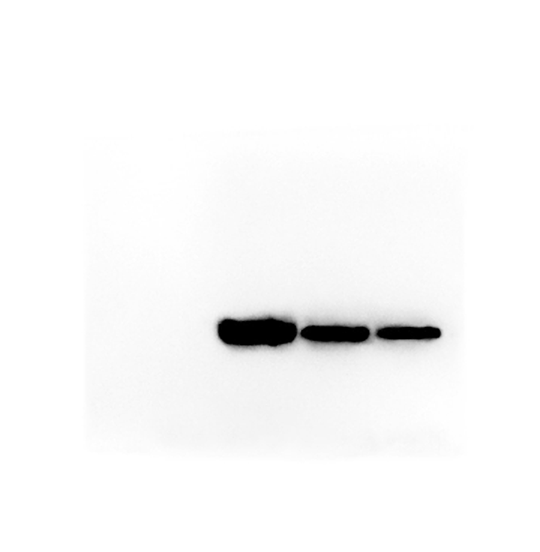

Supplement: Figure 7—source data 1. [file elife-95815-fig7-data1.zip › Figure 7—Source Data 1/Figure 7G Raw WB data/Figure 7G N-FOG1.tif]

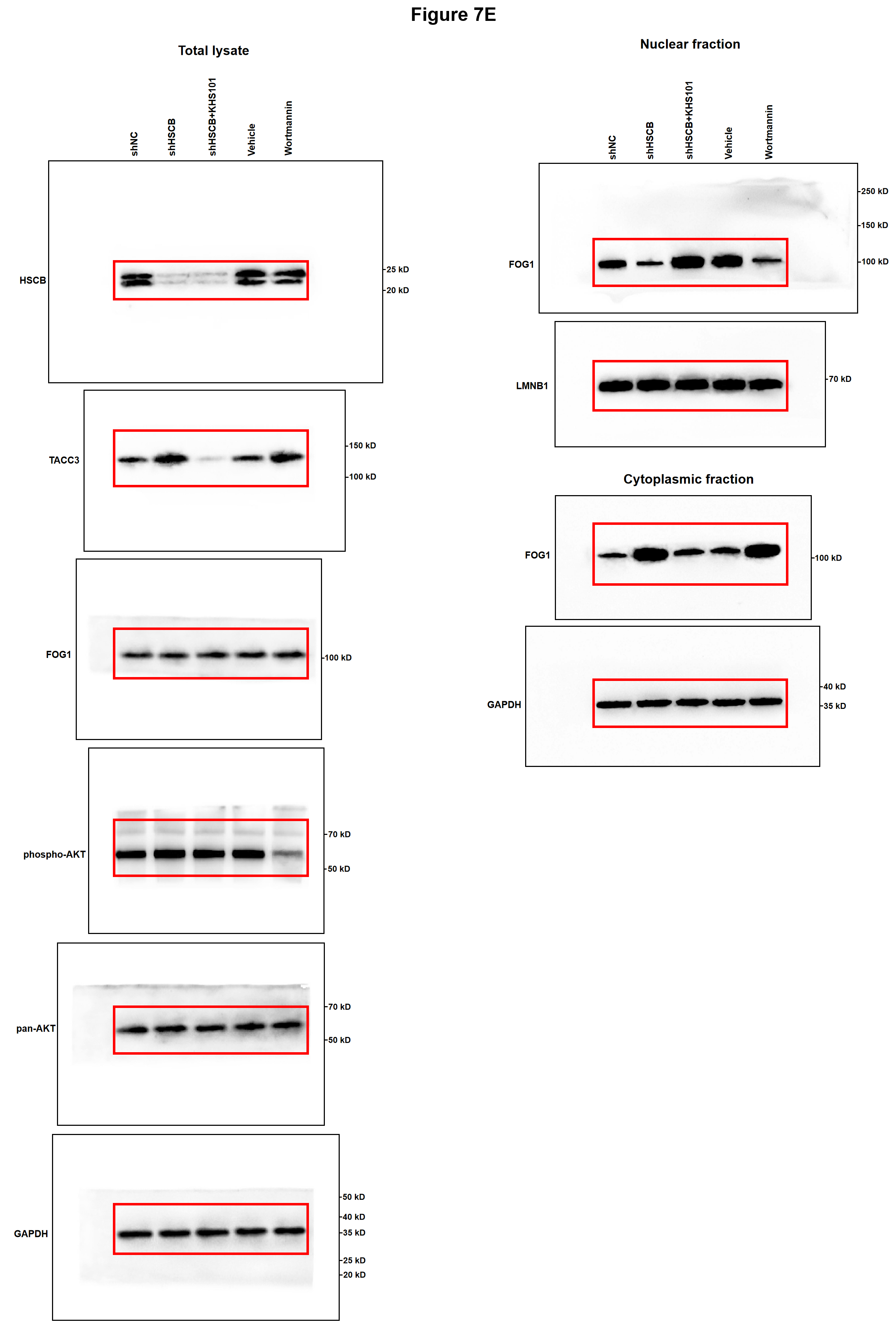

Supplement: Figure 7—source data 1. [file elife-95815-fig7-data1.zip › Figure 7—Source Data 1/Labelled WB data/Source blot data for Figure 7E.tif]

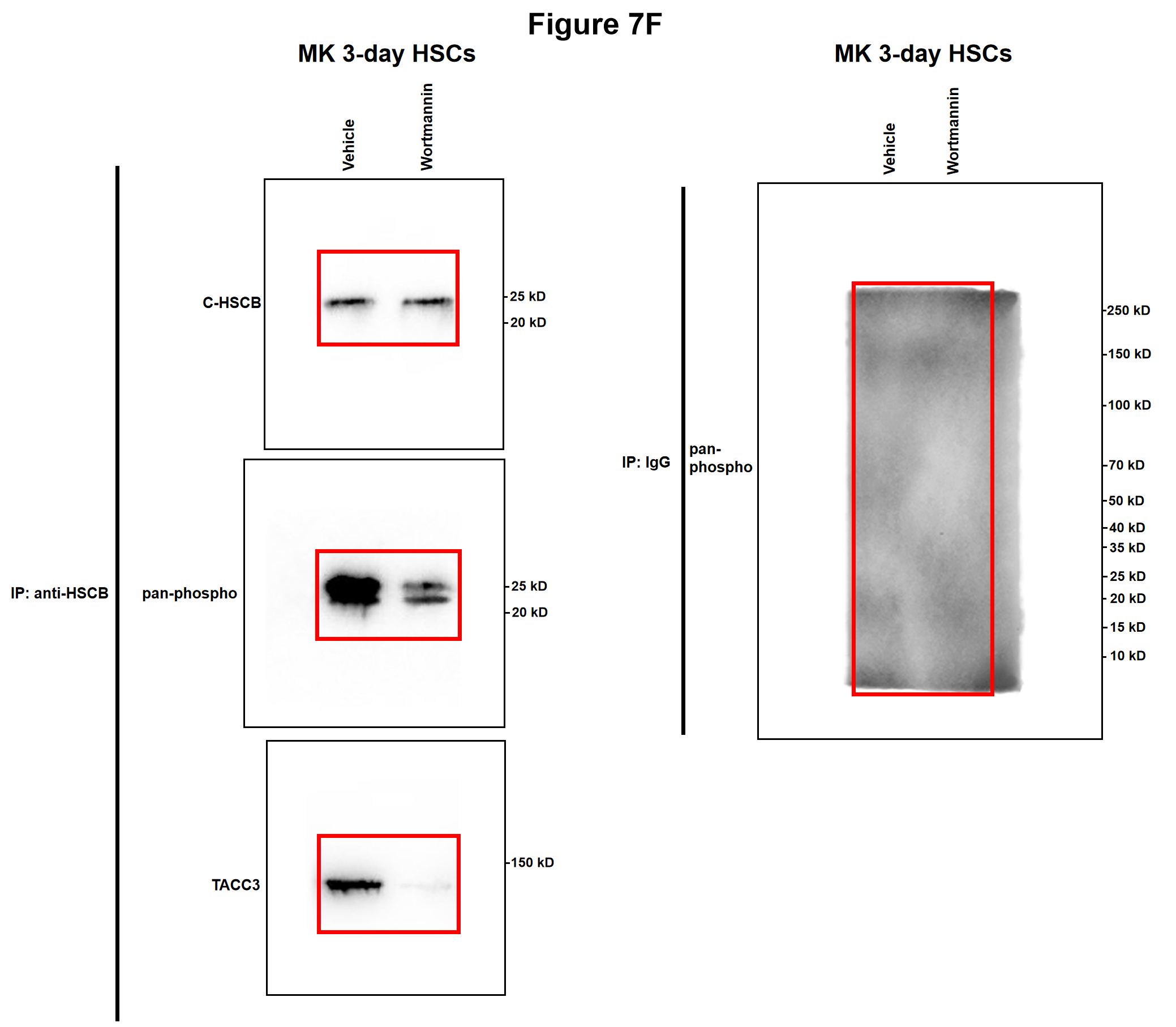

Supplement: Figure 7—source data 1. [file elife-95815-fig7-data1.zip › Figure 7—Source Data 1/Labelled WB data/Source blot data for Figure 7F.tif]

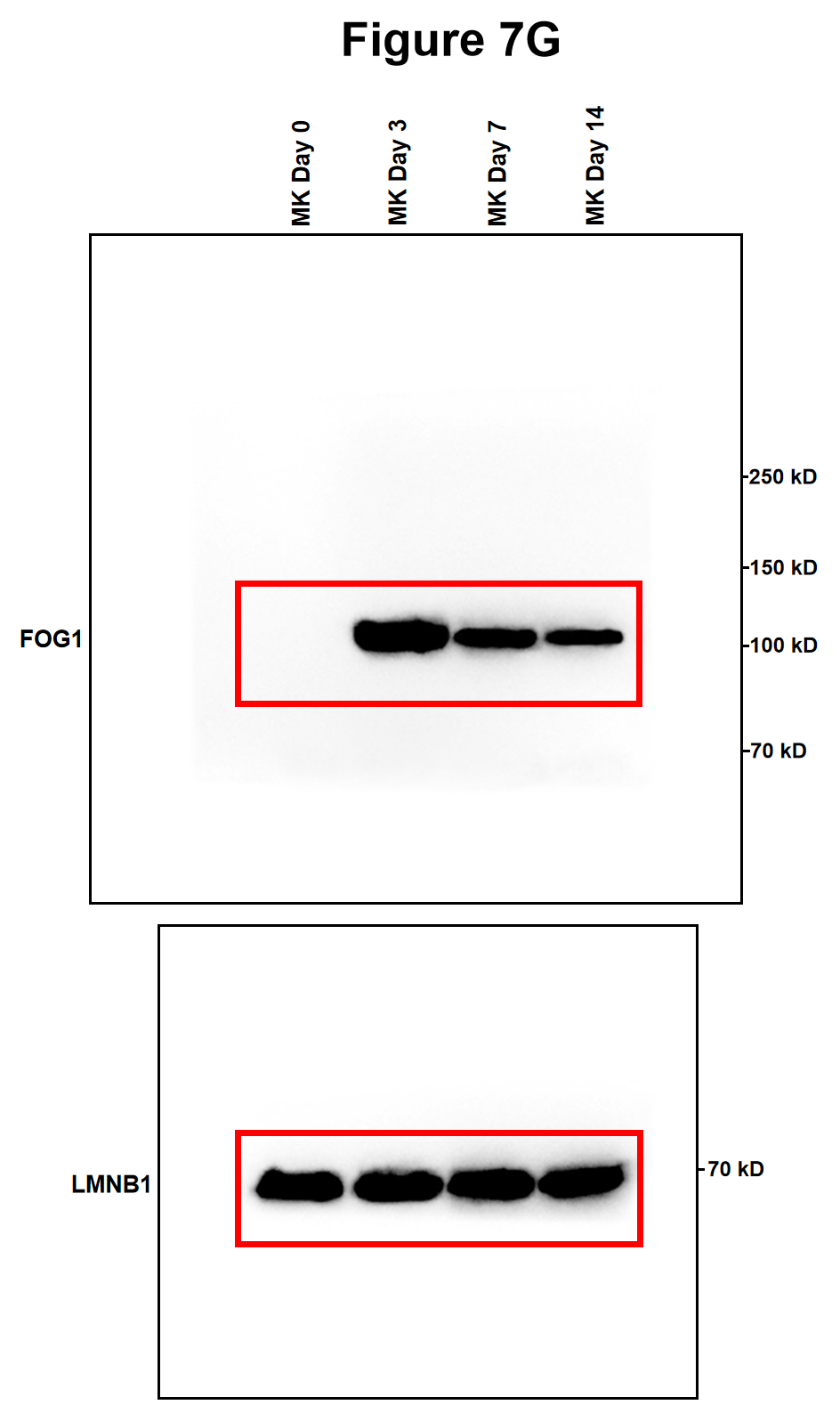

Supplement: Figure 7—source data 1. [file elife-95815-fig7-data1.zip › Figure 7—Source Data 1/Labelled WB data/Source blot data for Figure 7G.tif]
